# Determining doses for backfill cohorts based on patient-reported outcome

**DOI:** 10.1186/s12874-024-02398-w

**Published:** 2024-11-08

**Authors:** Xin Chen, Jingyi Zhang, Bosheng Li, Fangrong Yan

**Affiliations:** Department of Biostatistics, China Pharmaceutical University, Nanjing, China

**Keywords:** Backfill cohorts, Quality of life, Phase I clinical trials, Dose optimization

## Abstract

**Background:**

Incorporating backfill cohorts in phase I oncology trials is a recently developed strategy for dose optimization. However, the efficacy assessment window is long in general, causing a lag in identifying ineffective doses and more patients being backfilled to those doses. There is necessity to investigate how to use patient-reported outcomes (PRO) to determine doses for backfill cohorts.

**Methods:**

We propose a unified Bayesian design framework, called ‘Backfill-QoL’, to utilize patient-reported quality of life (QoL) data into phase I oncology trials with backfill cohorts, including methods for trial monitoring, algorithm for dose-finding, and criteria for dose selection. Simulation studies and sensitivity analyses are conducted to evaluate the proposed Backfill-QoL design.

**Results:**

The simulation studies demonstrate that the Backfill-QoL design is more efficient than traditional dose-expansion strategy, and fewer patients would be allocated to doses with unacceptable QoL profiles. A user-friendly Windows desktop application is developed and freely available for implementing the proposed design.

**Conclusions:**

The Backfill-QoL design enables continuous monitoring of safety, efficacy and QoL outcomes, and the recommended phase II dose (RP2D) can be identified in a more patient-centered perspective.

**Supplementary Information:**

The online version contains supplementary material available at 10.1186/s12874-024-02398-w.

## Background

The primary objective of phase I oncology clinical trials is to determine the maximum tolerated dose (MTD) of an investigational drug, as well as to evaluate its safety and tolerability. This ‘more is better’ paradigm is based on the assumption that higher doses will lead to better clinical outcomes (e.g., improved tumor response rates). In traditional phase I trials, it involves dose-escalation until reaching the MTD, which is the highest dose at which the drug can be administered without causing unacceptable toxicity or side effects. However, with the development of molecularly targeted agents and immunotherapies, this ‘more is better’ paradigm is challenged. Novel anti-tumor therapies often have distinct mechanisms of action and may exhibit different dose–response relationships compared to cytotoxic drugs. In some cases, higher doses do not necessarily lead to improved efficacy but instead increase the risk of toxicity [1, 2]. The optimal dose with a desired biological effect while minimizing toxicity, is likely to be lower than the MTD.

To determine the recommended phase II dose (RP2D) with a better benefit-risk balance, phase I trials often include dose-expansion cohorts at the identified MTD and/or doses below the MTD [3–6]. However, this dose-optimization strategy may require a long trial duration because the dose-expansion phase always follows the completion of the initial dose-escalation. Another type of dose-optimization design that employs the risk–benefit tradeoff to guide dose-finding has recently gained increasing attention. Typical examples include EffTox [7], BOIN-ET [8], U-BOIN [9], BOIN12 [10], and uTPI [11], among others. When the efficacy endpoint can be observed over a relatively short period of time, these designs are efficient in identifying the optimal dose. The recently developed ‘backfill cohorts’ is also a strategy for dose optimization [12–15]. It refers to the practice of enrolling additional patients into lower dose levels, which have been tested and regarded as safe, after the current dose-escalation cohort has been filled. The objective of backfilling is to gather more safety and efficacy data of lower doses [16–18]. In cases where the optimal dose is lower than the MTD, the backfill strategy may be especially effective. Furthermore, in traditional phase I trials, investigators often have to wait for patients of the current dose-escalation cohort to complete the toxicity assessment before treating the next cohort. If the backfill strategy is adopted, newly enrolled patients could then have the opportunity to be treated without having to wait for the previous patients to complete the assessment. Backfilling has the potential to improve the coherence of dose-escalation, save time by avoiding accrual interruptions, and allow more patients to have access to the investigational therapy [19].

One key question we may encounter in trials with backfilling is: How to determine doses for the backfill cohorts? It has been suggested to backfill patients to dose levels where evidence of drug activity has been observed [16–19]. However, the efficacy assessment window may be long in the context of oncology trials. Some novel therapies even have delayed treatment effects [20–22], and it may be necessary to observe whether a patient has a response at the second or third radiologic assessment. This issue may cause a lag in determining ineffective doses and may result in more patients being backfilled to those doses.

Therefore, we propose to determine doses for backfill cohorts based on patient-reported quality of life (QoL) data, which can be collected in a more timely manner than radiologic assessment. Patient-reported outcomes (PROs) are supplements to traditional safety and efficacy data, grounded on patient perceptions of treatment [23, 24], and there is convincing evidence that PROs are independent prognostic factors for overall survival [25]. In recent years, collecting PROs in early-phase clinical trials has become more and more common [26–28], but explicit use of PROs for dose optimization is still rare. Some researchers have proposed incorporating the patient-reported outcomes version of the Common Terminology Criteria for Adverse Events (PRO-CTCAE) to inform patient tolerability in dose-finding trials [29–31], but to the best of our knowledge, no one has utilized PRO to assist dose-optimization and decision making in trial designs with backfill cohorts so far. The proposed design, called ‘Backfill-QoL’, enables continuous monitoring for safety, efficacy, and QoL outcomes. Patient allocations to low doses with poor performance on QoL can be stopped early, which is more ethical. The RP2D can be identified at study conclusion based on safety, efficacy and QoL data, informing subsequent drug development in a patient-centered perspective.

## Methods

### Backfill-QoL design

#### Notations

Consider a phase I dose-escalation trial with $J$ candidate doses. The probability of dose-limiting toxicity (DLT) and tumor response for dose $j (j=1,\dots ,J)$ are denoted as ${p}_{j}$ and ${\theta }_{j}$, respectively. Let ${n}_{j}$, ${x}_{j}$, ${r}_{j}$ denote the number of patients treated with dose level $j$, the number of patients experiencing DLT at dose level $j$, and the number of patients with tumor response at dose level $j$. Both ${x}_{j}$ and ${r}_{j}$ follow binomial distributions, i.e., ${x}_{j}\sim Binomial({n}_{j},{p}_{j})$ and ${r}_{j}\sim Binomial({n}_{j},{\theta }_{j})$.

The QoL is measured by validated patient-reported outcome measures (PROMs), e.g., the Functional Assessment of Cancer Therapy-General (FACT-G) [27, 28, 32]. Let ${y}_{ij}$ denote the change of FACT-G total score (range 0–108, a higher score means better QoL) from baseline for the ${i}_{th}$ patient treated with dose $j$, which is assumed to be independent normally distributed, that is, ${y}_{ij}\sim N({\mu }_{j},{\sigma }_{j}^{2})$, where ${\mu }_{j}$ and ${\sigma }_{j}^{2}$ are the mean and variance of the FACT-G change from baseline in patients treated with dose $j$, respectively.

#### Trial monitoring

Continuous monitoring for safety, efficacy and QoL is an important feature of the Backfill-QoL design. First, for patient safety, the monitoring rule is derived based on a beta-binomial model, which has been adopted by frequently-used Bayesian model-assisted phase I trial designs [33–36] and is now well known to trialists and oncologists. Specifically, the prior distribution of ${p}_{j}$ is the conjugate prior $Beta(a,b)$, and the posterior distribution of ${p}_{j}$ is $Beta(a+{x}_{j},b+{n}_{j}-{x}_{j})$. If the posterior probability.

$$\text{Pr}\left(p_j>\phi_{DLT}\vert Data\right)>\varphi_T,$$

Dose $j$ and higher doses are considered overly toxic, where ${\phi }_{DLT}$ is the target DLT rate (e.g., 25%), $Data$ indicates the accumulated trial data and ${\varphi }_{T}$ is the statistical cutoff for monitoring safety. The advantage of this approach is the statistical cutoff can be adjusted according to the background of the disease, and the overdosing boundaries can be presented in advance for the convenience of use.

The method for monitoring efficacy is similar to that for monitoring safety. If the posterior probability.

$$\text{Pr}\left(\theta_j>\theta_0\vert Data\right)<\varphi_E,$$

Dose $j$ is considered to be futile, where ${\theta }_{0}$ is the lowest acceptable response rate (e.g., the response rate of the standard therapy), and ${\varphi }_{E}$ is the statistical cutoff for monitoring efficacy. The posterior probability is also tractable when using the conjugate prior distribution. Table 1 provides examples of the overdosing boundaries and futility boundaries when selecting different cutoffs.

**Table 1:** The overdosing boundaries and futility boundaries when selecting different cutoffs

Overdosing boundaries									
Cutoff		Number of patients							
		3	4	5	6	7	8	9	10
0.95	Declare overly toxic if no. of DLT ≥	3	3	3	4	4	4	5	5
0.90		2	3	3	3	4	4	4	5
0.85		2	2	3	3	3	4	4	4
0.80		2	2	2	3	3	3	4	4
Futility boundaries									
Cutoff		Number of patients							
		3	4	5	6	7	8	9	10
0.80	Declare futility if no. of response ≤	0	1	1	1	2	2	2	2
0.70		0	0	1	1	1	1	2	2
0.60		0	0	0	1	1	1	1	1
0.50		0	0	0	0	0	1	1	1

The target DLT $\phi_{DLT}$ rate is 0.25. The lowest acceptable response rate $\theta_0$ is 0.2. The prior set for each $p_j$ and $\theta_j$ is $Beta(1,1)$

In terms of monitoring QoL outcomes, if the posterior predictive probability.

$$\text{Pr}\left({\widetilde y}_j<\phi_{QoL}\vert Data\right)>\varphi_Q,$$

Dose $j$ is considered unacceptable, where ${\widetilde{y}}_{j}$ represents the change of QoL score from baseline for a future patient treated with dose $j$, ${\phi }_{QoL}$ is the maximum acceptable mean QoL deterioration (e.g., -10), ${\varphi }_{Q}$ is the statistical cutoff for monitoring QoL. When setting noninformative uniform prior distribution on $({\mu }_{j},\text{log}{\sigma }_{j}$), the posterior predictive distribution of ${\widetilde{y}}_{j}$ is the *t* distribution with location ${\overline{y} }_{j}$, scale ${(1+1/{n}_{j})}^{1/2}{s}_{j}$, and ${n}_{j}-1$ degrees of freedom:

$${\widetilde{y}}_{j}|Data\sim {t}_{{(n}_{j}-1)}\left({\overline{y} }_{j},{\left(1+\frac{1}{{n}_{j}}\right)}^\frac{1}{2}{s}_{j}\right),$$

where ${\overline{y} }_{j}$ and ${s}_{j}$ are the sample mean and sample standard deviation of QoL outcomes for patients treated with dose $j$, respectively. For the derivation of the posterior predictive distribution, the readers may refer to Sect. 3.2 of the monograph by Gelman et al. (2013) [37]. There are two main reasons for using posterior predictive probability rather than the posterior probability $\text{Pr}\left({\mu }_{j}<{\phi }_{QoL}|Data\right)$ to monitor QoL outcomes. First, the posterior predictive probability is more intuitive as it represents the proportion of patients with undesired QoL outcomes while the posterior probability just denotes the probability that ${\mu }_{j}<{\phi }_{QoL}$. In addition, the posterior distribution of ${\mu }_{j}$ does not account for the sampling variance ${\sigma }_{j}^{2}$, sometimes producing misleading results. For example, assuming the true distribution of ${y}_{ij}$ is $N(-9, 10)$, then $\text{Pr}\left({\mu }_{j}<-10|Data\right)$ will tend to 0 when the sample size is large, and one would believe that the QoL profile of dose $j$ is satisfactory. But in reality, nearly half of the patients will have undesired QoL outcomes, which may be unacceptable in some cases. The posterior predictive distribution of ${\widetilde{y}}_{j}$, however, can capture such information and thus inform decision-making more comprehensively.

Based on the closed-form solution of the posterior predictive distribution of ${\widetilde{y}}_{j}$, we propose to calibrate ${\varphi }_{Q}$ by calculating the probabilities of declaring a dose with unacceptable QoL profiles under several scenarios. It has been proved that when ${\varphi }_{Q}$, ${n}_{j}$ and $\text{Pr}({y}_{j}<{\phi }_{QoL})$ (i.e., the proportion of patient population with QoL changes lower than ${\phi }_{QoL}$) are fixed, the probability of $\text{Pr}\left({\widetilde{y}}_{j}<{\phi }_{QoL}|Data\right)>{\varphi }_{Q}$ (i.e., declaring that dose with unacceptable QoL profiles) is a constant regardless of the specific values of ${\mu }_{j}$ and ${\sigma }_{j}^{2}$ (See section A of the Additional file 1 for the proof). Due to this property, the calibration of the cutoff can be much simpler as we do not have to consider the specific values of ${\mu }_{j}$ and ${\sigma }_{j}^{2}$ but only the proportion of patient population with undesired QoL outcomes (examples are shown in Table 2).

**Table 2:** The probabilities of declaring a dose with unacceptable QoL profiles when selecting different cutoffs under several scenarios

Cutoff	Proportion of patient population with undesired QoL outcomes	Probabilities of declaring unacceptable QoL profiles (*N* = 3)	Probabilities of declaring unacceptable QoL profiles (*N* = 6)
0.5	25%	0.1214	0.0493
	50%	0.5000	0.5000
	75%	0.8786	0.9507
0.6	25%	0.0520	0.0115
	50%	0.3110	0.2556
	75%	0.7374	0.8308
0.7	25%	0.0226	0.0024
	50%	0.1712	0.0995
	75%	0.5310	0.5894

#### Dose-finding algorithm

**Fig. 1:**
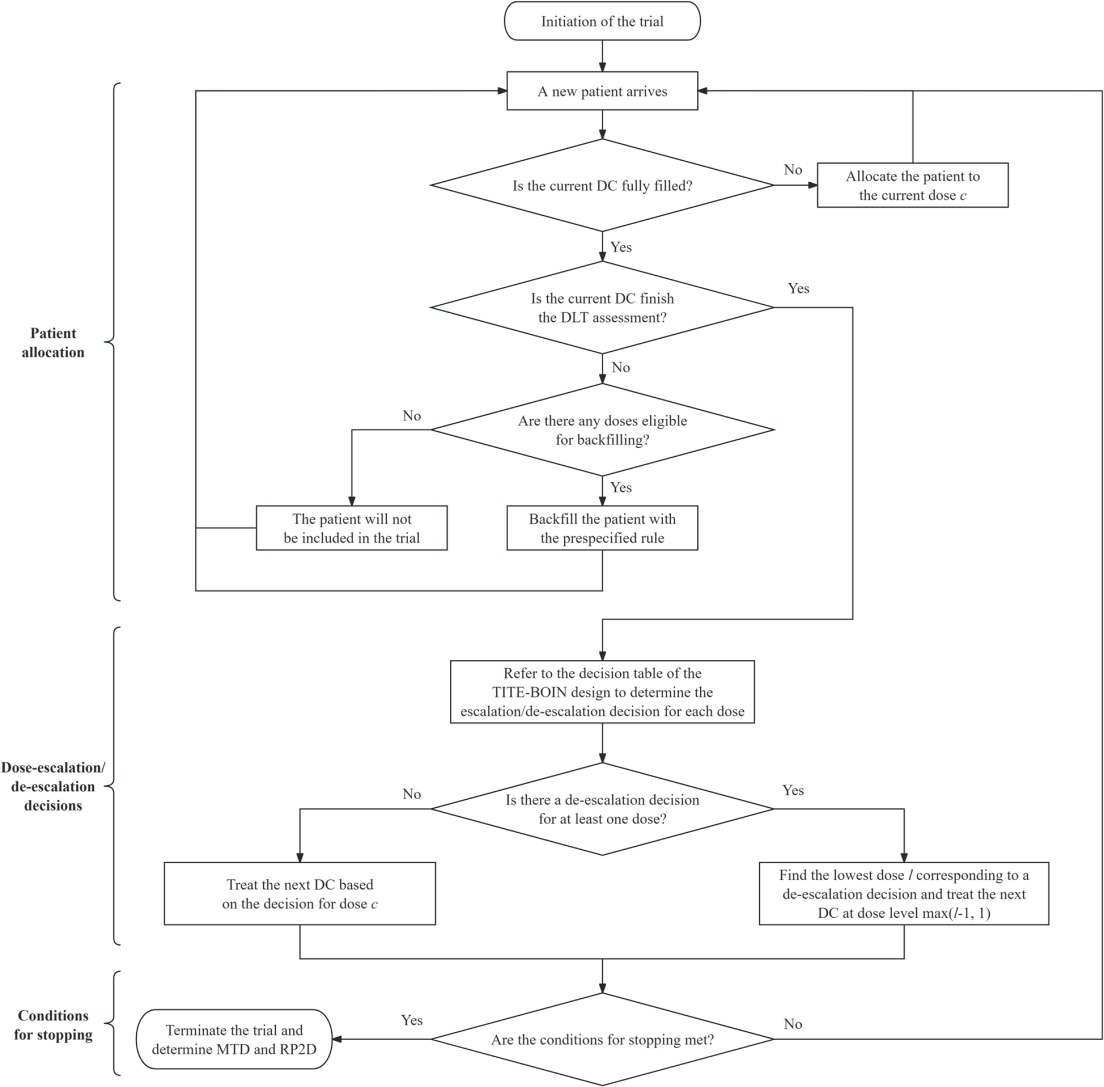
The flowchart of the Backfill-QoL design, where DC means the dose-escalation cohort

The dose-finding algorithm of the Backfill-QoL design can be summarized from three aspects: patient allocation, dose-escalation/de-escalation decisions, and conditions for stopping the trial (Fig. 1).

Two major issues need to be addressed in the context of dose-escalation designs with backfill cohorts. One is that there may be some pending DLT outcomes for backfill cohorts when patients in the current dose-escalation cohort complete DLT assessment. Therefore, we adopt the decision rules of the TITE-BOIN design [38], a version of the well-known Bayesian optimal interval (BOIN) design that deals with pending DLT outcomes, to facilitate the dose-finding in the Backfill-QoL design. Specifically, let ${x}_{ji}$ denote the binary DLT outcome for the ${i}_{th}$ patient treated with dose level $j$, where ${x}_{ji}=1$ indicating DLT and ${x}_{ji}=0$ indicating no DLT. When there are no pending DLT outcomes, the estimate of DLT probability ${\widehat{p}}_{j}={x}_{j}/{n}_{j}={\sum }_{i}{x}_{ji}/{n}_{j}$, and the BOIN design informs dose escalation/de-escalation decisions by comparing ${\widehat{p}}_{j}$ with two predetermined boundaries, ${\lambda }_{e}$ and ${\lambda }_{d}$. ${\widehat{p}}_{j}\le {\lambda }_{e}$, ${\widehat{p}}_{j}>{\lambda }_{d}$, and ${\lambda }_{e}<{\widehat{p}}_{j}\le {\lambda }_{d}$ correspond to dose escalation, dose de-escalation, and dose retention decisions, respectively. In the presence of pending outcomes, the estimate of DLT probability ${\sum }_{i}{x}_{ji}/{n}_{j}$ consists of two parts: ${x}_{ji}$ for observed outcomes and expected value of ${x}_{ji}$ for unobserved outcomes. Under the TITE-BOIN design, the unobserved outcomes are imputated by assuming the time to toxicity follows a uniform distribution over the assessment window $T$. Let ${T}_{ji}$ denote the time to toxicity for the ${i}_{th}$ patient treated with dose level $j$, the expected value of a missing ${x}_{ji}$ for a patient with follow-up time ${t}_{ji}$ is then given by $E({x}_{ji}|{T}_{ji}>{t}_{ji})$, which can be further approximated as ${p}_{j}(1-{t}_{ji}/T)/(1-{p}_{j})$ and the unknown ${p}_{j}$ can be estimated based on the observed data, which is elaborated in the appendix of Yuan et al. (2018) [38]. After the imputation, ${\widehat{p}}_{j}$ will be compared to ${\lambda }_{e}$ and ${\lambda }_{d}$, and the dose escalation/de-escalation decision can be derived for each dose. Due to safety concerns, it is not recommended to skip untried doses for dose-escalation cohorts.

Another issue is how to allocate patients for backfill cohorts. First, doses eligible for backfilling need to be updated with the monitoring rules. To avoid assigning too many patients to a specific low dose level, a sample size constraint can also be set [18]. Specifically, if the total number of patients treated with a dose level reaches ${n}_{cap}$, this dose will not be eligible for backfilling. For safety reasons, doses where the number of pending patients is more than half the total are also not eligible for backfilling. Then, the newly enrolled patient will be backfilled to one of eligible doses with a prespecified rule. For example, Dehbi et al. proposed to randomize backfill patients to the eligible doses with equal probability [17, 39], as the non-cytotoxic agents can present a plateau on the dose-efficacy curve and it is recommended to compare doses in a randomized fashion. Zhao et al. proposed to allocate patients to the highest eligible dose for backfilling [18], assuming the higher dose level is more likely to exhibit greater efficacy. Therefore, it is crucial to select an appropriate patient allocation method for backfill cohorts based on the understandings of the drug properties.

Let ${N}_{esc}$ denote the sample size for dose-escalation cohorts and the conditions for stopping the trial includes: (1) The sample size of dose-escalation cohorts reaches ${N}_{esc}$. (2) The number of patients treated at the current dose level $c$ reaches ${n}_{stop}$ and the decision is still to retain the dose. (3) The lowest dose level is deemed as overly toxic. (4) Other conditions requiring the trial to be stopped, e.g., an unexpected event of death occurs. Then, the MTD and RP2D are identified based on all collected safety, efficacy, and QoL data.

#### MTD and RP2D selection

At study conclusion, the MTD is identified based on an isotonic regression of all observed DLT outcomes. The dose with an estimated DLT probability that is closest to the target DLT rate is selected as the MTD. To select the RP2D, a comprehensive evaluation for safety, efficacy and QoL outcomes is required. Here we give the following rules for selecting the RP2D:

1. The RP2D is not higher than the MTD.
2. The RP2D must meet the prespecified criteria for efficacy and QoL, that is, $\text{Pr}\left({\theta }_{j}>{\theta }_{0}|Data\right)>{\varphi }_{E2}$ and $\text{Pr}\left({\widetilde{y}}_{j}<{\phi }_{QoL}|Data\right)<{\varphi }_{Q2}$. The criteria can be the same as or more stringent than those for trial monitoring.
3. Let $P$ denote the set of promising doses that satisfy the above two rules, and the dose with the best QoL performance in $P$ is denoted as ${d}_{Q}$. Then, according to the rank of estimated response rates of doses in $P$ from highest to lowest, the QoL performance of each dose is compared with ${d}_{Q}$ until one dose with ignorable difference in QoL with ${d}_{Q}$ is found. That dose is selected as the RP2D. The difference in QoL performance between dose $j$ and ${d}_{Q}$ is measured by a predictive probability, which is similar to the monitoring method for QoL described before. Specifically, if $\text{Pr}\left({\widetilde{y}}_{j}<{\widetilde{y}}_{{d}_{Q}}|Data\right)<{\varphi }_{C}$, the difference between dose $j$ and ${d}_{Q}$ in QoL is ignorable, where ${\varphi }_{C}$ is a prespecified cutoff.

It is worth noting that the third rule for RP2D selection can be modified based on specific clinical scenarios, e.g., selecting the RP2D from dose set $P$ solely based on the estimated response rate if the priority of tumor response is much higher than QoL. In addition, clinical pharmacokinetic, pharmacodynamic, and pharmacogenomic data may also be taken into account when selecting doses. Thus the research team should be given the discretion to choose the RP2D. When the sample size is insufficient to identify the optimal dose, a recommended dose range, rather than a single RP2D, can be given for subsequent randomized phase II trials [19].

### Software

The software for implementing the Backfill-QoL design was developed with R Shiny [40] and deployed as a Windows desktop application, which can be downloaded from https://github.com/cccc633/Backfill-QoL. This app includes the following modules: calibrating cutoffs for trial monitoring, providing the dose level that a newly enrolled patient should be allocated, selecting the MTD and RP2D at study conclusion, and running simulated trials. See section B of the Additional file 1 for a detailed description of the software.

### Simulation study

The operating characteristics of the proposed Backfill-QoL design are evaluated through a simulation study. A total of six candidate dose levels are investigated. The target DLT rate ${\phi }_{DLT}$ is 0.25, and the lowest acceptable response rate ${\theta }_{0}$ is 0.2. The maximum acceptable mean QoL deterioration of the FACT-G score ${\phi }_{QoL}$ is -10, the same as the setting of a multicenter randomized trial [41]. The accrual rate is 3 per 4 weeks and the time between two consecutive recruits is an independently and identically distributed exponential random variable with mean 4/3 weeks. The assessment windows of toxicity and efficacy are 4 weeks and 8 weeks, respectively. To assist dose-finding in a timely manner, the assessment window of QoL is set the same as that of toxicity. The maximum sample size for dose-escalation cohorts ${N}_{esc}$ is 36, and the cohort size is 3. The trial would be terminated when the number of patients treated at the current dose level reaches 9 (${n}_{stop}$) and the decision is still to retain the dose. If the number of patients treated with a dose level reaches 12 (${n}_{cap}$), this dose is not eligible for backfilling. As for patient allocation for backfill cohorts, equal randomization is adopted. The statistical cutoffs are set as follows: ${\varphi }_{T}=0.95$, ${\varphi }_{Q}=0.5$, ${\varphi }_{E}$=0.5, and ${\varphi }_{C}=0.75$. The values of ${\varphi }_{Q2}$ and ${\varphi }_{E2}$ are set the same as those of ${\varphi }_{Q}$ and ${\varphi }_{E}$ in the simulation study.

We compare the Backfill-QoL design with two alternative designs. The first is the simplified version of the proposed design that does not incorporate QoL outcomes (denoted as ‘Backfill’). The other for comparison consists of a dose-escalation phase using the BOIN design, followed by a dose-expansion phase where patients are randomized to one of the promising doses with equal probability (denoted as ‘DE-QoL’). Promising doses are not higher than the MTD and must meet the prespecified criteria for efficacy and QoL. The sample size for dose-expansion cohorts is set as ‘6 times the number of promising doses’ so that the overall sample size is similar to the Backfill-QoL. Trial settings such as the sample size and trial monitoring of the two designs for comparison are the same as those of the proposed design.

**Fig. 2:**
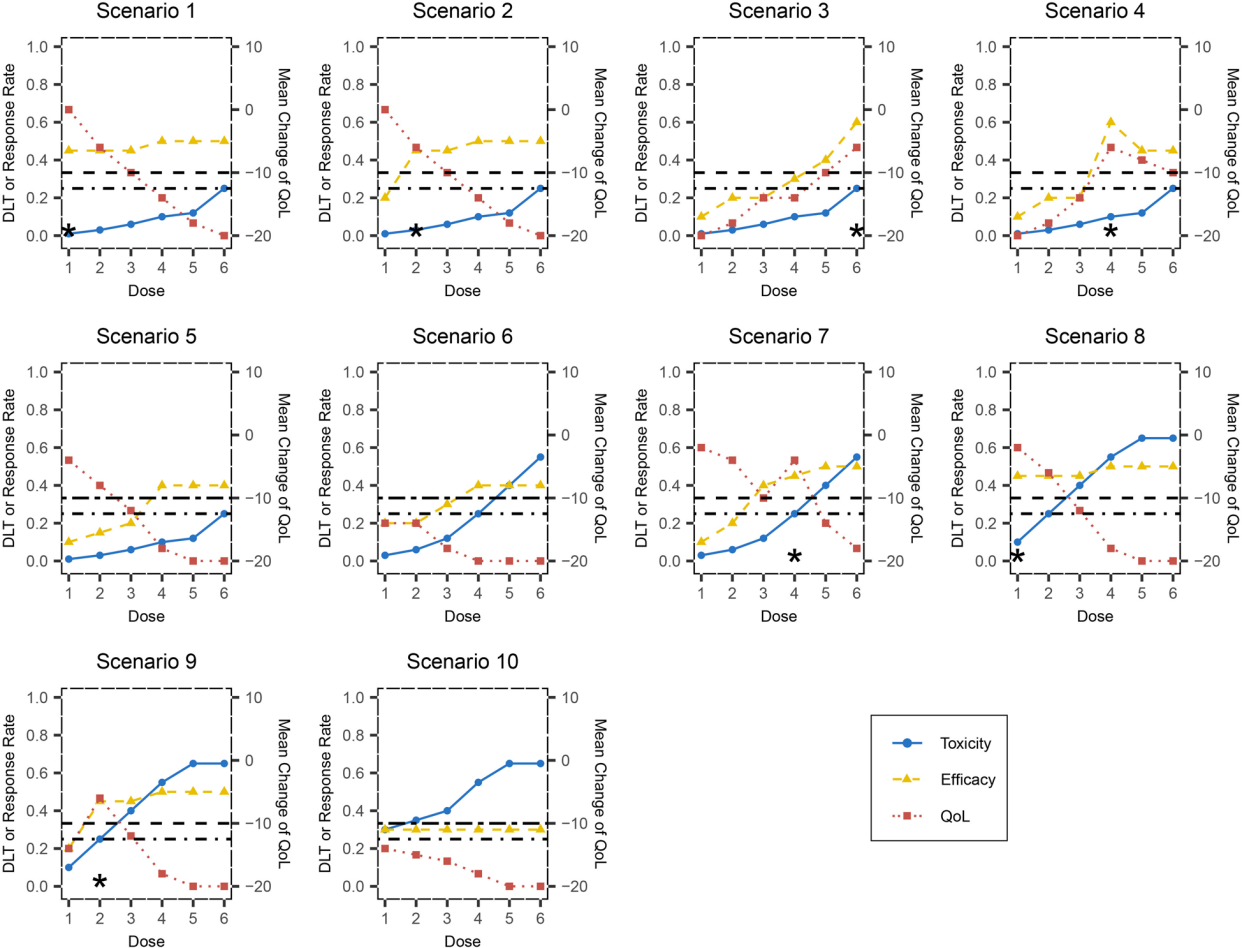
Ten scenarios evaluated in the simulation study. The dose-toxicity, dose–response and dose-QoL curves are shown by blue solid, yellow dashed and red dotted lines, respectively. The horizontal dashed and dash-dotted lines represent the maximum acceptable mean QoL deterioration of -10 and the DLT rate of 0.25. The asterisk indicates the optimal dose in each scenario

Ten scenarios with different dose-toxicity, dose–response, and dose-QoL curves are considered in the simulation study (Fig. 2). Specifically, the binary toxicity and efficacy outcomes for patients treated with dose level $j$ are simulated by Bernoulli distributions $Ber({p}_{j})$ and $Ber({\theta }_{j})$, respectively. The times to toxicity for those experiencing DLT events are simulated by a uniform distribution over $\left(0,T\right)$ and then rounded up to the next higher integer. The QoL outcome ${y}_{ij}$ is simulated by $N({\mu }_{j},{\sigma }_{j}^{2})$ and rounded to the nearest integer. Among these scenarios, the MTD is located at the highest dose level for scenarios 1 to 5, the 4th dose level for scenarios 6 and 7, the 2nd dose level for scenarios 8 and 9, and the 1st dose level for scenario 10. In scenarios 1 and 2, the dose–response curve plateaus at a low dose level, while the mean change of QoL decreases monotonically. In scenarios 3 and 4, the trend of the dose-QoL curve is consistent with that of the dose–response curve. In scenarios 5 and 6, no doses should be recommended as doses with high response rates lead to a QoL drop of nearly 20 points. In scenario 7, QoL first decreases with the increase of toxicity, then increases due to the improved efficacy, and finally decreases by excessive toxicity. In scenarios 8 and 9, only the first two doses have acceptable DLT rates, and the optimal dose is located at dose levels 1 and 2, respectively. In scenario 10, all candidate doses have DLT rate higher than ${\phi }_{DLT}$, as well as unacceptable QoL profiles. We also conduct several sensitivity analyses to assess the robustness of the proposed design and how design performance changes with the design parameters.

Metrics for evaluating operating characteristics include (1) the percentage of correctly selecting MTD (PCS-MTD); (2) the percentage of correctly selecting the optimal dose as RP2D (PCS-RP2D); (3) the expected sample size (EN); (4) the average trial duration. If no doses should be identified as RP2D (scenarios 5, 6, and 10), the PCS-RP2D is defined as the percentage of not selecting any dose as RP2D. All results are based on 1,000 simulated trials.

## Results

### Operating characteristics of the Backfill-QoL design

**Fig. 3:**
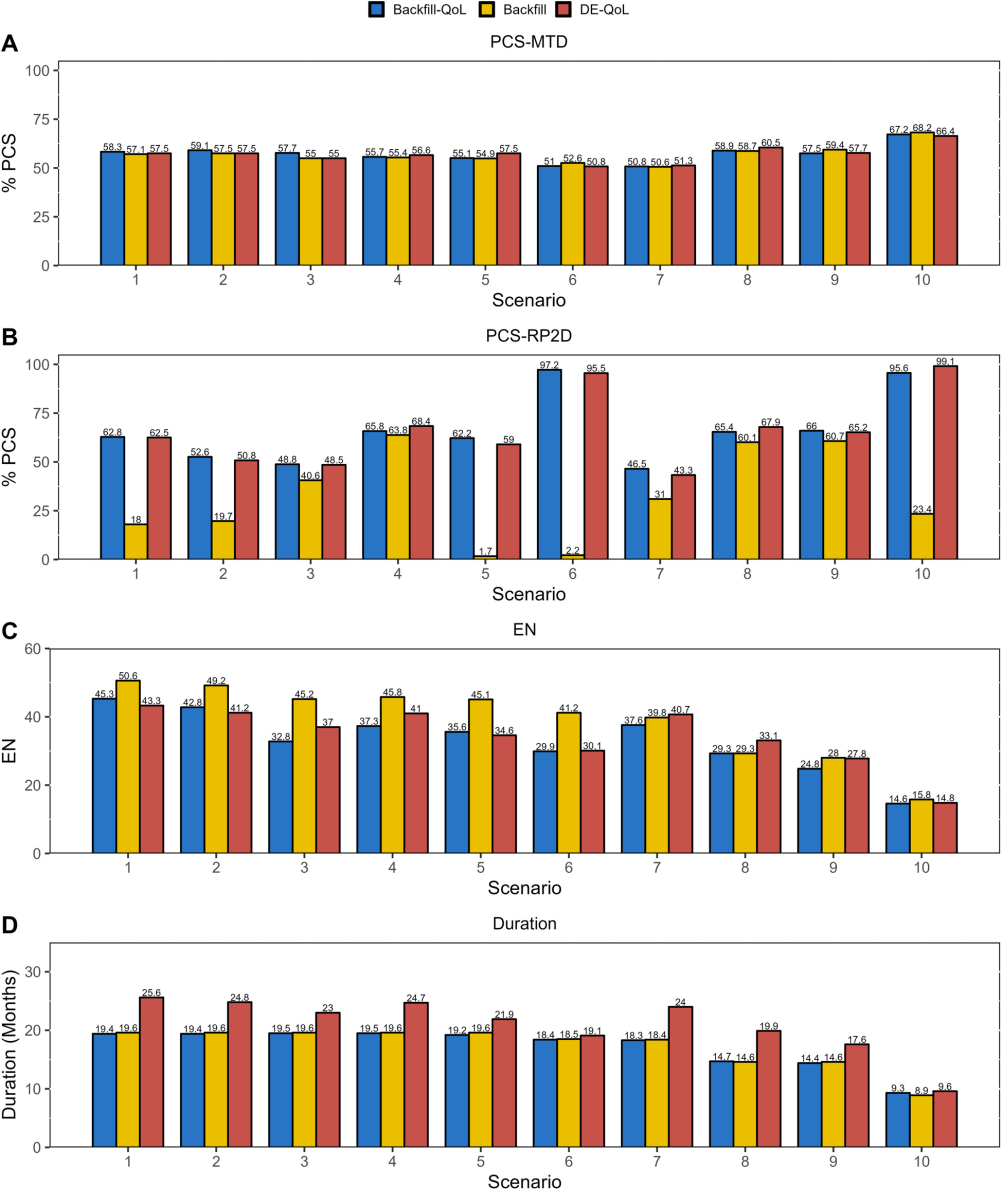
Comparison of three designs in ten scenarios on the percentage of correct selection, the expected sample size and the average trial duration

The proposed Backfill-QoL design selects MTD with almost the same accuracy as the Backfill and DE-QoL design (Fig. 3A), as their dose-escalation parts are all primarily directed by the BOIN design. We also investigate the operating characteristics of the BOIN design with the same simulation settings. It yields 59.0%, 49.9%, 59.2%, and 69.4% PCS-MTD for scenarios 1–5, 6–7, 8–9, and 10, suggesting that backfilling patients to low doses is of little use if there is a strong belief that the MTD is the optimal. In terms of the PCS-RP2D, the proposed Backfill-QoL and the DE-QoL are comparable on the whole, and both of them outperform the Backfill design where patient-reported QoL is not incorporated (Fig. 3B). When there exists doses with high response rates but unacceptable QoL profiles (scenarios 1, 2, 5 and 6), the Backfill design can hardly identify the true optimal dose. In scenario 10, the true DLT rate of the starting dose is 30% and there is a high probability that this dose is identified as the MTD. Compared with the Backfill design, the probability of the starting dose being identified as RP2D is much lower when applying Backfill-QoL or DE-QoL, due to the inclusion of QoL monitoring.

The expected sample size of the Backfill design is larger than that of the Backfill-QoL (Fig. 3C), as the former cannot eliminate doses with unacceptable QoL profiles and still backfill patients to those doses. This difference in EN is especially prominent when low dose levels have unacceptable QoL profiles, e.g., scenarios 3 and 6. The EN of the Backfill-QoL design is comparable with that of the DE-QoL design in most scenarios, but there are also minor differences (Fig. 3C). Specifically, when the MTD is at a high dose level and low doses are promising (scenarios 1 and 2), the Backfill-QoL would allocate more patients to low doses. For scenarios 3 and 4 where the DE-QoL yields larger EN, the number of patients allocated to low doses during the dose-escalation phase of DE-QoL is limited, causing doses that does not perform well on efficacy or QoL cannot be eliminated, and these doses would continue to be investigated in the dose-expansion phase. When the MTD is located at a low dose level (scenarios 8–10), the duration of dose-escalation is relatively short and the number of backfill patients could be limited.

Compared with DE-QoL, the Backfill-QoL and Backfill designs both shorten the average trial durations dramatically (Fig. 3D) because the backfilling component is synchronized with the dose-escalation process, unlike the dose-expansion that usually requires the dose-escalation phase to be completed. In general, dose-escalation trials with backfill cohorts would not raise additional safety concerns as patients can only be backfilled to doses that have been tested and regarded as safe, which is also supported by the simulation results. Detailed results of dose selection and patient allocation are shown in section C of the Additional file 1.

### Sensitivity analysis

Additional sensitivity analyses are conducted to assess the robustness of the proposed design (See section D of the Additional file 1 for detailed results). First, as the incorporation of QoL information is a key design feature of our proposed method, we investigate using QoL outcome-based adaptive randomization and the ‘pick-the-winner’ method to allocate doses for backfill patients. We compare equal randomization, adaptive randomization, pick-the-winner, and allocating patients to the highest eligible dose for backfilling (Sensitivity analysis 1). They yield almost identical performance on dose selection, expected sample size, and average trial duration. Therefore, in most cases, it is recommended to use an easy-to-implement method for backfilling patients, e.g., equal randomization or allocating patients to the highest eligible dose.

In terms of making dose escalation/de-escalation decisions, we investigate a more conservative method (Sensitivity analysis 2). That is, if there is a retention or de-escalation decision for at least one dose, find the lowest dose $l$ corresponding to a retention or de-escalation decision and treat the next dose-escalation cohort according to that decision. The sensitivity analysis shows this method yields similar PCS-RP2D and slightly lower PCS-MTD compared to the one we proposed earlier. The average number of patients treated above MTD is lower when using this more conservative method, but the decrease is extremely limited as it is rare to generate a retention decision on a dose while seeing a retention or even escalation decision for higher doses at the same time.

We also investigated the effects of accrual rate on design performance (Sensitivity analysis 3). A higher accrual rate means a larger sample size, especially when the MTD is located at a high dose level. But the increase in EN is usually not more than 5 when the accrual rate doubles from 3 to 6 per 4 weeks. This is mainly due to the setting of ${n}_{cap}$ and the rule that backfilling patients to doses where the number of pending patients is more than half the total is not allowed. In addition, if the accrual rate is very low, the number of patients to be backfilled may be too small to meet the requirements for dose optimization. Then it may be necessary to attach a dose-expansion component after the dose-escalation trial.

The statistical cutoffs described earlier also need to be carefully calibrated at the planning stage of a trial. Obviously, too stringent a cutoff may inadvertently result in excluding promising doses, while a cutoff that is too lenient has less power to rule out unacceptable doses. We show how design performance changes with statistical cutoffs ${\varphi }_{E}$, ${\varphi }_{Q}$, and ${\varphi }_{C}$ (Sensitivity analysis 4).

The performance of the proposed design is further examined under different correlations between toxicity, efficacy and QoL outcomes (Sensitivity analysis 5). Trivariate normal distributions are used to simultaneously simulate QoL outcomes and two latent variables for toxicity and efficacy, where a latent variable larger than 0 indicates the event of interest (DLT or response) occurs. The simulation results show that the correlations between these three outcomes have minor impacts on the operating characteristics if the marginal distribution for each outcome is fixed, consistent with findings of several optimal dose-finding designs [10, 42, 43].

Sample size determination is an issue that cannot be overlooked in any trial, so an additional analysis (Sensitivity analysis 6) is conducted to evaluate the impacts of sample size on the proposed design. In most scenarios, the larger the sample size, the higher the PCS, especially when the MTD and OBD locate at a high dose level. But there is often no significant improvement in design performance after the sample size has reached a certain level, mainly due to the sequential nature of dose escalation/de-escalation decisions. Therefore, it is critical to strike a tradeoff between the sample size and statistical performance by simulation studies at the planning stage. Under our proposed design, it is generally recommended to first design an MTD-finding trial as a reference, e.g., using a BOIN design that many investigators are relatively familiar with. The maximum sample size for dose-escalation cohorts can be conventionally set as six times the number of dose levels, and ${n}_{stop}$ is between 9 and 15. Based on such a MTD-finding design, the backfilling part can be further investigated. As shown in Sensitivity analysis 3, the accrual rate may be the most key factor for the sample size of backfilling cohorts. If there is a high accrual rate, it is necessary to carefully set ${n}_{cap}$ to constrain the sample size of backfilling cohorts. When the accrual rate is low, it may be necessary to attach a dose-expansion component after the dose-escalation trial to facilitate dose optimization.

## Discussion

In recent years, the backfill strategy has received a great deal of attention in oncology dose-escalation trials. Considering the potentially long efficacy assessment window, which may result in more patients being backfilled to ineffective doses, we propose to determine doses for backfill cohorts based on patient-reported QoL data in this paper. The proposed Backfill-QoL design enables continuous monitoring of safety, efficacy and QoL outcomes, and the RP2D is selected from a more patient-centered perspective. The simulation studies demonstrate that the proposed design is more efficient than the traditional dose-expansion strategy, and fewer patients would be allocated to doses with unacceptable QoL profiles due to the incorporation of QoL. Determining statistical cutoffs is a key issue when applying the proposed design, requiring the cooperation of both statisticians and clinicians. The general principle is that the cutoffs should be determined according to the disease background and the properties of the investigational drug. Based on overdosing boundaries and futility boundaries, e.g., Table 1, it can be judged from a clinical perspective whether the selections of ${\varphi }_{T}$, ${\varphi }_{E}$, and ${\varphi }_{E2}$ is too stringent or too lenient. Similarly, the cutoffs regarding QoL (i.e., ${\varphi }_{Q}$ and ${\varphi }_{Q2}$) can be calibrated based on Table 2, where the objective is to find such cutoffs so that doses with poor QoL profiles have a high probability to be detected, and doses that perform well on QoL have a low probability to be stopped early. Cutoff ${\varphi }_{C}$ reflects the tradeoff between QoL and efficacy outcomes, and is not easy to specify. It is recommended to conduct sensitivity analyses to evaluate the effects of the cutoffs on overall operating characteristics, just like Sensitivity analysis 4. The decision table and simulation results for various scenarios should be provided in advance to help determine the cutoffs. The statistical cutoffs can also be set as functions of the actual sample size to achieve greater flexibility [44].

In addition, the design proposed in this paper may need to be tailored to meet the practical clinical backgrounds. For example, the QoL may not be measured by the FACT-G total score, and the assumption of normality for ${y}_{ij}$ may not be valid. It is recommended to examine the distribution of PROMs of interest through previous studies and published literature, and consider whether it can be described with a skewed distribution such as the log-normal distribution. Or we can simply use the median and quartile of the observed PRO outcomes for trial monitoring and decision-making. If the variable type for measuring QoL is no longer continuous, some minor changes (e.g., monitoring rules for QoL) should be made to the proposed design. We are also investigating how to leverage longitudinal PRO data collected by mobile devices, rather than a landmark result, to guide dose finding. It is also crucial to emphasize that the PRO instrument must be valid, reliable, and fit-for-purpose. Otherwise, it may not accurately capture real QoL changes and clinical benefits, potentially leading to incorrect dose selection.

## Conclusions

The proposed Backfill-QoL design enables continuous monitoring of safety, efficacy and QoL outcomes, and the recommended phase II dose (RP2D) can be identified in a more patient-centered perspective. It has the potential to be applied in future phase I clinical trials with backfill cohorts.

## Supplementary Information

Additional file 1: Supplemental material.

## Data Availability

The data involved in this paper were obtained by simulation, and software that supports simulation can be downloaded from https://github.com/cccc633/Backfill-QoL.

## Abbreviations

BOIN: Bayesian optimal interval
DE: Dose-expansion
DLT: Dose-limiting toxicity
EN: Expected sample size
FACT-G: Functional Assessment of Cancer Therapy-General
MTD: Maximum tolerated dose
PCS: Percentage of correct selection
PRO: Patient-reported outcome
PROMs: Patient-reported outcome measures
PRO-CTCAE: Patient-reported outcomes version of the Common Terminology Criteria for Adverse Events
QoL: Quality of life
RP2D: Recommended phase II dose
