# Supplementary material for: Determining doses for backfill cohorts based on patient-reported outcome
Source: BMC Med Res Methodol. 2024 Nov 8;24:270. doi: 10.1186/s12874-024-02398-w (PMC11546322; doi:10.1186/s12874-024-02398-w)
Supplement: Supplementary file 1 — Additional file 1: Supplemental material. [file 12874_2024_2398_MOESM1_ESM.pdf]

Supplemental Material for "Determining doses for backfill cohorts based on patient-reported outcome"

Xin Chen[1], Jingyi Zhang[1], Bosheng Li[1] and Fangrong Yan[1]

[1]Department of Biostatistics, China Pharmaceutical University, Nanjing, China

A. Proof of the theorem utilized in QoL monitoring

Theorem: If φ_Q, n_j and $\Pr(y_j < \phi_{QoL})$ are fixed, then the probability of $\Pr(\tilde{y}_j < \phi_{QoL} | Data) > \varphi_Q$ is constant regardless of the specific values of μ_j and σ_j^2.

Proof:

The posterior predictive distribution of $\tilde{y}_j$ is $t_{(n_j-1)}(\bar{y}_j, (1+1/n_j)^{1/2}s_j)$, thus $\Pr(\tilde{y}_j < \phi_{QoL} | Data) > \varphi_Q$ can be represented as $\frac{\phi_{QoL}-\bar{y}_j}{(1+1/n_j)^{1/2}s_j} > qt_{(n_j-1)}(\varphi_Q)$, where $qt_{(n_j-1)}(\cdot)$ denotes the quantile function of a standard *t* distribution with $n_j - 1$ degrees of freedom. Then, this inequation can be rewritten as

$$\frac{\phi_{QoL}-\bar{y}_j}{(1/n_j)^{1/2}s_j} > (n_j+1)^{1/2}qt_{(n_j-1)}(\varphi_Q).$$

The left hand side of the inequation can be rewritten further as

$$\frac{\phi_{QoL}-(\bar{y}_j-\mu_j)-\mu_j}{(1/n_j)^{1/2}s_j},$$

consisting of two parts: $A = \frac{\phi_{QoL}-\mu_j}{(1/n_j)^{1/2}s_j}$ and $B = \frac{\bar{y}_j-\mu_j}{(1/n_j)^{1/2}s_j}$.

As $\Pr(y_j < \phi_{QoL})$ is a constant and $y_j \sim N(\mu_j, \sigma_j^2)$, $\frac{\phi_{QoL}-\mu_j}{\sigma_j}$ is naturally a constant. Let $\omega = \frac{\phi_{QoL}-\mu_j}{\sigma_j}$, then $\mu_j = \phi_{QoL} - \omega\sigma_j$ and $A = \frac{\omega\sigma_j}{(1/n_j)^{1/2}s_j}$. Based on basic knowledge of the sampling distribution, $(n_j-1)s_j^2/\sigma_j^2$ follows a chi-square distribution $\chi(n_j-1)$ with n_j-1 degrees of freedom, and $\bar{y}_j$ follows a normal distribution $N(\mu_j, \sigma_j^2/n_j)$. Let $C = (n_j-1)s_j^2/\sigma_j^2$, then $A = \omega(\frac{n_j(n_j-1)}{C})^{1/2}$ and $B|C \sim N(0, \frac{n_j-1}{C})$. Therefore, given C, whose distribution depends only on n_j, A is a

constant and B follows a specific normal distribution independent of the values of μ_j and σ_j. So the probability that $A - B > (n_j + 1)^{1/2} q t_{(n_j-1)}(\varphi_Q)$ is independent of the value of μ_j and σ_j, and can be calculated as follows:

$$\int_{0}^{+\infty} F_{B|C}\left(\omega\left(\frac{n_j(n_j-1)}{C}\right)^{\frac{1}{2}} - (n_j+1)^{\frac{1}{2}} q t_{(n_j-1)}(\varphi_Q)\right) f(C)dC,$$

where $F_{B|C}$ indicates the distribution function of B given C, i.e, the distribution function of $N(0, \frac{n_j-1}{C})$; $f(C)$ is the probability density function of C, i.e., the density function of $\chi(n_j - 1)$.

B. Introduction of the software for implementing the Backfill-QoL design

The software for implementing the Backfill-QoL design was deployed as a Windows desktop application and the user interface includes six main modules as follows: cutoff calibration for monitoring toxicity and efficacy, cutoff calibration for monitoring QoL, trial setting, trial conduct, dose selection, and simulation. Below we will go through their functions one by one.

1. Cutoff calibration for monitoring toxicity and efficacy

The software can provide the overdosing boundary and the futility boundary once the target DLT rate ϕ_{DLT}, the lowest acceptable response rate θ_0, and the statistical cutoffs (φ_T and φ_E) are input (Figure S1). Nmax is the maximum number of patients that can be allocated at a single dose level, controlling the width of the output table. The users can then calibrate the cutoffs easily according to the background of the disease. Multiple forms of output, e.g., Excel and PDF, are allowed.

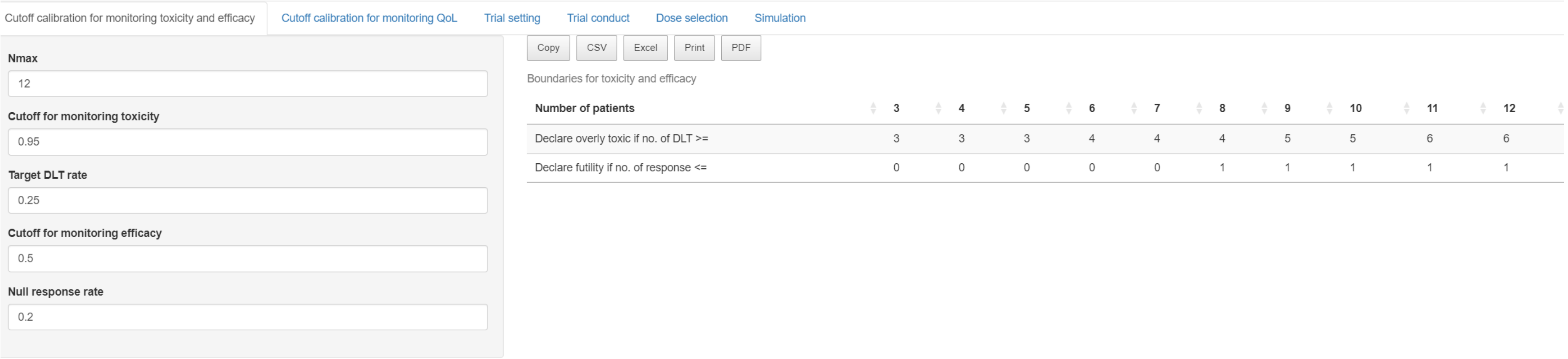

Figure S1 The software module that supports cutoff calibration for monitoring toxicity and efficacy.

2. Cutoff calibration for monitoring QoL

This module can provide the probabilities of declaring a dose with unacceptable QoL profiles when selecting different values of cutoff φ_Q under several scenarios, i.e., different sample sizes n_j and proportions of patient population with undesired QoL outcomes $\Pr(y_j < \phi_{QoL})$ (Figure S2). The users can then calibrate the cutoff by comparing those probabilities. The module now allows three values of both φ_Q and $\Pr(y_j < \phi_{QoL})$ to be input concurrently. Nmax is the maximum number of patients that can be allocated at a single dose level, and Nmin is the minimum sample size that can support eliminating a dose with an unacceptable QoL profile. If it is not necessary to calculate those probabilities for all sample sizes from Nmin to Nmax, we can set a Step larger than one to control the size of the output table.

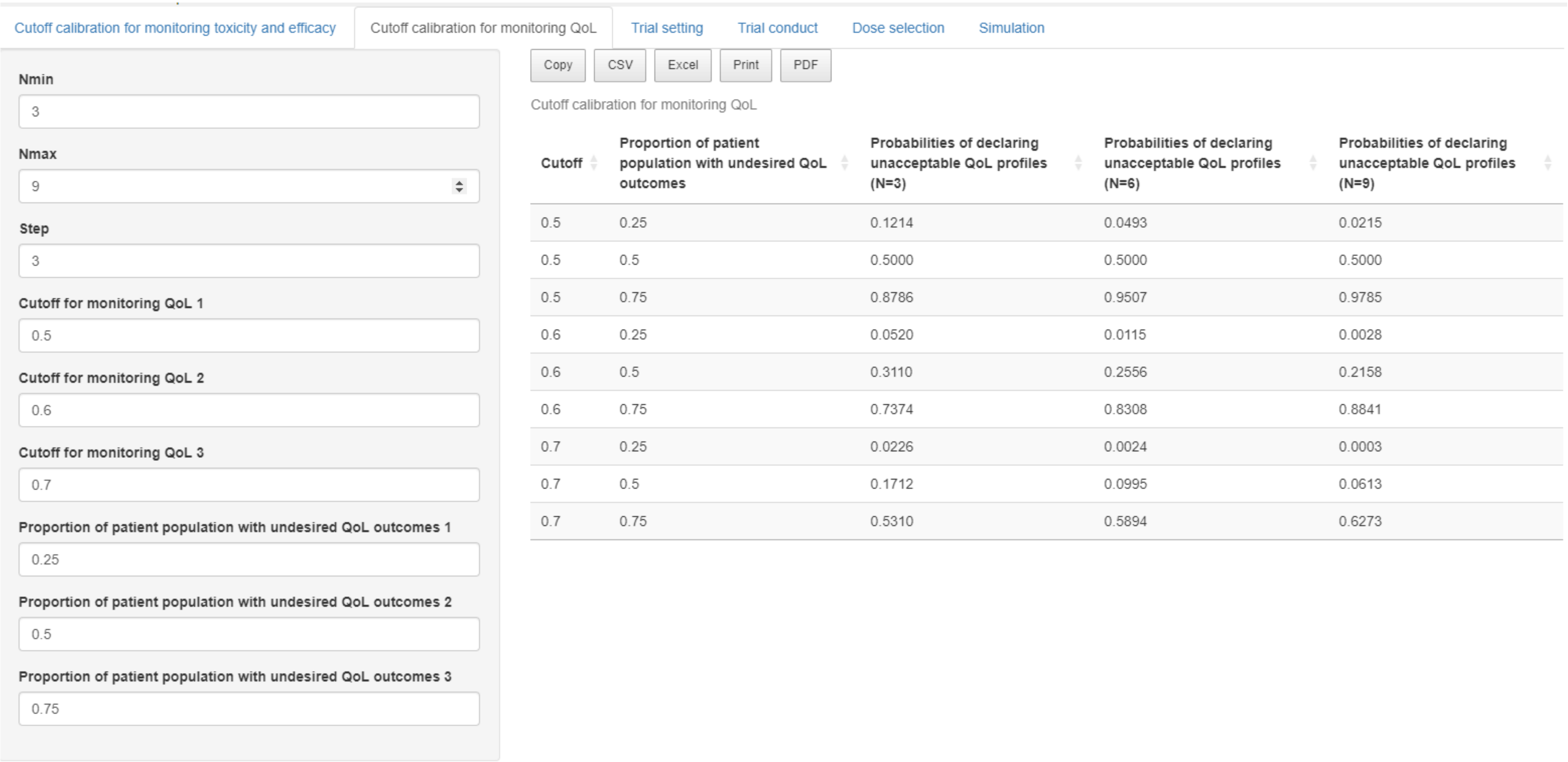

Cutoff	Proportion of patient population with undesired QoL outcomes	Probabilities of declaring unacceptable QoL profiles (N=3)	Probabilities of declaring unacceptable QoL profiles (N=6)	Probabilities of declaring unacceptable QoL profiles (N=9)
0.5	0.25	0.1214	0.0493	0.0215
0.5	0.5	0.5000	0.5000	0.5000
0.5	0.75	0.8786	0.9507	0.9785
0.6	0.25	0.0520	0.0115	0.0028
0.6	0.5	0.3110	0.2556	0.2158
0.6	0.75	0.7374	0.8308	0.8841
0.7	0.25	0.0226	0.0024	0.0003
0.7	0.5	0.1712	0.0995	0.0613
0.7	0.75	0.5310	0.5894	0.6273

Figure S2 The software module that supports cutoff calibration for monitoring QoL.

3. Trial setting

This module has no output and is used to input design parameters in preparation for the subsequent modules. The design parameters to be input here include N_esc (N_{esc}), n_cap (n_{cap}), n_stop (n_{stop}), the cohort size, the number of dose levels J, the target DLT rate ϕ_{DLT}, the null response rate θ_0, the maximum acceptable mean QoL deterioration ϕ_{DLT}, the assessment windows for DLT, QoL and efficacy, the cutoffs for monitoring (φ_T, φ_Q and φ_E), the cutoffs for QoL and efficacy at dose selection (φ_{Q2} and φ_{E2}), the cutoff for comparing QoL φ_C.

4. Trial conduct

This module can provide the dose level that a newly enrolled patient should be allocated based on available data. The users just need to upload the analysis dataset in the format of Excel and select the clinical cutoff date (here referring to the date when a new patient is enrolled), then the trial summary and the decision of dose allocation for the next patient will be output. The analysis dataset should contain six variables for each patient, including ‘Type’ (1 indicates dose-escalation cohorts while 2 indicates backfill cohorts), the allocated dose level, time of first administration, DLT outcome (1 indicates DLT while 0 indicates no DLT), response outcome (1 indicates response while 0 indicates no response), and QoL change from baseline.

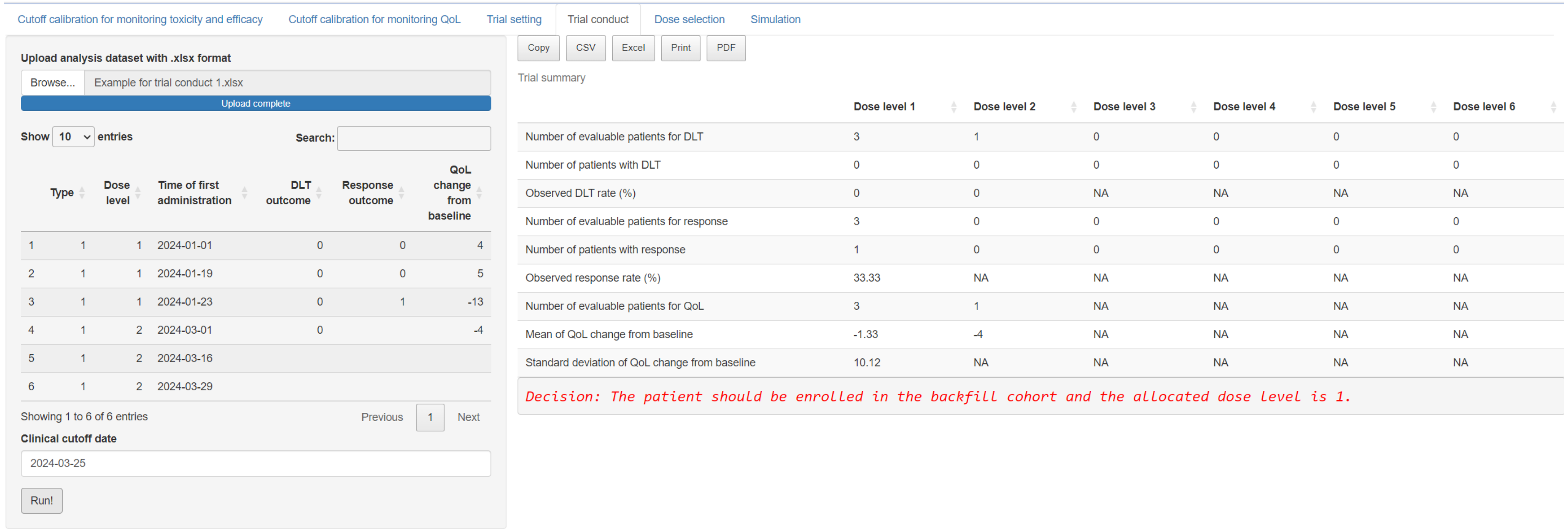

Figure S3 The software module that supports trial conduct.

5. Dose selection

The user interface of this module is very similar to that of the module for trial conduct. There is no need to give the clinical cutoff date due to no pending outcomes. In addition, the decision below the trial summary is the identified MTD and RP2D, instead of dose allocation.

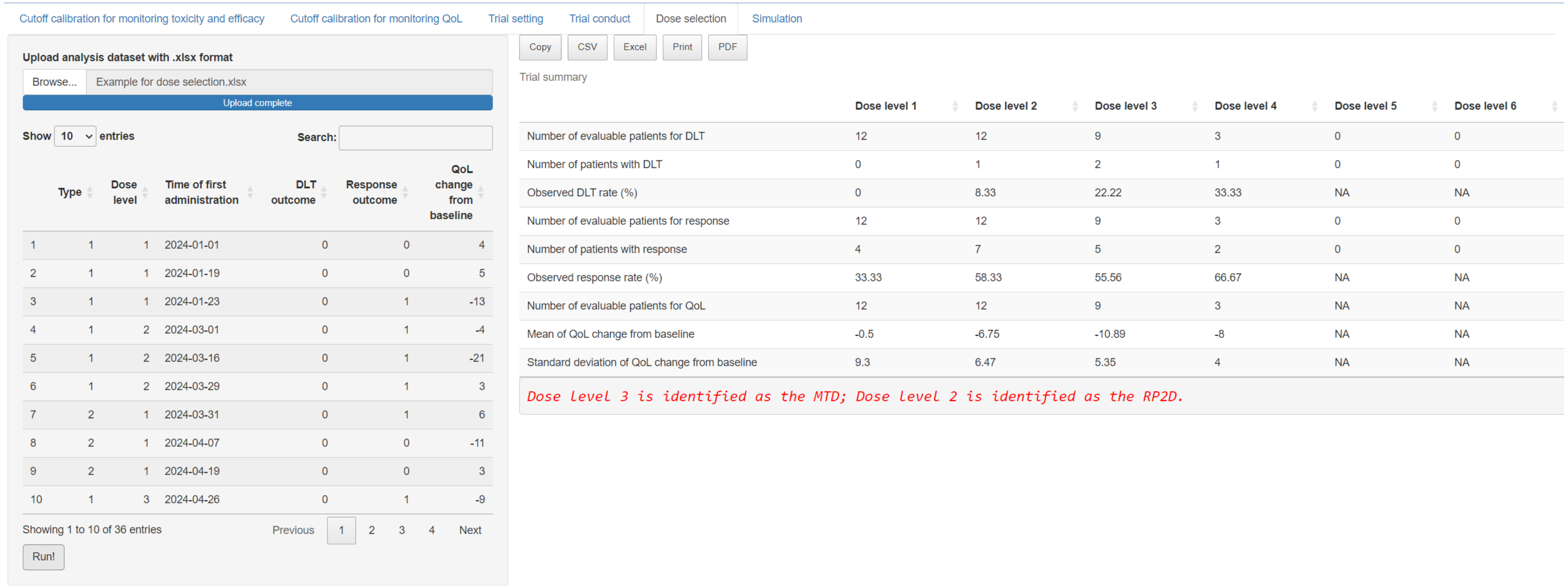

	Type	Dose level	Time of first administration	DLT outcome	Response outcome	QoL change from baseline
1	1	1	2024-01-01	0	0	4
2	1	1	2024-01-19	0	0	5
3	1	1	2024-01-23	0	1	-13
4	1	2	2024-03-01	0	1	-4
5	1	2	2024-03-16	0	1	-21
6	1	2	2024-03-29	0	1	3
7	2	1	2024-03-31	0	1	6
8	2	1	2024-04-07	0	0	-11
9	2	1	2024-04-19	0	0	3
10	1	3	2024-04-26	0	1	-9

	Dose level 1	Dose level 2	Dose level 3	Dose level 4	Dose level 5	Dose level 6
Number of evaluable patients for DLT	12	12	9	3	0	0
Number of patients with DLT	0	1	2	1	0	0
Observed DLT rate (%)	0	8.33	22.22	33.33	NA	NA
Number of evaluable patients for response	12	12	9	3	0	0
Number of patients with response	4	7	5	2	0	0
Observed response rate (%)	33.33	58.33	55.56	66.67	NA	NA
Number of evaluable patients for QoL	12	12	9	3	NA	NA
Mean of QoL change from baseline	-0.5	-6.75	-10.89	-8	NA	NA
Standard deviation of QoL change from baseline	9.3	6.47	5.35	4	NA	NA

Dose level 3 is identified as the MTD; Dose level 2 is identified as the RP2D.

Figure S4 The software module that supports dose selection.

6. Simulation

The function of this module is to run simulated trials for the Backfill-QoL design. The users are required to upload the simulation scenarios in the format of Excel and enter the number of scenarios, the standard deviation of QoL changes, the accrual rate, and the number of simulated trials for each scenario. The software supports parallel computing using CPU, so there is an option to fill in the number of available CPU cores. After clicking ‘run’ and waiting for some time (depending on the number of scenarios, the number of simulated trials, and the computing resource), the

software will produce the simulation results for each scenario, including the number and percentage of patients allocated to each dose level, the percentage of selecting each dose as the MTD, the percentage of selecting each dose as the RP2D, the expected sample size, and the average trial duration.

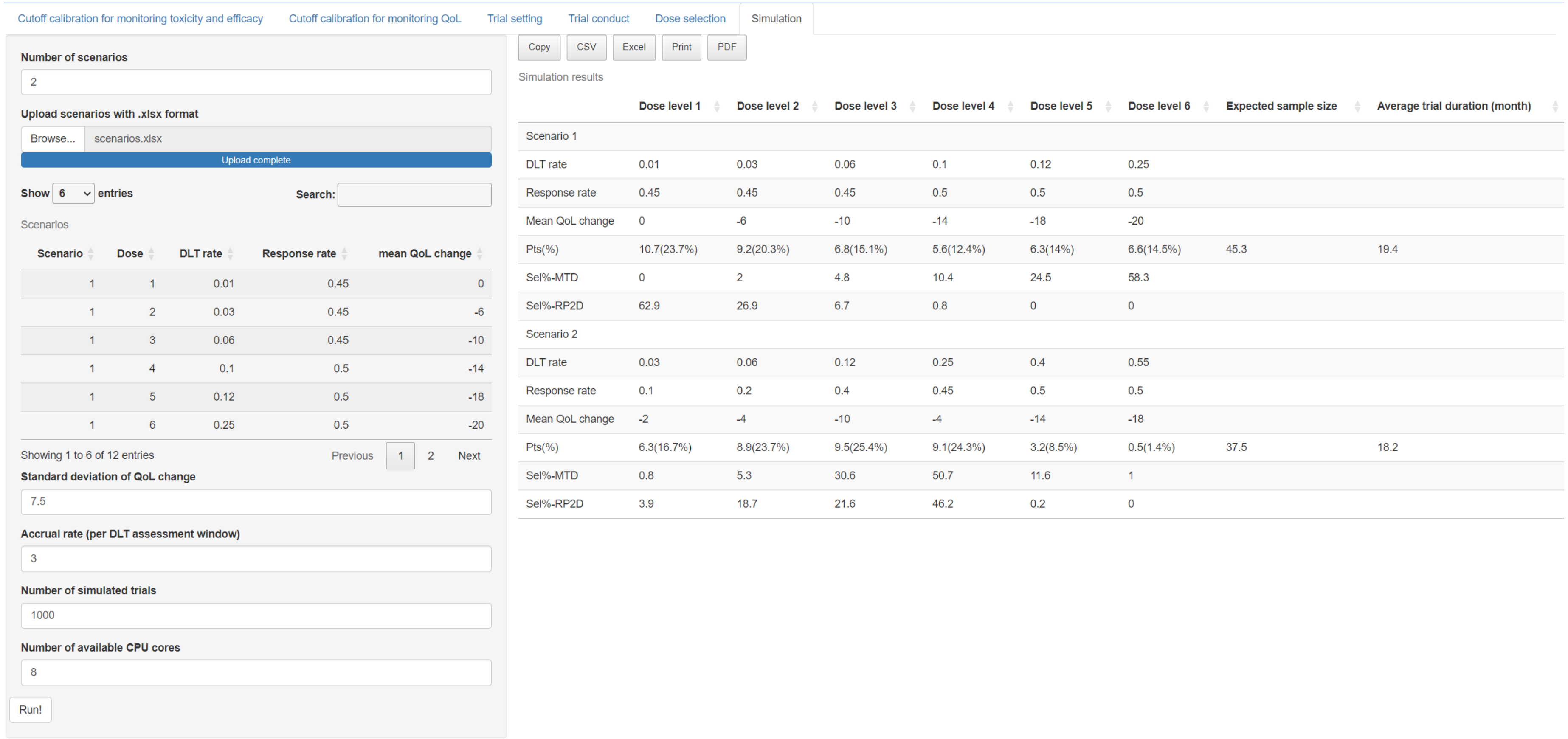

Figure S5 The software module that supports the simulation study.

C. Supplemental simulation results

Table S1. Detailed simulation results of three designs (Backfill-QoL, Backfill and DE-QoL) in ten scenarios.

Design		Dose Level						EN	Duration (Month)
		1	2	3	4	5	6		
Scenario 1	DLT rate	0.01	0.03	0.06	0.1	0.12	0.25		
	Response rate	0.45	0.45	0.45	0.5	0.5	0.5		
	Mean change of QoL	0	-6	-10	-14	-18	-20		
Backfill-QoL	Pts(%)	10.7(23.7%)	9.2(20.3%)	6.8(15.1%)	5.6(12.4%)	6.3(14%)	6.6(14.5%)	45.3	19.4
	Sel%-MTD	0	2	4.8	10.4	24.5	58.3		
	Sel%-RP2D	62.8	27	6.7	0.8	0	0		
Backfill	Pts(%)	10.2(20.1%)	9.3(18.4%)	8.9(17.6%)	8.2(16.3%)	7.6(14.9%)	6.4(12.6%)	50.6	19.6
	Sel%-MTD	0	1.9	5.2	10.4	25.4	57.1		
	Sel%-RP2D	18	16.2	15.1	22.7	17.7	10.3		
DE-QoL	Pts(%)	8.7(20.1%)	8.3(19.1%)	7.4(17.1%)	6(13.9%)	6.4(14.8%)	6.5(15.1%)	43.3	25.6
	Sel%-MTD	0	0.9	3.9	12	25.7	57.5		
	Sel%-RP2D	62.5	27.8	7	0.8	0	0		
Scenario 2	DLT rate	0.01	0.03	0.06	0.1	0.12	0.25		
	Response rate	0.2	0.45	0.45	0.5	0.5	0.5		
	Mean change of QoL	0	-6	-10	-14	-18	-20		
Backfill-QoL	Pts(%)	7.8(18.2%)	9.4(22%)	7.1(16.6%)	5.6(13.1%)	6.3(14.7%)	6.6(15.4%)	42.8	19.4
	Sel%-MTD	0	1.8	5.1	10.4	23.6	59.1		
	Sel%-RP2D	22	52.6	13.2	1.6	0	0.1		
Backfill	Pts(%)	7.4(15.1%)	9.8(19.9%)	9.3(19%)	8.4(17.1%)	7.7(15.7%)	6.5(13.2%)	49.2	19.6
	Sel%-MTD	0	1.8	5	10.5	25.2	57.5		
	Sel%-RP2D	1	19.7	19.8	28.8	19.8	10.9		

DE-QoL	Pts(%)	7.1(17.2%)	7.9(19.2%)	7.3(17.6%)	6(14.6%)	6.4(15.6%)	6.5(15.8%)	41.2	24.8
	Sel%-MTD	0	1	3.9	11.9	25.7	57.5		
	Sel%-RP2D	24.2	50.8	14.9	1.7	0	0		
Scenario 3	DLT rate	0.01	0.03	0.06	0.1	0.12	0.25		
	Response rate	0.1	0.2	0.2	0.3	0.4	0.6		
	Mean change of QoL	-20	-18	-14	-14	-10	-6		
Backfill-QoL	Pts(%)	3.4(10.2%)	4(12.1%)	5.2(15.8%)	5.9(17.9%)	8(24.2%)	6.5(19.8%)	32.8	19.5
	Sel%-MTD	0	1	4.3	11.9	25.1	57.7		
	Sel%-RP2D	0	0	0.3	1	18.3	48.8		
Backfill	Pts(%)	5.7(12.7%)	7.9(17.4%)	8.2(18.2%)	8.7(19.2%)	8.4(18.6%)	6.3(13.9%)	45.2	19.6
	Sel%-MTD	0	1.4	4.6	12.2	26.8	55		
	Sel%-RP2D	1.8	6.9	7.6	16.9	24.5	40.6		
DE-QoL	Pts(%)	3.8(10.4%)	4.4(12%)	5.6(15.1%)	6.2(16.8%)	8.4(22.6%)	8.6(23.1%)	37	23
	Sel%-MTD	0	1	4.2	11.8	28	55		
	Sel%-RP2D	0	0.1	1.2	2.1	16.6	48.5		
Scenario 4	DLT rate	0.01	0.03	0.06	0.1	0.12	0.25		
	Response rate	0.1	0.2	0.2	0.6	0.45	0.45		
	Mean change of QoL	-20	-18	-14	-6	-8	-10		
Backfill-QoL	Pts(%)	3.4(9%)	4(10.7%)	5.2(13.9%)	10.1(27.2%)	8.4(22.5%)	6.3(16.8%)	37.3	19.5
	Sel%-MTD	0	1	4.3	12.1	26.9	55.7		
	Sel%-RP2D	0	0	0.2	65.8	18	7.8		
Backfill	Pts(%)	5.7(12.5%)	7.8(17%)	8.2(17.8%)	9.7(21.2%)	8.2(17.9%)	6.2(13.6%)	45.8	19.6
	Sel%-MTD	0	1.4	4.6	12.2	26.4	55.4		
	Sel%-RP2D	0.7	3	3.5	63.8	17.4	10.6		
DE-QoL	Pts(%)	3.8(9.3%)	4.5(10.9%)	5.7(13.9%)	9.3(22.6%)	9.6(23.5%)	8.1(19.8%)	41	24.7
	Sel%-MTD	0	1	4.2	11.6	26.6	56.6		

	Sel%-RP2D	0	0	0.3	68.4	17.1	6		
Scenario 5	DLT rate	0.01	0.03	0.06	0.1	0.12	0.25		
	Response rate	0.1	0.15	0.2	0.4	0.4	0.4		
	Mean change of QoL	-4	-8	-12	-18	-20	-20		
Backfill-QoL	Pts(%)	5.7(16%)	6.3(17.7%)	5.7(15.9%)	5.4(15.2%)	6.2(17.5%)	6.3(17.8%)	35.6	19.2
	Sel%-MTD	0	1.4	4.2	12.3	27	55.1		
	Sel%-RP2D	14.1	18.5	5.2	0	0	0		
Backfill	Pts(%)	5.7(12.8%)	7.1(15.8%)	8.3(18.4%)	9.3(20.6%)	8.4(18.7%)	6.2(13.8%)	45.1	19.6
	Sel%-MTD	0	1.4	4.6	11.9	27.2	54.9		
	Sel%-RP2D	1.7	3.2	7.7	38.3	29.5	17.9		
DE-QoL	Pts(%)	5.6(16.1%)	5.3(15.4%)	5.4(15.5%)	5.4(15.5%)	6.4(18.5%)	6.5(18.9%)	34.6	21.9
	Sel%-MTD	0	1	4.2	11.8	25.5	57.5		
	Sel%-RP2D	15.2	19	6.6	0.2	0	0		
Scenario 6	DLT rate	0.03	0.06	0.12	0.25	0.4	0.55		
	Response rate	0.2	0.2	0.3	0.4	0.4	0.4		
	Mean change of QoL	-14	-14	-18	-20	-20	-20		
Backfill-QoL	Pts(%)	4.3(14.5%)	5.7(19%)	7.8(26%)	7.8(26.2%)	3.5(11.9%)	0.7(2.4%)	29.9	18.4
	Sel%-MTD	0.8	5.9	27.1	51	14.5	0.7		
	Sel%-RP2D	0.9	1.9	0	0	0	0		
Backfill	Pts(%)	8.2(19.9%)	9(21.8%)	11.1(27%)	9(21.9%)	3.4(8.2%)	0.5(1.3%)	41.2	18.5
	Sel%-MTD	0.9	4.9	30	52.6	11.1	0.5		
	Sel%-RP2D	11.8	11.9	33.8	36.1	4.1	0.1		
DE-QoL	Pts(%)	4.6(15.3%)	5.5(18.2%)	7.8(26%)	7.9(26.2%)	3.5(11.7%)	0.7(2.4%)	30.1	19.1
	Sel%-MTD	0.8	5.7	27.9	50.8	14	0.8		
	Sel%-RP2D	1.4	2.9	0.2	0	0	0		
Scenario 7	DLT rate	0.03	0.06	0.12	0.25	0.4	0.55		

	Response rate	0.1	0.2	0.4	0.45	0.5	0.5		
	Mean change of QoL	-2	-4	-10	-4	-14	-18		
Backfill-QoL	Pts(%)	6.3(16.7%)	8.9(23.7%)	9.5(25.4%)	9.2(24.4%)	3.2(8.5%)	0.5(1.4%)	37.6	18.3
	Sel%-MTD	0.8	5.3	30.4	50.8	11.7	1		
	Sel%-RP2D	3.9	18.7	21.6	46.5	0.2	0		
Backfill	Pts(%)	6.3(15.8%)	9(22.7%)	11.6(29.1%)	9(22.6%)	3.4(8.4%)	0.6(1.4%)	39.8	18.4
	Sel%-MTD	0.8	5.4	30.6	50.6	11.9	0.7		
	Sel%-RP2D	1.5	9.1	49.8	31	5.6	0.2		
DE-QoL	Pts(%)	6.1(15.1%)	8.6(21.1%)	10.5(25.8%)	11.1(27.4%)	3.6(8.8%)	0.7(1.8%)	40.7	24
	Sel%-MTD	0.8	5.3	29.6	51.3	12.2	0.8		
	Sel%-RP2D	6.2	17.5	22.4	43.3	0.1	0		
Scenario 8	DLT rate	0.1	0.25	0.4	0.55	0.65	0.65		
	Response rate	0.45	0.45	0.45	0.5	0.5	0.5		
	Mean change of QoL	-2	-6	-12	-18	-20	-20		
Backfill-QoL	Pts(%)	13.3(45.4%)	11.1(37.8%)	4(13.8%)	0.8(2.7%)	0.1(0.2%)	0(0%)	29.3	14.7
	Sel%-MTD	26.1	58.9	13.9	0.7	0	0		
	Sel%-RP2D	65.4	32.2	0.8	0	0	0		
Backfill	Pts(%)	13.3(45.4%)	11(37.7%)	4.1(14.1%)	0.8(2.6%)	0.1(0.2%)	0(0%)	29.3	14.6
	Sel%-MTD	26.5	58.7	13.9	0.5	0	0		
	Sel%-RP2D	60.1	33.9	4.8	0.1	0	0		
DE-QoL	Pts(%)	14.3(43.1%)	13.5(40.8%)	4.4(13.3%)	0.8(2.5%)	0.1(0.2%)	0(0%)	33.1	19.9
	Sel%-MTD	25.4	60.5	13.3	0.5	0	0		
	Sel%-RP2D	67.9	30.1	0.4	0	0	0		
Scenario 9	DLT rate	0.1	0.25	0.4	0.55	0.65	0.65		
	Response rate	0.2	0.45	0.45	0.5	0.5	0.5		
	Mean change of QoL	-14	-6	-12	-18	-20	-20		

Backfill-QoL	Pts(%)	8.9(35.9%)	11.1(44.8%)	3.9(15.9%)	0.8(3.2%)	0.1(0.2%)	0(0%)	24.8	14.4
	Sel%-MTD	27.4	57.5	14	0.8	0	0		
	Sel%-RP2D	1.2	66	1.4	0	0	0		
Backfill	Pts(%)	11.7(41.8%)	11.3(40.2%)	4.2(15%)	0.8(2.7%)	0.1(0.2%)	0(0%)	28	14.6
	Sel%-MTD	26.2	59.4	13.5	0.5	0	0		
	Sel%-RP2D	20.4	60.7	6.8	0.2	0	0		
DE-QoL	Pts(%)	10.7(38.5%)	11.8(42.5%)	4.4(15.8%)	0.8(3%)	0.1(0.2%)	0(0%)	27.8	17.6
	Sel%-MTD	27.9	57.7	13.3	0.8	0	0		
	Sel%-RP2D	0.5	65.2	0.6	0	0	0		
Scenario 10	DLT rate	0.3	0.35	0.4	0.55	0.65	0.65		
	Response rate	0.3	0.3	0.3	0.3	0.3	0.3		
	Mean change of QoL	-14	-15	-16	-18	-20	-20		
Backfill-QoL	Pts(%)	9.3(63.6%)	3.8(26.1%)	1.2(8.3%)	0.3(1.8%)	0(0.2%)	0(0%)	14.6	9.3
	Sel%-MTD	67.2	12.3	3.5	0.5	0	0		
	Sel%-RP2D	4.3	0	0.1	0	0	0		
Backfill	Pts(%)	10.6(66.7%)	3.9(24.8%)	1.2(7.3%)	0.2(1.1%)	0(0.1%)	0(0%)	15.8	8.9
	Sel%-MTD	68.2	12	2.3	0.2	0	0		
	Sel%-RP2D	68.4	7.5	0.7	0	0	0		
DE-QoL	Pts(%)	9.4(63.6%)	3.8(25.8%)	1.2(8.4%)	0.3(1.9%)	0(0.2%)	0(0%)	14.8	9.6
	Sel%-MTD	66.4	12.8	3.6	0.5	0	0		
	Sel%-RP2D	0.8	0.1	0	0	0	0		

Note: Pts(%), the average number and percentage of patients allocated to each dose; Sel%-MTD, the percentage of selecting each dose as the MTD; Sel%-RP2D, the percentage of selecting each dose as the RP2D; EN, the expected sample size.

D. Simulation results for the sensitivity analyses

Sensitivity analysis 1

Let D denote the set of eligible doses for backfilling patients. In terms of the QoL outcome-based adaptive randomization (AR), the probability that a patient is randomized to dose $j(j \in D)$ is proportional to the square root of the probability that the posterior predictive QoL outcome of dose j is the optimal among D. That is, the randomization probability $AR_j \propto \Pr(\tilde{y}_j > \tilde{y}_{j'} | Data, \{j, j'\} = D, j' \neq j)^{1/2}$. The main purpose of introducing square roots is to reduce the chances of overly extreme randomization probabilities. As for the pick-the-winner strategy, patients are backfilled to the dose that has the optimal posterior predictive QoL outcome among D, i.e., $\underset{j \in D}{\operatorname{argmax}} \left\{ \Pr(\tilde{y}_j > \tilde{y}_{j'} | Data, \{j, j'\} = D, j' \neq j) \right\}$.

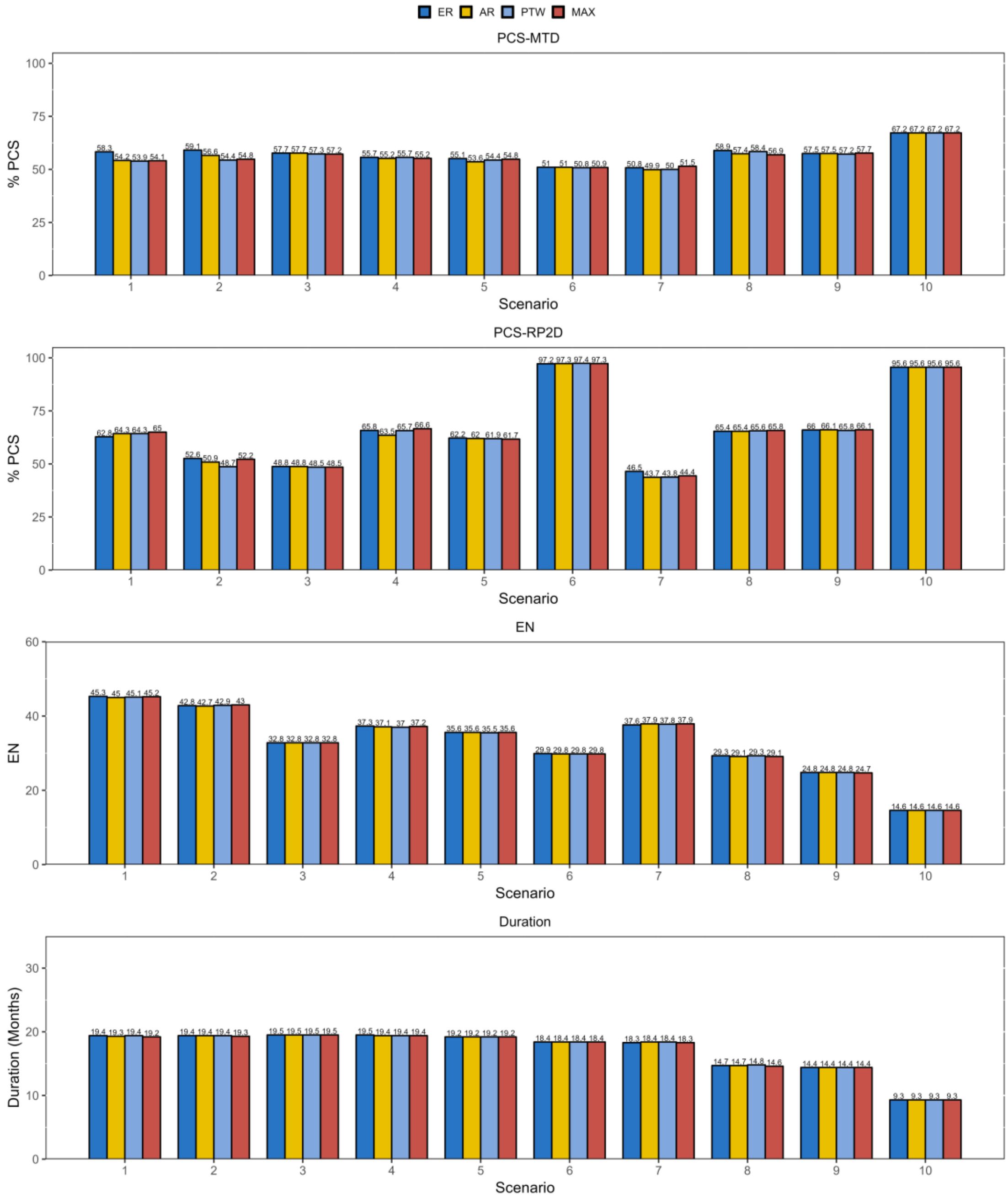

Figure S6 Comparison of four dose allocation methods for backfill cohorts on the percentage of correct selection, the expected sample size and the average trial duration. ER, AR, PTW and MAX indicate equal randomization, QoL outcome-based adaptive randomization, pick the winner, and backfilling patients to the highest eligible dose level, respectively.

Table S2. Detailed simulation results of sensitivity analysis 1.

Design		Dose Level						EN	Duration (Month)
		1	2	3	4	5	6		
Scenario 1	DLT rate	0.01	0.03	0.06	0.1	0.12	0.25		
	Response rate	0.45	0.45	0.45	0.5	0.5	0.5		
	Mean change of QoL	0	-6	-10	-14	-18	-20		
ER	Pts(%)	10.7(23.7%)	9.2(20.3%)	6.8(15.1%)	5.6(12.4%)	6.3(14%)	6.6(14.5%)	45.3	19.4
	Sel%-MTD	0	2	4.8	10.4	24.5	58.3		
	Sel%-RP2D	62.8	27	6.7	0.8	0	0		
AR	Pts(%)	10.8(24%)	9.1(20.2%)	6.9(15.4%)	5.8(12.9%)	6.2(13.8%)	6.2(13.8%)	45	19.3
	Sel%-MTD	0	1.9	5.4	12.5	26	54.2		
	Sel%-RP2D	64.3	26.1	6.4	0.5	0	0		
PTW	Pts(%)	10.9(24.2%)	9.1(20.1%)	6.8(15.2%)	5.6(12.5%)	6.4(14.1%)	6.3(14%)	45.1	19.4
	Sel%-MTD	0	1.9	5.4	11.5	27.3	53.9		
	Sel%-RP2D	64.3	25.3	7.2	0.7	0	0		
MAX	Pts(%)	10.1(22.3%)	9.5(20.9%)	7.4(16.4%)	5.8(12.7%)	6.2(13.8%)	6.2(13.8%)	45.2	19.2
	Sel%-MTD	0	2	5.7	12.4	25.8	54.1		
	Sel%-RP2D	65	26.1	5.7	0.3	0	0		
Scenario 2	DLT rate	0.01	0.03	0.06	0.1	0.12	0.25		
	Response rate	0.2	0.45	0.45	0.5	0.5	0.5		
	Mean change of QoL	0	-6	-10	-14	-18	-20		
ER	Pts(%)	7.8(18.2%)	9.4(22%)	7.1(16.6%)	5.6(13.1%)	6.3(14.7%)	6.6(15.4%)	42.8	19.4
	Sel%-MTD	0	1.8	5.1	10.4	23.6	59.1		
	Sel%-RP2D	22	52.6	13.2	1.6	0	0.1		
AR	Pts(%)	7.8(18.4%)	9.4(22%)	7.2(16.8%)	5.7(13.4%)	6.2(14.6%)	6.3(14.8%)	42.7	19.4
	Sel%-MTD	0	1.5	6	12.1	23.8	56.6		

	Sel%-RP2D	22.5	50.9	14.5	1.3	0	0		
PTW	Pts(%)	8(18.6%)	9.4(21.9%)	7.2(16.7%)	5.8(13.4%)	6.3(14.7%)	6.3(14.6%)	42.9	19.4
	Sel%-MTD	0	1.5	5.1	11.3	27.7	54.4		
	Sel%-RP2D	24.4	48.7	14.4	2.3	0	0		
MAX	Pts(%)	7.5(17.5%)	9.5(22.1%)	7.6(17.7%)	5.9(13.7%)	6.2(14.5%)	6.3(14.6%)	43	19.3
	Sel%-MTD	0	1.5	5.2	12.5	26	54.8		
	Sel%-RP2D	22.7	52.2	13	1.1	0	0		
Scenario 3	DLT rate	0.01	0.03	0.06	0.1	0.12	0.25		
	Response rate	0.1	0.2	0.2	0.3	0.4	0.6		
	Mean change of QoL	-20	-18	-14	-14	-10	-6		
ER	Pts(%)	3.4(10.2%)	4(12.1%)	5.2(15.8%)	5.9(17.9%)	8(24.2%)	6.5(19.8%)	32.8	19.5
	Sel%-MTD	0	1	4.3	11.9	25.1	57.7		
	Sel%-RP2D	0	0	0.3	1	18.3	48.8		
AR	Pts(%)	3.4(10.2%)	4(12.1%)	5.2(15.8%)	5.9(17.9%)	7.9(24.2%)	6.5(19.7%)	32.8	19.5
	Sel%-MTD	0	1	4.3	11.9	25.1	57.7		
	Sel%-RP2D	0	0	0.3	1.1	18.3	48.8		
PTW	Pts(%)	3.4(10.2%)	4(12.2%)	5.2(15.8%)	5.9(17.9%)	7.9(24.2%)	6.4(19.7%)	32.8	19.5
	Sel%-MTD	0	1	4.3	11.9	25.5	57.3		
	Sel%-RP2D	0	0	0.3	1	18.4	48.5		
MAX	Pts(%)	3.4(10.2%)	4(12.2%)	5.2(15.8%)	5.8(17.8%)	7.9(24.2%)	6.4(19.7%)	32.8	19.5
	Sel%-MTD	0	1	4.3	11.9	25.6	57.2		
	Sel%-RP2D	0	0	0.3	1	18.4	48.5		
Scenario 4	DLT rate	0.01	0.03	0.06	0.1	0.12	0.25		
	Response rate	0.1	0.2	0.2	0.6	0.45	0.45		
	Mean change of QoL	-20	-18	-14	-6	-8	-10		
ER	Pts(%)	3.4(9%)	4(10.7%)	5.2(13.9%)	10.1(27.2%)	8.4(22.5%)	6.3(16.8%)	37.3	19.5

	Sel%-MTD	0	1	4.3	12.1	26.9	55.7		
	Sel%-RP2D	0	0	0.2	65.8	18	7.8		
AR	Pts(%)	3.4(9%)	4(10.7%)	5.2(14%)	10.1(27.3%)	8.3(22.2%)	6.2(16.7%)	37.1	19.4
	Sel%-MTD	0	1	4.4	12.1	27.3	55.2		
	Sel%-RP2D	0	0	0.2	63.5	19.2	8.7		
PTW	Pts(%)	3.4(9.1%)	4(10.8%)	5.2(14.1%)	10.2(27.6%)	8.1(21.9%)	6.1(16.6%)	37	19.4
	Sel%-MTD	0	1	4.3	12.1	26.9	55.7		
	Sel%-RP2D	0	0	0.2	65.7	18.2	7.7		
MAX	Pts(%)	3.4(9%)	4(10.7%)	5.2(14%)	9.8(26.3%)	8.7(23.5%)	6.2(16.6%)	37.2	19.4
	Sel%-MTD	0	1	4.4	12.2	27.2	55.2		
	Sel%-RP2D	0	0	0.2	66.6	17.4	7.5		
Scenario 5	DLT rate	0.01	0.03	0.06	0.1	0.12	0.25		
	Response rate	0.1	0.15	0.2	0.4	0.4	0.4		
	Mean change of QoL	-4	-8	-12	-18	-20	-20		
ER	Pts(%)	5.7(16%)	6.3(17.7%)	5.7(15.9%)	5.4(15.2%)	6.2(17.5%)	6.3(17.8%)	35.6	19.2
	Sel%-MTD	0	1.4	4.2	12.3	27	55.1		
	Sel%-RP2D	14.1	18.5	5.2	0	0	0		
AR	Pts(%)	5.7(16.1%)	6.3(17.6%)	5.7(16%)	5.4(15.1%)	6.3(17.6%)	6.3(17.6%)	35.6	19.2
	Sel%-MTD	0	1.3	4.6	12.9	27.6	53.6		
	Sel%-RP2D	13.7	18.4	5.9	0	0	0		
PTW	Pts(%)	5.8(16.2%)	6.3(17.6%)	5.6(15.8%)	5.4(15.1%)	6.2(17.5%)	6.3(17.7%)	35.5	19.2
	Sel%-MTD	0	1.3	4.7	12.5	27.1	54.4		
	Sel%-RP2D	14.5	18.4	5.2	0	0	0		
MAX	Pts(%)	5.7(16.1%)	6.3(17.8%)	5.7(16%)	5.4(15.1%)	6.2(17.4%)	6.3(17.6%)	35.6	19.2
	Sel%-MTD	0	1.3	4.5	12.6	26.8	54.8		
	Sel%-RP2D	15	18.4	4.9	0	0	0		

Scenario 6	DLT rate	0.03	0.06	0.12	0.25	0.4	0.55		
	Response rate	0.2	0.2	0.3	0.4	0.4	0.4		
	Mean change of QoL	-14	-14	-18	-20	-20	-20		
ER	Pts(%)	4.3(14.5%)	5.7(19%)	7.8(26%)	7.8(26.2%)	3.5(11.9%)	0.7(2.4%)	29.9	18.4
	Sel%-MTD	0.8	5.9	27.1	51	14.5	0.7		
	Sel%-RP2D	0.9	1.9	0	0	0	0		
AR	Pts(%)	4.3(14.6%)	5.7(19%)	7.8(26.1%)	7.8(26.1%)	3.5(11.8%)	0.7(2.4%)	29.8	18.4
	Sel%-MTD	0.8	5.9	27.2	51	14.4	0.7		
	Sel%-RP2D	0.8	1.9	0	0	0	0		
PTW	Pts(%)	4.3(14.6%)	5.7(19%)	7.8(26.1%)	7.8(26.1%)	3.5(11.8%)	0.7(2.4%)	29.8	18.4
	Sel%-MTD	0.8	5.9	27.5	50.8	14.3	0.7		
	Sel%-RP2D	0.8	1.8	0	0	0	0		
MAX	Pts(%)	4.4(14.6%)	5.7(19%)	7.8(26.1%)	7.8(26.1%)	3.5(11.8%)	0.7(2.4%)	29.8	18.4
	Sel%-MTD	0.8	5.9	27.4	50.9	14.3	0.7		
	Sel%-RP2D	0.9	1.8	0	0	0	0		
Scenario 7	DLT rate	0.03	0.06	0.12	0.25	0.4	0.55		
	Response rate	0.1	0.2	0.4	0.45	0.5	0.5		
	Mean change of QoL	-2	-4	-10	-4	-14	-18		
ER	Pts(%)	6.3(16.7%)	8.9(23.7%)	9.5(25.4%)	9.2(24.4%)	3.2(8.5%)	0.5(1.4%)	37.6	18.3
	Sel%-MTD	0.8	5.3	30.4	50.8	11.7	1		
	Sel%-RP2D	3.9	18.7	21.6	46.5	0.2	0		
AR	Pts(%)	6.3(16.6%)	8.9(23.5%)	9.6(25.3%)	9.3(24.4%)	3.2(8.6%)	0.6(1.6%)	37.9	18.4
	Sel%-MTD	0.8	5.1	30.5	49.9	12.9	0.8		
	Sel%-RP2D	4.4	19.5	23.3	43.7	0.2	0		
PTW	Pts(%)	6.3(16.7%)	8.9(23.6%)	9.4(24.8%)	9.3(24.7%)	3.2(8.6%)	0.6(1.6%)	37.8	18.4
	Sel%-MTD	0.8	5.1	30.1	50	12.9	1.1		

	Sel%-RP2D	4	19.4	23.2	43.8	0.2	0		
MAX	Pts(%)	6.1(16.2%)	8.8(23.3%)	9.7(25.6%)	9.5(25%)	3.2(8.4%)	0.6(1.5%)	37.9	18.3
	Sel%-MTD	0.8	5.1	30	51.5	11.5	1.1		
	Sel%-RP2D	4.6	18.5	23	44.4	0.1	0		
Scenario 8	DLT rate	0.1	0.25	0.4	0.55	0.65	0.65		
	Response rate	0.45	0.45	0.45	0.5	0.5	0.5		
	Mean change of QoL	-2	-6	-12	-18	-20	-20		
ER	Pts(%)	13.3(45.4%)	11.1(37.8%)	4(13.8%)	0.8(2.7%)	0.1(0.2%)	0(0%)	29.3	14.7
	Sel%-MTD	26.1	58.9	13.9	0.7	0	0		
	Sel%-RP2D	65.4	32.2	0.8	0	0	0		
AR	Pts(%)	13.3(45.6%)	10.9(37.6%)	4(13.7%)	0.8(2.8%)	0.1(0.3%)	0(0%)	29.1	14.7
	Sel%-MTD	26.9	57.4	14.1	1.1	0.1	0		
	Sel%-RP2D	65.4	32.1	1	0	0	0		
PTW	Pts(%)	13.3(45.4%)	11(37.5%)	4.1(14%)	0.8(2.8%)	0.1(0.3%)	0(0%)	29.3	14.8
	Sel%-MTD	27	58.4	13.3	0.9	0	0		
	Sel%-RP2D	65.6	32	0.9	0	0	0		
MAX	Pts(%)	13(44.6%)	11.3(38.7%)	4(13.8%)	0.8(2.7%)	0.1(0.2%)	0(0%)	29.1	14.6
	Sel%-MTD	27.7	56.9	14.2	0.8	0	0		
	Sel%-RP2D	65.8	32	0.7	0	0	0		
Scenario 9	DLT rate	0.1	0.25	0.4	0.55	0.65	0.65		
	Response rate	0.2	0.45	0.45	0.5	0.5	0.5		
	Mean change of QoL	-14	-6	-12	-18	-20	-20		
ER	Pts(%)	8.9(35.9%)	11.1(44.8%)	3.9(15.9%)	0.8(3.2%)	0.1(0.2%)	0(0%)	24.8	14.4
	Sel%-MTD	27.4	57.5	14	0.8	0	0		
	Sel%-RP2D	1.2	66	1.4	0	0	0		
AR	Pts(%)	8.9(35.8%)	11.1(44.8%)	4(16%)	0.8(3.2%)	0.1(0.3%)	0(0%)	24.8	14.4

	Sel%-MTD	27.4	57.5	14	0.8	0	0		
	Sel%-RP2D	1.1	66.1	1.3	0	0	0		
PTW	Pts(%)	8.9(36.1%)	11.1(44.9%)	3.9(15.7%)	0.8(3.2%)	0.1(0.2%)	0(0%)	24.8	14.4
	Sel%-MTD	27.5	57.2	14	1	0	0		
	Sel%-RP2D	1.2	65.8	1.4	0	0	0		
MAX	Pts(%)	8.9(35.9%)	11.1(44.8%)	3.9(15.9%)	0.8(3.2%)	0.1(0.2%)	0(0%)	24.7	14.4
	Sel%-MTD	27.4	57.7	13.8	0.8	0	0		
	Sel%-RP2D	1.2	66.1	1.2	0	0	0		
Scenario 10	DLT rate	0.3	0.35	0.4	0.55	0.65	0.65		
	Response rate	0.3	0.3	0.3	0.3	0.3	0.3		
	Mean change of QoL	-14	-15	-16	-18	-20	-20		
ER	Pts(%)	9.3(63.6%)	3.8(26.1%)	1.2(8.3%)	0.3(1.8%)	0(0.2%)	0(0%)	14.6	9.3
	Sel%-MTD	67.2	12.3	3.5	0.5	0	0		
	Sel%-RP2D	4.3	0	0.1	0	0	0		
AR	Pts(%)	9.3(63.6%)	3.8(26.1%)	1.2(8.3%)	0.3(1.8%)	0(0.2%)	0(0%)	14.6	9.3
	Sel%-MTD	67.2	12.3	3.5	0.5	0	0		
	Sel%-RP2D	4.3	0	0.1	0	0	0		
PTW	Pts(%)	9.3(63.6%)	3.8(26.1%)	1.2(8.3%)	0.3(1.8%)	0(0.2%)	0(0%)	14.6	9.3
	Sel%-MTD	67.2	12.3	3.5	0.5	0	0		
	Sel%-RP2D	4.3	0	0.1	0	0	0		
MAX	Pts(%)	9.3(63.6%)	3.8(26.1%)	1.2(8.3%)	0.3(1.8%)	0(0.2%)	0(0%)	14.6	9.3
	Sel%-MTD	67.2	12.3	3.5	0.5	0	0		
	Sel%-RP2D	4.3	0	0.1	0	0	0		

ER, AR, PTW and MAX indicate equal randomization, QoL outcome-based adaptive randomization, pick the winner, and backfilling patients to the highest eligible dose level, respectively.

Sensitivity analysis 2

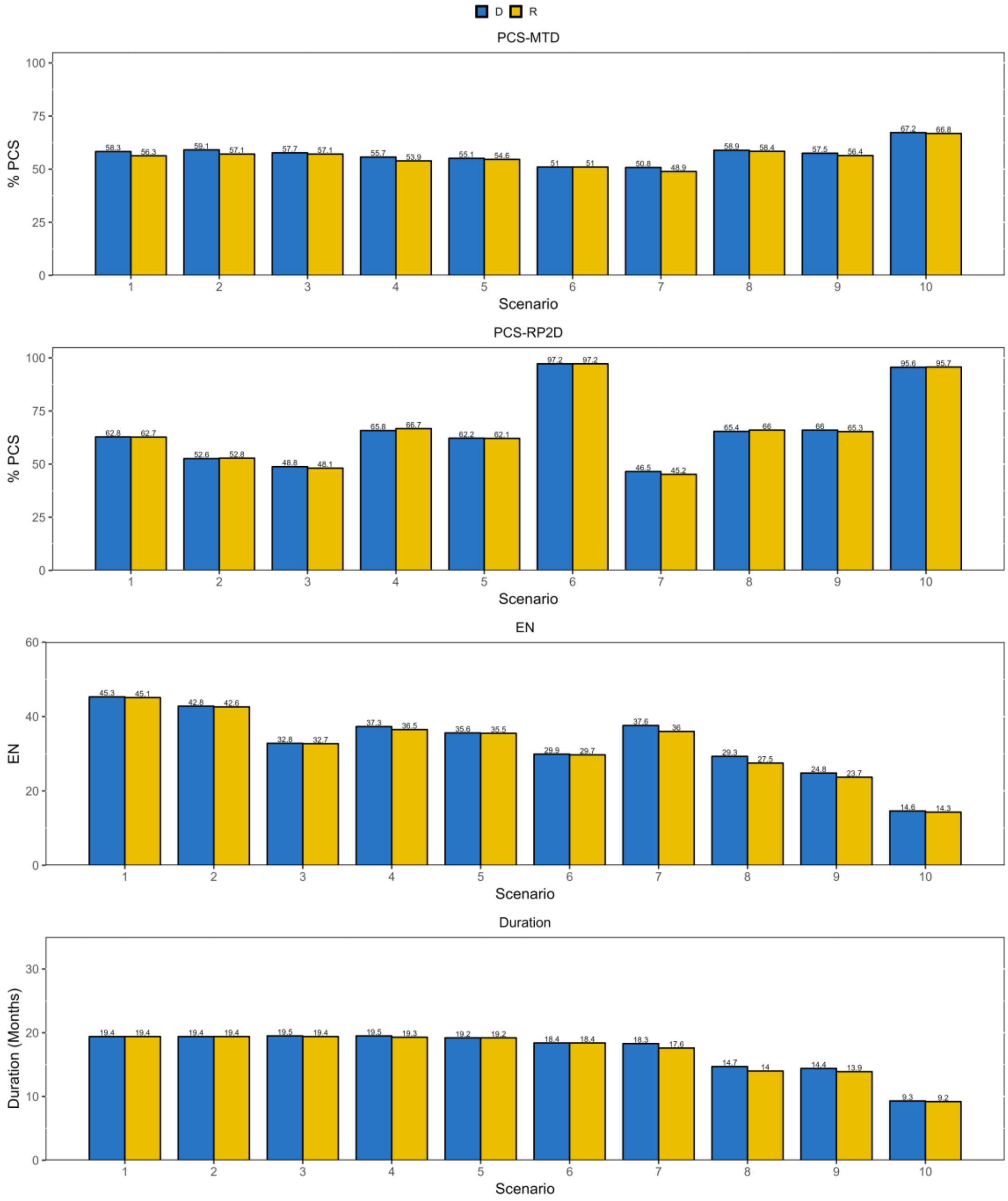

Figure S7 Comparison of two decision-making methods on the percentage of correct selection, the expected sample size and the average trial duration. D indicates the method used in the Backfill-QoL, that is, if there is a de-escalation decision for at least one dose, find the lowest dose corresponding to a de-escalation decision and treat the next dose-escalation cohort according to that decision. R indicates a more conservative method, that is, if there is a retention/de-escalation decision for at least one dose, find the lowest dose corresponding to a retention/de-escalation decision and treat the next dose-escalation cohort according to that decision.

Table S3 Detailed simulation results of sensitivity analysis 2.

Decision-making method		Dose Level						EN	Duration (Month)
		1	2	3	4	5	6		
Scenario 1	DLT rate	0.01	0.03	0.06	0.1	0.12	0.25		
	Response rate	0.45	0.45	0.45	0.5	0.5	0.5		
	Mean change of QoL	0	-6	-10	-14	-18	-20		
D	Pts(%)	10.7(23.7%)	9.2(20.3%)	6.8(15.1%)	5.6(12.4%)	6.3(14%)	6.6(14.5%)	45.3	19.4
	Sel%-MTD	0	2	4.8	10.4	24.5	58.3		
	Sel%-RP2D	62.8	27	6.7	0.8	0	0		
R	Pts(%)	10.7(23.8%)	9.2(20.5%)	6.9(15.3%)	5.6(12.5%)	6.2(13.8%)	6.3(14%)	45.1	19.4
	Sel%-MTD	0	2	5.4	10.5	25.8	56.3		
	Sel%-RP2D	62.7	27.3	6.7	0.6	0	0		
Scenario 2	DLT rate	0.01	0.03	0.06	0.1	0.12	0.25		
	Response rate	0.2	0.45	0.45	0.5	0.5	0.5		
	Mean change of QoL	0	-6	-10	-14	-18	-20		
D	Pts(%)	7.8(18.2%)	9.4(22%)	7.1(16.6%)	5.6(13.1%)	6.3(14.7%)	6.6(15.4%)	42.8	19.4
	Sel%-MTD	0	1.8	5.1	10.4	23.6	59.1		
	Sel%-RP2D	22	52.6	13.2	1.6	0	0.1		
R	Pts(%)	7.8(18.3%)	9.5(22.2%)	7.1(16.7%)	5.7(13.4%)	6.2(14.5%)	6.4(14.9%)	42.6	19.4
	Sel%-MTD	0	1.9	5.4	10.6	25	57.1		
	Sel%-RP2D	21.9	52.8	13.3	1.7	0	0.1		
Scenario 3	DLT rate	0.01	0.03	0.06	0.1	0.12	0.25		
	Response rate	0.1	0.2	0.2	0.3	0.4	0.6		
	Mean change of QoL	-20	-18	-14	-14	-10	-6		
D	Pts(%)	3.4(10.2%)	4(12.1%)	5.2(15.8%)	5.9(17.9%)	8(24.2%)	6.5(19.8%)	32.8	19.5

	Sel%-MTD	0	1	4.3	11.9	25.1	57.7		
	Sel%-RP2D	0	0	0.3	1	18.3	48.8		
R	Pts(%)	3.4(10.3%)	4(12.2%)	5.2(15.9%)	5.9(17.9%)	7.9(24.1%)	6.4(19.6%)	32.7	19.4
	Sel%-MTD	0	1	4.3	11.9	25.7	57.1		
	Sel%-RP2D	0	0	0.4	1.3	18.6	48.1		
Scenario 4	DLT rate	0.01	0.03	0.06	0.1	0.12	0.25		
	Response rate	0.1	0.2	0.2	0.6	0.45	0.45		
	Mean change of QoL	-20	-18	-14	-6	-8	-10		
D	Pts(%)	3.4(9%)	4(10.7%)	5.2(13.9%)	10.1(27.2%)	8.4(22.5%)	6.3(16.8%)	37.3	19.5
	Sel%-MTD	0	1	4.3	12.1	26.9	55.7		
	Sel%-RP2D	0	0	0.2	65.8	18	7.8		
R	Pts(%)	3.4(9.2%)	4(10.9%)	5.2(14.2%)	10.1(27.6%)	8.1(22.1%)	5.8(16%)	36.5	19.3
	Sel%-MTD	0	1	4.3	12.5	28.3	53.9		
	Sel%-RP2D	0	0	0.2	66.7	17.3	7.6		
Scenario 5	DLT rate	0.01	0.03	0.06	0.1	0.12	0.25		
	Response rate	0.1	0.15	0.2	0.4	0.4	0.4		
	Mean change of QoL	-4	-8	-12	-18	-20	-20		
D	Pts(%)	5.7(16%)	6.3(17.7%)	5.7(15.9%)	5.4(15.2%)	6.2(17.5%)	6.3(17.8%)	35.6	19.2
	Sel%-MTD	0	1.4	4.2	12.3	27	55.1		
	Sel%-RP2D	14.1	18.5	5.2	0	0	0		
R	Pts(%)	5.7(16.1%)	6.3(17.8%)	5.7(16.1%)	5.4(15.2%)	6.1(17.2%)	6.2(17.6%)	35.5	19.2
	Sel%-MTD	0	1.4	4.3	13	26.7	54.6		
	Sel%-RP2D	13.9	18.5	5.5	0	0	0		
Scenario 6	DLT rate	0.03	0.06	0.12	0.25	0.4	0.55		
	Response rate	0.2	0.2	0.3	0.4	0.4	0.4		
	Mean change of QoL	-14	-14	-18	-20	-20	-20		

D	Pts(%)	4.3(14.5%)	5.7(19%)	7.8(26%)	7.8(26.2%)	3.5(11.9%)	0.7(2.4%)	29.9	18.4
	Sel%-MTD	0.8	5.9	27.1	51	14.5	0.7		
	Sel%-RP2D	0.9	1.9	0	0	0	0		
R	Pts(%)	4.4(14.6%)	5.7(19.2%)	7.7(26%)	7.7(26%)	3.5(11.8%)	0.7(2.3%)	29.7	18.4
	Sel%-MTD	0.8	5.9	27.4	51	14.3	0.6		
	Sel%-RP2D	0.9	1.9	0	0	0	0		
Scenario 7	DLT rate	0.03	0.06	0.12	0.25	0.4	0.55		
	Response rate	0.1	0.2	0.4	0.45	0.5	0.5		
	Mean change of QoL	-2	-4	-10	-4	-14	-18		
D	Pts(%)	6.3(16.7%)	8.9(23.7%)	9.5(25.4%)	9.2(24.4%)	3.2(8.5%)	0.5(1.4%)	37.6	18.3
	Sel%-MTD	0.8	5.3	30.4	50.8	11.7	1		
	Sel%-RP2D	3.9	18.7	21.6	46.5	0.2	0		
R	Pts(%)	6.3(17.5%)	8.8(24.5%)	9.1(25.3%)	8.5(23.6%)	2.9(8%)	0.4(1.1%)	36	17.6
	Sel%-MTD	0.8	5.9	30.1	48.9	13.4	0.9		
	Sel%-RP2D	4.1	18.8	22	45.2	0.4	0		
Scenario 8	DLT rate	0.1	0.25	0.4	0.55	0.65	0.65		
	Response rate	0.45	0.45	0.45	0.5	0.5	0.5		
	Mean change of QoL	-2	-6	-12	-18	-20	-20		
D	Pts(%)	13.3(45.4%)	11.1(37.8%)	4(13.8%)	0.8(2.7%)	0.1(0.2%)	0(0%)	29.3	14.7
	Sel%-MTD	26.1	58.9	13.9	0.7	0	0		
	Sel%-RP2D	65.4	32.2	0.8	0	0	0		
R	Pts(%)	12.9(46.8%)	10.4(37.9%)	3.6(13%)	0.6(2.2%)	0(0.1%)	0(0%)	27.5	14
	Sel%-MTD	26.3	58.4	13.8	1.2	0	0		
	Sel%-RP2D	66	31.6	1.1	0	0	0		
Scenario 9	DLT rate	0.1	0.25	0.4	0.55	0.65	0.65		
	Response rate	0.2	0.45	0.45	0.5	0.5	0.5		

	Mean change of QoL	-14	-6	-12	-18	-20	-20		
D	Pts(%)	8.9(35.9%)	11.1(44.8%)	3.9(15.9%)	0.8(3.2%)	0.1(0.2%)	0(0%)	24.8	14.4
	Sel%-MTD	27.4	57.5	14	0.8	0	0		
	Sel%-RP2D	1.2	66	1.4	0	0	0		
R	Pts(%)	8.6(36.1%)	10.7(45.1%)	3.7(15.7%)	0.7(2.9%)	0(0.2%)	0(0%)	23.7	13.9
	Sel%-MTD	27.4	56.4	15	0.9	0	0		
	Sel%-RP2D	1.3	65.3	2	0	0	0		
Scenario 10	DLT rate	0.3	0.35	0.4	0.55	0.65	0.65		
	Response rate	0.3	0.3	0.3	0.3	0.3	0.3		
	Mean change of QoL	-14	-15	-16	-18	-20	-20		
D	Pts(%)	9.3(63.6%)	3.8(26.1%)	1.2(8.3%)	0.3(1.8%)	0(0.2%)	0(0%)	14.6	9.3
	Sel%-MTD	67.2	12.3	3.5	0.5	0	0		
	Sel%-RP2D	4.3	0	0.1	0	0	0		
R	Pts(%)	9.2(63.9%)	3.8(26.2%)	1.1(8%)	0.3(1.8%)	0(0.2%)	0(0%)	14.3	9.2
	Sel%-MTD	66.8	12.6	3.5	0.5	0	0		
	Sel%-RP2D	4.2	0	0.1	0	0	0		

D indicates the method used in the Backfill-QoL, that is, if there is a de-escalation decision for at least one dose, find the lowest dose corresponding to a de-escalation decision and treat the next dose-escalation cohort according to that decision. R indicates a more conservative method, that is, if there is a retention/de-escalation decision for at least one dose, find the lowest dose corresponding to a retention/de-escalation decision and treat the next dose-escalation cohort according to that decision.

Sensitivity analysis 3

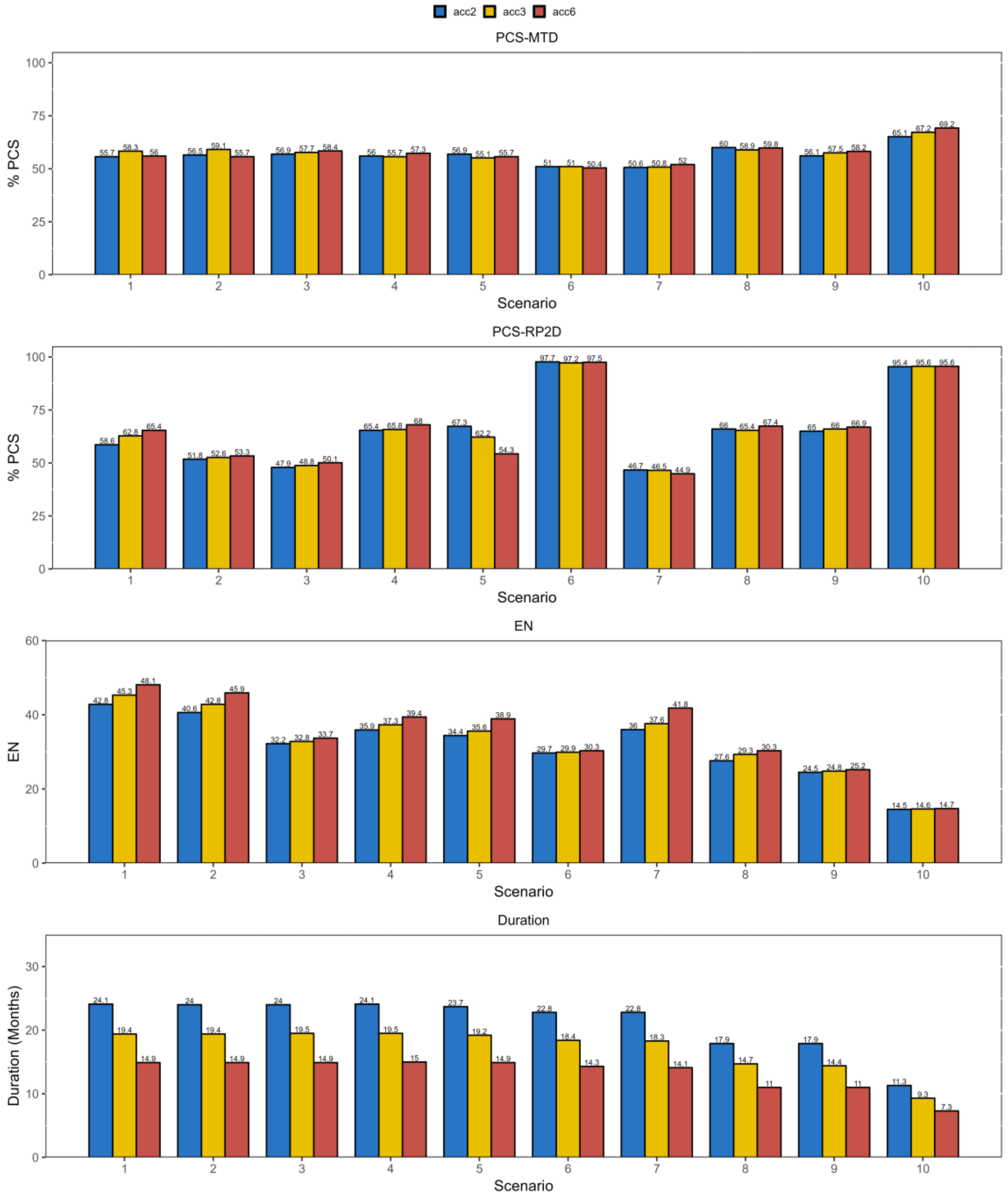

Figure S8 Comparison of three accrual rates on the percentage of correct selection, the expected sample size and the average trial duration. The labels acc2, acc3 and acc6 indicate the accrual rate of 2, 3, and 6 patients per 4 weeks, respectively.

Table S4 Detailed simulation results of sensitivity analysis 3.

Accrual rate		Dose Level 1	2	3	4	5	6	EN	Duration (Month)
Scenario 1	DLT rate	0.01	0.03	0.06	0.1	0.12	0.25		
	Response rate	0.45	0.45	0.45	0.5	0.5	0.5		
	Mean change of QoL	0	-6	-10	-14	-18	-20		
acc2	Pts(%)	9.7(22.7%)	8.2(19.1%)	6.6(15.3%)	5.7(13.4%)	6.2(14.6%)	6.3(14.8%)	42.8	24.1
	Sel%-MTD	0	1.5	4.8	13.3	24.7	55.7		
	Sel%-RP2D	58.6	29.4	7.4	1.3	0	0		
acc3	Pts(%)	10.7(23.7%)	9.2(20.3%)	6.8(15.1%)	5.6(12.4%)	6.3(14%)	6.6(14.5%)	45.3	19.4
	Sel%-MTD	0	2	4.8	10.4	24.5	58.3		
	Sel%-RP2D	62.8	27	6.7	0.8	0	0		
acc6	Pts(%)	11.6(24.1%)	10.1(21%)	7.7(16.1%)	5.9(12.3%)	6.3(13.2%)	6.4(13.3%)	48.1	14.9
	Sel%-MTD	0	1.8	4.4	13.2	24.6	56		
	Sel%-RP2D	65.4	27.1	5.8	0.4	0	0		
Scenario 2	DLT rate	0.01	0.03	0.06	0.1	0.12	0.25		
	Response rate	0.2	0.45	0.45	0.5	0.5	0.5		
	Mean change of QoL	0	-6	-10	-14	-18	-20		
acc2	Pts(%)	6.9(17%)	8.6(21.3%)	6.6(16.3%)	5.7(13.9%)	6.4(15.7%)	6.4(15.8%)	40.6	24
	Sel%-MTD	0	1.3	4.9	11.6	25.7	56.5		
	Sel%-RP2D	20.7	51.8	12.7	1.6	0	0		
acc3	Pts(%)	7.8(18.2%)	9.4(22%)	7.1(16.6%)	5.6(13.1%)	6.3(14.7%)	6.6(15.4%)	42.8	19.4
	Sel%-MTD	0	1.8	5.1	10.4	23.6	59.1		
	Sel%-RP2D	22	52.6	13.2	1.6	0	0.1		
acc6	Pts(%)	9.4(20.5%)	10.2(22.2%)	7.9(17.2%)	5.9(12.9%)	6.2(13.6%)	6.3(13.8%)	45.9	14.9
	Sel%-MTD	0	1.4	5.1	13.6	24.2	55.7		

	Sel%-RP2D	26.3	53.3	11.4	0.7	0	0		
Scenario 3	DLT rate	0.01	0.03	0.06	0.1	0.12	0.25		
	Response rate	0.1	0.2	0.2	0.3	0.4	0.6		
	Mean change of QoL	-20	-18	-14	-14	-10	-6		
acc2	Pts(%)	3.4(10.4%)	4(12.4%)	5.1(15.9%)	5.7(17.8%)	7.5(23.4%)	6.5(20.1%)	32.2	24
	Sel%-MTD	0	1	4.5	11.9	25.7	56.9		
	Sel%-RP2D	0	0	0.2	1.3	19.9	47.9		
acc3	Pts(%)	3.4(10.2%)	4(12.1%)	5.2(15.8%)	5.9(17.9%)	8(24.2%)	6.5(19.8%)	32.8	19.5
	Sel%-MTD	0	1	4.3	11.9	25.1	57.7		
	Sel%-RP2D	0	0	0.3	1	18.3	48.8		
acc6	Pts(%)	3.4(10%)	4(11.9%)	5.4(16.1%)	6(17.7%)	8.4(25%)	6.5(19.4%)	33.7	14.9
	Sel%-MTD	0	1	4.2	11.8	24.6	58.4		
	Sel%-RP2D	0	0	0.4	1	16.7	50.1		
Scenario 4	DLT rate	0.01	0.03	0.06	0.1	0.12	0.25		
	Response rate	0.1	0.2	0.2	0.6	0.45	0.45		
	Mean change of QoL	-20	-18	-14	-6	-8	-10		
acc2	Pts(%)	3.4(9.3%)	4(11.1%)	5.1(14.3%)	9.4(26.2%)	7.7(21.4%)	6.3(17.6%)	35.9	24.1
	Sel%-MTD	0	1	4.5	11.9	26.6	56		
	Sel%-RP2D	0	0	0.2	65.4	17.1	8.3		
acc3	Pts(%)	3.4(9%)	4(10.7%)	5.2(13.9%)	10.1(27.2%)	8.4(22.5%)	6.3(16.8%)	37.3	19.5
	Sel%-MTD	0	1	4.3	12.1	26.9	55.7		
	Sel%-RP2D	0	0	0.2	65.8	18	7.8		
acc6	Pts(%)	3.4(8.6%)	4(10.2%)	5.5(13.9%)	10.8(27.6%)	9.3(23.6%)	6.4(16.2%)	39.4	15
	Sel%-MTD	0	1	4.2	12.3	25.2	57.3		
	Sel%-RP2D	0	0	0.1	68	16.4	7.5		
Scenario 5	DLT rate	0.01	0.03	0.06	0.1	0.12	0.25		

	Response rate	0.1	0.15	0.2	0.4	0.4	0.4		
	Mean change of QoL	-4	-8	-12	-18	-20	-20		
acc2	Pts(%)	5.2(15%)	5.8(16.9%)	5.5(15.9%)	5.5(15.9%)	6.2(18%)	6.3(18.4%)	34.4	23.7
	Sel%-MTD	0	1.1	3.7	14.1	24.2	56.9		
	Sel%-RP2D	11.4	15.7	5.6	0	0	0		
acc3	Pts(%)	5.7(16%)	6.3(17.7%)	5.7(15.9%)	5.4(15.2%)	6.2(17.5%)	6.3(17.8%)	35.6	19.2
	Sel%-MTD	0	1.4	4.2	12.3	27	55.1		
	Sel%-RP2D	14.1	18.5	5.2	0	0	0		
acc6	Pts(%)	7.5(19.2%)	7.2(18.4%)	6.1(15.6%)	5.4(13.9%)	6.4(16.4%)	6.4(16.5%)	38.9	14.9
	Sel%-MTD	0	1.4	5.5	10.7	26.7	55.7		
	Sel%-RP2D	20.2	21	4.5	0	0	0		
Scenario 6	DLT rate	0.03	0.06	0.12	0.25	0.4	0.55		
	Response rate	0.2	0.2	0.3	0.4	0.4	0.4		
	Mean change of QoL	-14	-14	-18	-20	-20	-20		
acc2	Pts(%)	4.3(14.4%)	5.6(18.7%)	7.8(26.2%)	7.8(26.4%)	3.5(11.9%)	0.7(2.4%)	29.7	22.8
	Sel%-MTD	0.8	5.9	27.5	51	14	0.8		
	Sel%-RP2D	1.1	1.2	0	0	0	0		
acc3	Pts(%)	4.3(14.5%)	5.7(19%)	7.8(26%)	7.8(26.2%)	3.5(11.9%)	0.7(2.4%)	29.9	18.4
	Sel%-MTD	0.8	5.9	27.1	51	14.5	0.7		
	Sel%-RP2D	0.9	1.9	0	0	0	0		
acc6	Pts(%)	4.5(14.9%)	5.8(19.3%)	7.8(25.6%)	7.8(25.8%)	3.6(11.9%)	0.8(2.5%)	30.3	14.3
	Sel%-MTD	0.8	5.6	27.9	50.4	14.7	0.6		
	Sel%-RP2D	1.6	0.9	0	0	0	0		
Scenario 7	DLT rate	0.03	0.06	0.12	0.25	0.4	0.55		
	Response rate	0.1	0.2	0.4	0.45	0.5	0.5		
	Mean change of QoL	-2	-4	-10	-4	-14	-18		

acc2	Pts(%)	5.7(15.8%)	8.2(22.9%)	9.2(25.5%)	9(24.9%)	3.4(9.4%)	0.5(1.5%)	36	22.8
	Sel%-MTD	0.8	4.9	30	50.6	12.7	1		
	Sel%-RP2D	3.5	15.5	24.1	46.7	0.5	0		
acc3	Pts(%)	6.3(16.7%)	8.9(23.7%)	9.5(25.4%)	9.2(24.4%)	3.2(8.5%)	0.5(1.4%)	37.6	18.3
	Sel%-MTD	0.8	5.3	30.4	50.8	11.7	1		
	Sel%-RP2D	3.9	18.7	21.6	46.5	0.2	0		
acc6	Pts(%)	8(19.1%)	10.1(24.2%)	10.4(24.8%)	9.8(23.5%)	3(7.2%)	0.5(1.2%)	41.8	14.1
	Sel%-MTD	0.8	6.1	29.5	52	10.5	1.1		
	Sel%-RP2D	5.9	17.9	23.2	44.9	0.3	0		
Scenario 8	DLT rate	0.1	0.25	0.4	0.55	0.65	0.65		
	Response rate	0.45	0.45	0.45	0.5	0.5	0.5		
	Mean change of QoL	-2	-6	-12	-18	-20	-20		
acc2	Pts(%)	12.4(45%)	10.5(38%)	3.9(14.2%)	0.7(2.6%)	0.1(0.2%)	0(0%)	27.6	17.9
	Sel%-MTD	25.8	60	13.1	0.7	0	0		
	Sel%-RP2D	66	31.7	0.7	0.1	0	0		
acc3	Pts(%)	13.3(45.4%)	11.1(37.8%)	4(13.8%)	0.8(2.7%)	0.1(0.2%)	0(0%)	29.3	14.7
	Sel%-MTD	26.1	58.9	13.9	0.7	0	0		
	Sel%-RP2D	65.4	32.2	0.8	0	0	0		
acc6	Pts(%)	14.2(46.7%)	11.6(38.1%)	3.9(12.8%)	0.7(2.2%)	0.1(0.2%)	0(0%)	30.3	11
	Sel%-MTD	27	59.8	12.2	0.7	0	0		
	Sel%-RP2D	67.4	31.3	0.5	0	0	0		
Scenario 9	DLT rate	0.1	0.25	0.4	0.55	0.65	0.65		
	Response rate	0.2	0.45	0.45	0.5	0.5	0.5		
	Mean change of QoL	-14	-6	-12	-18	-20	-20		
acc2	Pts(%)	8.8(35.9%)	10.9(44.3%)	4(16.4%)	0.8(3.1%)	0.1(0.3%)	0(0%)	24.5	17.9
	Sel%-MTD	27.7	56.1	14.9	0.8	0.1	0		

	Sel%-RP2D	1.4	65	1.8	0.1	0	0		
acc3	Pts(%)	8.9(35.9%)	11.1(44.8%)	3.9(15.9%)	0.8(3.2%)	0.1(0.2%)	0(0%)	24.8	14.4
	Sel%-MTD	27.4	57.5	14	0.8	0	0		
	Sel%-RP2D	1.2	66	1.4	0	0	0		
acc6	Pts(%)	9(35.8%)	11.5(45.8%)	3.9(15.4%)	0.7(2.8%)	0.1(0.2%)	0(0%)	25.2	11
	Sel%-MTD	27.3	58.2	13.9	0.3	0	0		
	Sel%-RP2D	1.2	66.9	0.8	0	0	0		
Scenario 10	DLT rate	0.3	0.35	0.4	0.55	0.65	0.65		
	Response rate	0.3	0.3	0.3	0.3	0.3	0.3		
	Mean change of QoL	-14	-15	-16	-18	-20	-20		
acc2	Pts(%)	9.2(63.4%)	3.8(26.1%)	1.2(8.4%)	0.3(1.9%)	0(0.2%)	0(0%)	14.5	11.3
	Sel%-MTD	65.1	12.3	3.9	0.5	0	0		
	Sel%-RP2D	4.4	0.1	0.1	0	0	0		
acc3	Pts(%)	9.3(63.6%)	3.8(26.1%)	1.2(8.3%)	0.3(1.8%)	0(0.2%)	0(0%)	14.6	9.3
	Sel%-MTD	67.2	12.3	3.5	0.5	0	0		
	Sel%-RP2D	4.3	0	0.1	0	0	0		
acc6	Pts(%)	9.3(63.5%)	3.8(25.8%)	1.2(8.4%)	0.3(2%)	0(0.3%)	0(0%)	14.7	7.3
	Sel%-MTD	69.2	12.5	3.5	0.5	0	0		
	Sel%-RP2D	4.1	0.2	0.1	0	0	0		

The labels acc2, acc3 and acc6 indicate the accrual rate of 2, 3, and 6 patients per 4 weeks, respectively.

It is worth noting that in scenario 5, where dose levels 1 and 2 have low response rates but favorable QoL profiles, a high accrual rate results in a reduced percentage of not selecting any dose as RP2D. This is because the design fails to eliminate ineffective doses in time due to the inconsistency between efficacy and QoL, and more patients are backfilled to dose levels 1 and 2. Meanwhile, the futility boundaries are all 0 when

the sample size is less than 8, so the efficacy monitoring criterion is more easily reached when the number of evaluable patients increases within 7. To address this issue, we can set stricter monitoring rules, and/or continue to closely monitor drug efficacy in the subsequent trials.

Sensitivity analysis 4

We showed how design performance changes with those statistical cutoffs in this section, including φ_E (Sensitivity analysis 4A), φ_Q (Sensitivity analysis 4B), and φ_C (Sensitivity analysis 4C).

Sensitivity analysis 4A

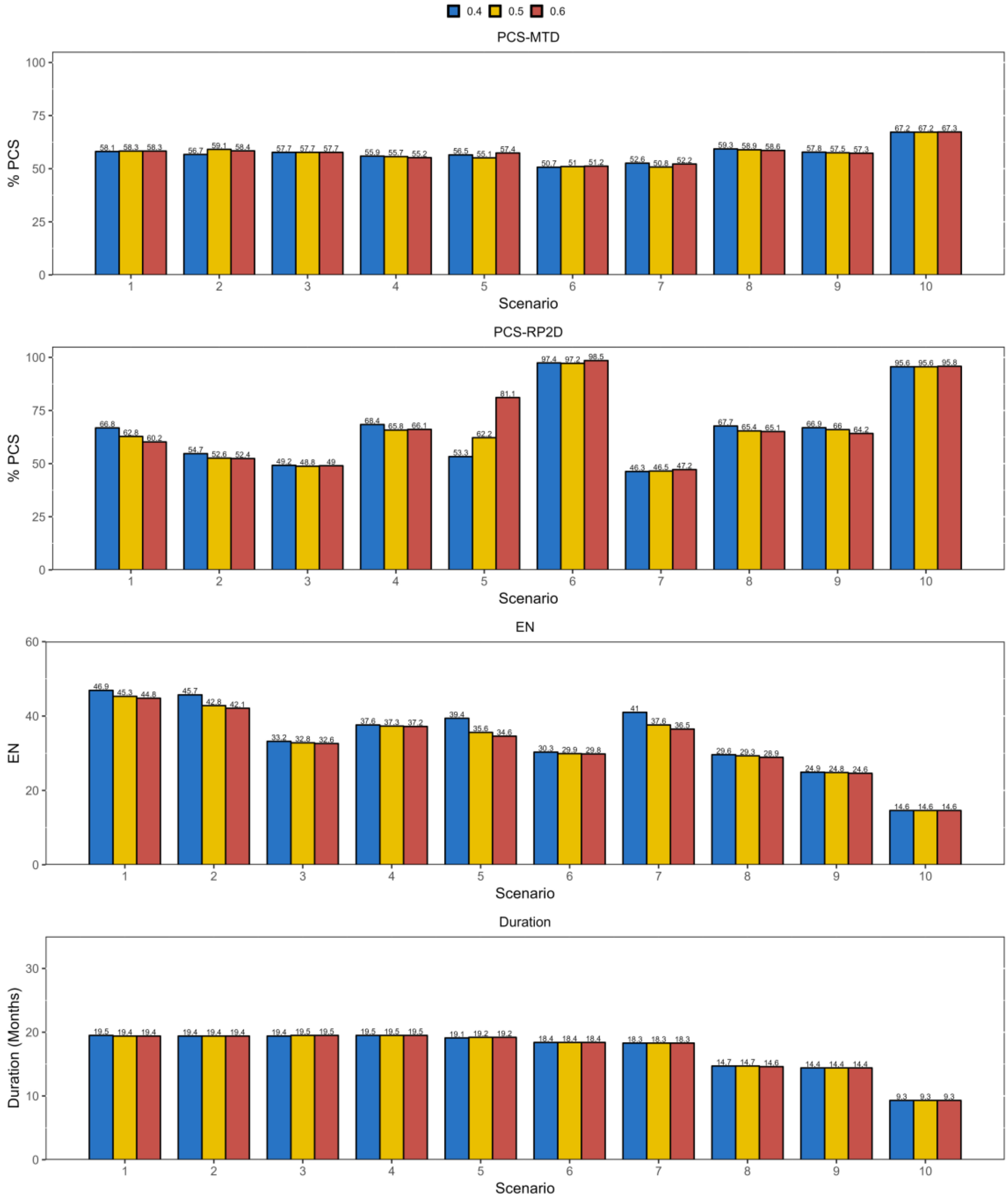

Figure S9 Comparison of three cutoff values for φ_E on the percentage of correct selection, the expected sample size and the average trial duration.

Table S5 Detailed simulation results of sensitivity analysis 4A.

Cutoff φ_E		Dose Level						EN	Duration (Month)
		1	2	3	4	5	6		
Scenario 1	DLT rate	0.01	0.03	0.06	0.1	0.12	0.25		
	Response rate	0.45	0.45	0.45	0.5	0.5	0.5		
	Mean change of QoL	0	-6	-10	-14	-18	-20		
0.4	Pts(%)	11.5(24.6%)	9.7(20.7%)	7(14.9%)	5.6(12%)	6.4(13.6%)	6.6(14.1%)	46.9	19.5
	Sel%-MTD	0	2	4.3	11.5	24.1	58.1		
	Sel%-RP2D	66.8	26.5	5.5	0.8	0	0		
0.5	Pts(%)	10.7(23.7%)	9.2(20.3%)	6.8(15.1%)	5.6(12.4%)	6.3(14%)	6.6(14.5%)	45.3	19.4
	Sel%-MTD	0	2	4.8	10.4	24.5	58.3		
	Sel%-RP2D	62.8	27	6.7	0.8	0	0		
0.6	Pts(%)	10.5(23.4%)	9(20.1%)	6.8(15.1%)	5.6(12.6%)	6.4(14.2%)	6.6(14.6%)	44.8	19.4
	Sel%-MTD	0	2	4.8	10.8	24.1	58.3		
	Sel%-RP2D	60.2	28.6	6.6	0.8	0	0		
Scenario 2	DLT rate	0.01	0.03	0.06	0.1	0.12	0.25		
	Response rate	0.2	0.45	0.45	0.5	0.5	0.5		
	Mean change of QoL	0	-6	-10	-14	-18	-20		
0.4	Pts(%)	9.9(21.6%)	9.9(21.7%)	7.2(15.7%)	5.7(12.6%)	6.4(14.1%)	6.5(14.3%)	45.7	19.4
	Sel%-MTD	0	1.9	4.5	11.7	25.2	56.7		
	Sel%-RP2D	27.2	54.7	11.2	1	0	0		
0.5	Pts(%)	7.8(18.2%)	9.4(22%)	7.1(16.6%)	5.6(13.1%)	6.3(14.7%)	6.6(15.4%)	42.8	19.4
	Sel%-MTD	0	1.8	5.1	10.4	23.6	59.1		
	Sel%-RP2D	22	52.6	13.2	1.6	0	0.1		
0.6	Pts(%)	7.1(16.8%)	9.3(22%)	7.2(17.1%)	5.7(13.4%)	6.3(15%)	6.6(15.6%)	42.1	19.4
	Sel%-MTD	0	1.7	4.9	10.2	24.8	58.4		

	Sel%-RP2D	14.9	52.4	14.8	1.8	0.1	0		
Scenario 3	DLT rate	0.01	0.03	0.06	0.1	0.12	0.25		
	Response rate	0.1	0.2	0.2	0.3	0.4	0.6		
	Mean change of QoL	-20	-18	-14	-14	-10	-6		
0.4	Pts(%)	3.4(10.1%)	4(12.1%)	5.4(16.3%)	6(17.9%)	8(24.2%)	6.4(19.4%)	33.2	19.4
	Sel%-MTD	0	1	4.3	12.2	24.8	57.7		
	Sel%-RP2D	0	0	0.5	1.2	17.3	49.2		
0.5	Pts(%)	3.4(10.2%)	4(12.1%)	5.2(15.8%)	5.9(17.9%)	8(24.2%)	6.5(19.8%)	32.8	19.5
	Sel%-MTD	0	1	4.3	11.9	25.1	57.7		
	Sel%-RP2D	0	0	0.3	1	18.3	48.8		
0.6	Pts(%)	3.4(10.3%)	4(12.2%)	5.2(15.8%)	5.8(17.8%)	7.8(24%)	6.5(19.9%)	32.6	19.5
	Sel%-MTD	0	1	4.3	11.8	25.2	57.7		
	Sel%-RP2D	0	0	0.4	1.1	17.7	49		
Scenario 4	DLT rate	0.01	0.03	0.06	0.1	0.12	0.25		
	Response rate	0.1	0.2	0.2	0.6	0.45	0.45		
	Mean change of QoL	-20	-18	-14	-6	-8	-10		
0.4	Pts(%)	3.4(9%)	4(10.6%)	5.4(14.4%)	10.2(27.2%)	8.3(22.2%)	6.2(16.5%)	37.6	19.5
	Sel%-MTD	0	1	4.3	12.2	26.6	55.9		
	Sel%-RP2D	0	0	0.3	68.4	16.3	7.4		
0.5	Pts(%)	3.4(9%)	4(10.7%)	5.2(13.9%)	10.1(27.2%)	8.4(22.5%)	6.3(16.8%)	37.3	19.5
	Sel%-MTD	0	1	4.3	12.1	26.9	55.7		
	Sel%-RP2D	0	0	0.2	65.8	18	7.8		
0.6	Pts(%)	3.4(9%)	4(10.7%)	5.2(13.9%)	10.1(27.2%)	8.3(22.3%)	6.2(16.8%)	37.2	19.5
	Sel%-MTD	0	1	4.3	12.2	27.3	55.2		
	Sel%-RP2D	0	0	0.1	66.1	17.9	7.8		
Scenario 5	DLT rate	0.01	0.03	0.06	0.1	0.12	0.25		

	Response rate	0.1	0.15	0.2	0.4	0.4	0.4		
	Mean change of QoL	-4	-8	-12	-18	-20	-20		
0.4	Pts(%)	8.2(20.7%)	7.4(18.9%)	6(15.2%)	5.3(13.4%)	6.2(15.6%)	6.4(16.2%)	39.4	19.1
	Sel%-MTD	0	1.8	4.5	12.2	25	56.5		
	Sel%-RP2D	19.9	21.9	4.9	0	0	0		
0.5	Pts(%)	5.7(16%)	6.3(17.7%)	5.7(15.9%)	5.4(15.2%)	6.2(17.5%)	6.3(17.8%)	35.6	19.2
	Sel%-MTD	0	1.4	4.2	12.3	27	55.1		
	Sel%-RP2D	14.1	18.5	5.2	0	0	0		
0.6	Pts(%)	5.3(15.3%)	5.8(16.8%)	5.5(16%)	5.3(15.4%)	6.2(18%)	6.4(18.5%)	34.6	19.2
	Sel%-MTD	0	1.4	4	11.8	25.4	57.4		
	Sel%-RP2D	5.7	9.1	4.1	0	0	0		
Scenario 6	DLT rate	0.03	0.06	0.12	0.25	0.4	0.55		
	Response rate	0.2	0.2	0.3	0.4	0.4	0.4		
	Mean change of QoL	-14	-14	-18	-20	-20	-20		
0.4	Pts(%)	4.6(15.1%)	5.9(19.3%)	7.8(25.6%)	7.8(25.7%)	3.6(11.9%)	0.7(2.5%)	30.3	18.4
	Sel%-MTD	0.8	5.5	27.7	50.7	14.8	0.5		
	Sel%-RP2D	1	1.6	0	0	0	0		
0.5	Pts(%)	4.3(14.5%)	5.7(19%)	7.8(26%)	7.8(26.2%)	3.5(11.9%)	0.7(2.4%)	29.9	18.4
	Sel%-MTD	0.8	5.9	27.1	51	14.5	0.7		
	Sel%-RP2D	0.9	1.9	0	0	0	0		
0.6	Pts(%)	4.3(14.5%)	5.6(18.9%)	7.8(26.1%)	7.8(26.2%)	3.5(11.8%)	0.7(2.4%)	29.8	18.4
	Sel%-MTD	0.8	5.9	27	51.2	14.4	0.7		
	Sel%-RP2D	0.4	1.1	0	0	0	0		
Scenario 7	DLT rate	0.03	0.06	0.12	0.25	0.4	0.55		
	Response rate	0.1	0.2	0.4	0.45	0.5	0.5		
	Mean change of QoL	-2	-4	-10	-4	-14	-18		

0.4	Pts(%)	8.4(20.6%)	10(24.5%)	9.6(23.3%)	9.2(22.5%)	3.2(7.7%)	0.6(1.4%)	41	18.3
	Sel%-MTD	0.8	5.5	28.3	52.6	12.2	0.6		
	Sel%-RP2D	5.3	18.4	22	46.3	0.4	0		
0.5	Pts(%)	6.3(16.7%)	8.9(23.7%)	9.5(25.4%)	9.2(24.4%)	3.2(8.5%)	0.5(1.4%)	37.6	18.3
	Sel%-MTD	0.8	5.3	30.4	50.8	11.7	1		
	Sel%-RP2D	3.9	18.7	21.6	46.5	0.2	0		
0.6	Pts(%)	5.7(15.7%)	8.3(22.8%)	9.5(26%)	9.2(25.1%)	3.2(8.9%)	0.5(1.5%)	36.5	18.3
	Sel%-MTD	0.8	5.3	29.6	52.2	11.6	0.5		
	Sel%-RP2D	2.3	14.5	22.2	47.2	0.3	0		
Scenario 8	DLT rate	0.1	0.25	0.4	0.55	0.65	0.65		
	Response rate	0.45	0.45	0.45	0.5	0.5	0.5		
	Mean change of QoL	-2	-6	-12	-18	-20	-20		
0.4	Pts(%)	13.7(46.2%)	11.1(37.5%)	4(13.6%)	0.7(2.5%)	0.1(0.2%)	0(0%)	29.6	14.7
	Sel%-MTD	27	59.3	12.6	0.8	0	0		
	Sel%-RP2D	67.7	31	0.6	0	0	0		
0.5	Pts(%)	13.3(45.4%)	11.1(37.8%)	4(13.8%)	0.8(2.7%)	0.1(0.2%)	0(0%)	29.3	14.7
	Sel%-MTD	26.1	58.9	13.9	0.7	0	0		
	Sel%-RP2D	65.4	32.2	0.8	0	0	0		
0.6	Pts(%)	13.1(45.3%)	10.9(37.8%)	4(13.9%)	0.8(2.8%)	0.1(0.2%)	0(0%)	28.9	14.6
	Sel%-MTD	26.7	58.6	13.7	0.6	0	0		
	Sel%-RP2D	65.1	32.2	0.9	0	0	0		
Scenario 9	DLT rate	0.1	0.25	0.4	0.55	0.65	0.65		
	Response rate	0.2	0.45	0.45	0.5	0.5	0.5		
	Mean change of QoL	-14	-6	-12	-18	-20	-20		
0.4	Pts(%)	9(36%)	11.2(45%)	3.9(15.8%)	0.8(3.1%)	0.1(0.2%)	0(0%)	24.9	14.4
	Sel%-MTD	27.1	57.8	14	0.8	0	0		

	Sel%-RP2D	1.3	66.9	1.4	0	0	0		
0.5	Pts(%)	8.9(35.9%)	11.1(44.8%)	3.9(15.9%)	0.8(3.2%)	0.1(0.2%)	0(0%)	24.8	14.4
	Sel%-MTD	27.4	57.5	14	0.8	0	0		
	Sel%-RP2D	1.2	66	1.4	0	0	0		
0.6	Pts(%)	8.9(36%)	11(44.7%)	3.9(15.9%)	0.8(3.2%)	0(0.2%)	0(0%)	24.6	14.4
	Sel%-MTD	27.5	57.3	14.1	0.8	0	0		
	Sel%-RP2D	1.1	64.2	1.4	0	0	0		
Scenario 10	DLT rate	0.3	0.35	0.4	0.55	0.65	0.65		
	Response rate	0.3	0.3	0.3	0.3	0.3	0.3		
	Mean change of QoL	-14	-15	-16	-18	-20	-20		
0.4	Pts(%)	9.3(63.6%)	3.8(26.1%)	1.2(8.3%)	0.3(1.8%)	0(0.2%)	0(0%)	14.6	9.3
	Sel%-MTD	67.2	12.1	3.7	0.4	0	0		
	Sel%-RP2D	4.3	0	0.1	0	0	0		
0.5	Pts(%)	9.3(63.6%)	3.8(26.1%)	1.2(8.3%)	0.3(1.8%)	0(0.2%)	0(0%)	14.6	9.3
	Sel%-MTD	67.2	12.3	3.5	0.5	0	0		
	Sel%-RP2D	4.3	0	0.1	0	0	0		
0.6	Pts(%)	9.3(63.5%)	3.8(26.1%)	1.2(8.3%)	0.3(1.8%)	0(0.2%)	0(0%)	14.6	9.3
	Sel%-MTD	67.3	12.2	3.5	0.5	0	0		
	Sel%-RP2D	4.1	0	0.1	0	0	0		

Sensitivity analysis 4B

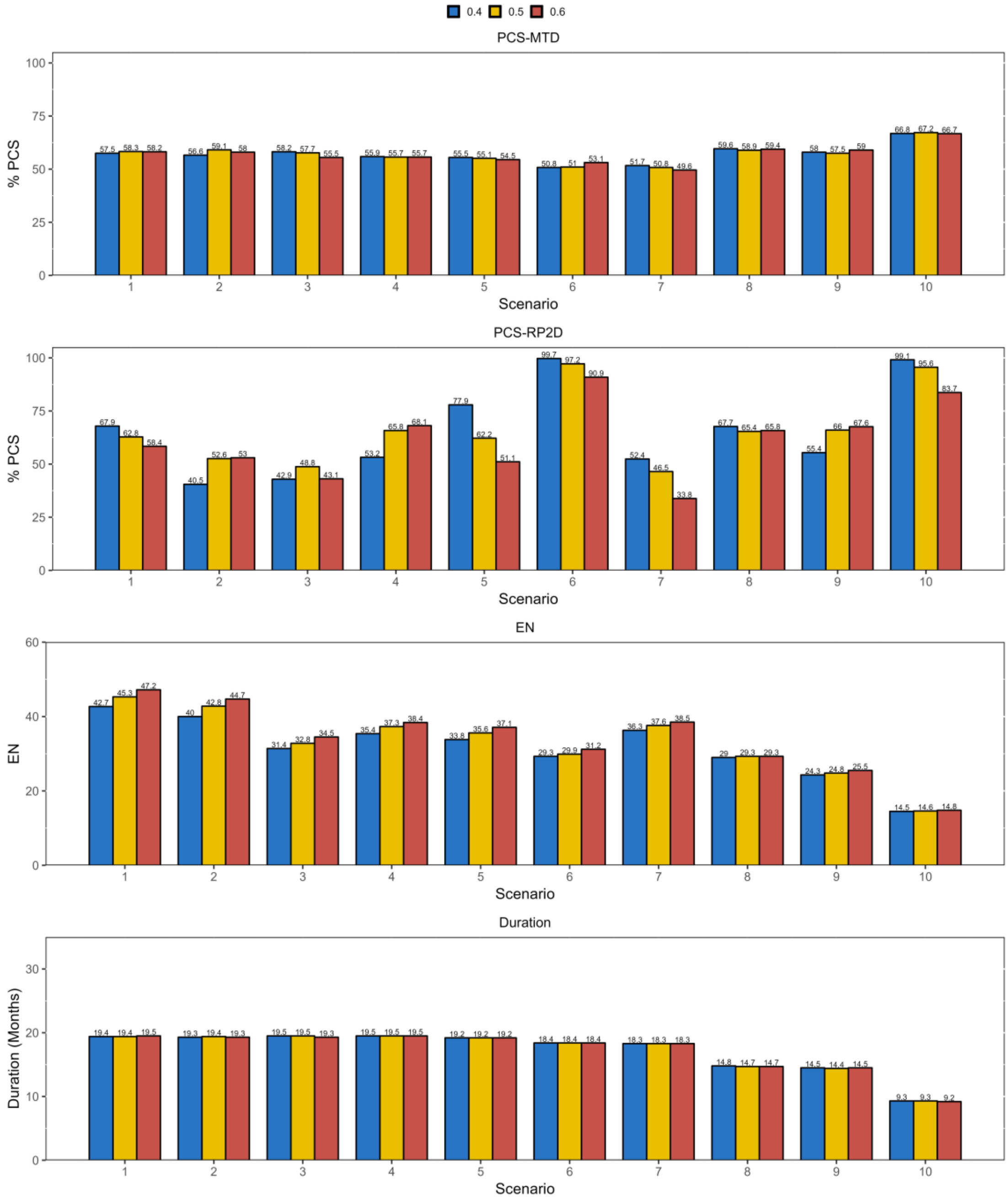

Figure S10 Comparison of three cutoff values for φ_Q on the percentage of correct selection, the expected sample size and the average trial duration.

Table S6 Detailed simulation results of sensitivity analysis 4B.

Cutoff φ_Q		Dose Level						EN	Duration (Month)
		1	2	3	4	5	6		
Scenario 1	DLT rate	0.01	0.03	0.06	0.1	0.12	0.25		
	Response rate	0.45	0.45	0.45	0.5	0.5	0.5		
	Mean change of QoL	0	-6	-10	-14	-18	-20		
0.4	Pts(%)	10.7(25%)	7.9(18.5%)	5.8(13.6%)	5.3(12.5%)	6.4(14.9%)	6.6(15.5%)	42.7	19.4
	Sel%-MTD	0	2	4.4	10.9	25.2	57.5		
	Sel%-RP2D	67.9	21.6	3.5	0.3	0	0		
0.5	Pts(%)	10.7(23.7%)	9.2(20.3%)	6.8(15.1%)	5.6(12.4%)	6.3(14%)	6.6(14.5%)	45.3	19.4
	Sel%-MTD	0	2	4.8	10.4	24.5	58.3		
	Sel%-RP2D	62.8	27	6.7	0.8	0	0		
0.6	Pts(%)	10.6(22.5%)	9.5(20.2%)	8(16.8%)	6.2(13.1%)	6.4(13.6%)	6.5(13.8%)	47.2	19.5
	Sel%-MTD	0	1.9	5	10.6	24.3	58.2		
	Sel%-RP2D	58.4	30.4	9	1.4	0	0		
Scenario 2	DLT rate	0.01	0.03	0.06	0.1	0.12	0.25		
	Response rate	0.2	0.45	0.45	0.5	0.5	0.5		
	Mean change of QoL	0	-6	-10	-14	-18	-20		
0.4	Pts(%)	7.8(19.5%)	8.1(20.2%)	5.9(14.9%)	5.3(13.4%)	6.3(15.8%)	6.5(16.3%)	40	19.3
	Sel%-MTD	0	1.8	5.4	11.1	25.1	56.6		
	Sel%-RP2D	27.7	40.5	5.3	0.2	0	0		
0.5	Pts(%)	7.8(18.2%)	9.4(22%)	7.1(16.6%)	5.6(13.1%)	6.3(14.7%)	6.6(15.4%)	42.8	19.4
	Sel%-MTD	0	1.8	5.1	10.4	23.6	59.1		
	Sel%-RP2D	22	52.6	13.2	1.6	0	0.1		
0.6	Pts(%)	7.7(17.3%)	9.9(22.1%)	8.2(18.3%)	6.1(13.7%)	6.3(14%)	6.5(14.6%)	44.7	19.3
	Sel%-MTD	0	1.8	5.1	10.9	24.2	58		

	Sel%-RP2D	19.3	53	19.1	4	0.1	0.1		
Scenario 3	DLT rate	0.01	0.03	0.06	0.1	0.12	0.25		
	Response rate	0.1	0.2	0.2	0.3	0.4	0.6		
	Mean change of QoL	-20	-18	-14	-14	-10	-6		
0.4	Pts(%)	3.3(10.6%)	3.9(12.5%)	4.9(15.5%)	5.5(17.5%)	7.2(22.9%)	6.6(20.8%)	31.4	19.5
	Sel%-MTD	0	1	4.3	11.6	24.9	58.2		
	Sel%-RP2D	0	0	0.2	0	7	42.9		
0.5	Pts(%)	3.4(10.2%)	4(12.1%)	5.2(15.8%)	5.9(17.9%)	8(24.2%)	6.5(19.8%)	32.8	19.5
	Sel%-MTD	0	1	4.3	11.9	25.1	57.7		
	Sel%-RP2D	0	0	0.3	1	18.3	48.8		
0.6	Pts(%)	3.4(9.8%)	4.1(11.9%)	5.8(16.7%)	6.4(18.7%)	8.5(24.6%)	6.3(18.4%)	34.5	19.3
	Sel%-MTD	0	1	4.5	12.4	26.6	55.5		
	Sel%-RP2D	0	0	2.5	5.3	29.3	43.1		
Scenario 4	DLT rate	0.01	0.03	0.06	0.1	0.12	0.25		
	Response rate	0.1	0.2	0.2	0.6	0.45	0.45		
	Mean change of QoL	-20	-18	-14	-6	-8	-10		
0.4	Pts(%)	3.3(9.5%)	3.9(11.1%)	4.9(13.9%)	9(25.5%)	7.8(22.1%)	6.3(17.8%)	35.4	19.5
	Sel%-MTD	0	1	4.3	11.4	27.4	55.9		
	Sel%-RP2D	0	0	0.1	53.2	15.8	5.3		
0.5	Pts(%)	3.4(9%)	4(10.7%)	5.2(13.9%)	10.1(27.2%)	8.4(22.5%)	6.3(16.8%)	37.3	19.5
	Sel%-MTD	0	1	4.3	12.1	26.9	55.7		
	Sel%-RP2D	0	0	0.2	65.8	18	7.8		
0.6	Pts(%)	3.4(8.8%)	4.1(10.7%)	5.7(14.9%)	10.5(27.4%)	8.5(22.1%)	6.2(16%)	38.4	19.5
	Sel%-MTD	0	1	4.5	13	25.8	55.7		
	Sel%-RP2D	0	0	1.2	68.1	16.2	8.8		
Scenario 5	DLT rate	0.01	0.03	0.06	0.1	0.12	0.25		

	Response rate	0.1	0.15	0.2	0.4	0.4	0.4		
	Mean change of QoL	-4	-8	-12	-18	-20	-20		
0.4	Pts(%)	5.4(15.9%)	5.4(15.9%)	5.1(15%)	5.3(15.6%)	6.3(18.6%)	6.4(19%)	33.8	19.2
	Sel%-MTD	0	1.4	4.5	12.2	26.4	55.5		
	Sel%-RP2D	12.3	8.7	1.1	0	0	0		
0.5	Pts(%)	5.7(16%)	6.3(17.7%)	5.7(15.9%)	5.4(15.2%)	6.2(17.5%)	6.3(17.8%)	35.6	19.2
	Sel%-MTD	0	1.4	4.2	12.3	27	55.1		
	Sel%-RP2D	14.1	18.5	5.2	0	0	0		
0.6	Pts(%)	5.8(15.6%)	6.8(18.3%)	6.4(17.4%)	5.6(15%)	6.3(17.1%)	6.2(16.7%)	37.1	19.2
	Sel%-MTD	0	1.4	4.9	12.6	26.6	54.5		
	Sel%-RP2D	13.5	21.4	13.6	0.4	0	0		
Scenario 6	DLT rate	0.03	0.06	0.12	0.25	0.4	0.55		
	Response rate	0.2	0.2	0.3	0.4	0.4	0.4		
	Mean change of QoL	-14	-14	-18	-20	-20	-20		
0.4	Pts(%)	4(13.8%)	5.5(18.6%)	7.8(26.7%)	7.8(26.6%)	3.5(11.9%)	0.7(2.5%)	29.3	18.4
	Sel%-MTD	0.8	5.7	28.3	50.8	13.5	0.9		
	Sel%-RP2D	0.1	0.2	0	0	0	0		
0.5	Pts(%)	4.3(14.5%)	5.7(19%)	7.8(26%)	7.8(26.2%)	3.5(11.9%)	0.7(2.4%)	29.9	18.4
	Sel%-MTD	0.8	5.9	27.1	51	14.5	0.7		
	Sel%-RP2D	0.9	1.9	0	0	0	0		
0.6	Pts(%)	5(15.9%)	6.3(20.1%)	7.9(25.5%)	7.9(25.2%)	3.5(11.1%)	0.7(2.2%)	31.2	18.4
	Sel%-MTD	0.8	5.7	26.8	53.1	12.7	0.9		
	Sel%-RP2D	4	5	0.1	0	0	0		
Scenario 7	DLT rate	0.03	0.06	0.12	0.25	0.4	0.55		
	Response rate	0.1	0.2	0.4	0.45	0.5	0.5		
	Mean change of QoL	-2	-4	-10	-4	-14	-18		

0.4	Pts(%)	6.1(16.9%)	8.5(23.4%)	8.7(24.1%)	9.1(25.2%)	3.2(8.7%)	0.6(1.6%)	36.3	18.3
	Sel%-MTD	0.8	5.1	29.7	51.7	11.3	1.4		
	Sel%-RP2D	4.8	20.9	6.3	52.4	0	0		
0.5	Pts(%)	6.3(16.7%)	8.9(23.7%)	9.5(25.4%)	9.2(24.4%)	3.2(8.5%)	0.5(1.4%)	37.6	18.3
	Sel%-MTD	0.8	5.3	30.4	50.8	11.7	1		
	Sel%-RP2D	3.9	18.7	21.6	46.5	0.2	0		
0.6	Pts(%)	6.3(16.3%)	9(23.5%)	10.5(27.1%)	9(23.4%)	3.2(8.2%)	0.6(1.5%)	38.5	18.3
	Sel%-MTD	0.8	5.3	31.1	49.6	12	1.2		
	Sel%-RP2D	3.8	16.3	40.2	33.8	0.9	0		
Scenario 8	DLT rate	0.1	0.25	0.4	0.55	0.65	0.65		
	Response rate	0.45	0.45	0.45	0.5	0.5	0.5		
	Mean change of QoL	-2	-6	-12	-18	-20	-20		
0.4	Pts(%)	13.1(45.2%)	10.9(37.6%)	4.1(14.1%)	0.8(2.7%)	0.1(0.3%)	0(0%)	29	14.8
	Sel%-MTD	26	59.6	13.1	0.9	0	0		
	Sel%-RP2D	67.7	29.4	0.1	0	0	0		
0.5	Pts(%)	13.3(45.4%)	11.1(37.8%)	4(13.8%)	0.8(2.7%)	0.1(0.2%)	0(0%)	29.3	14.7
	Sel%-MTD	26.1	58.9	13.9	0.7	0	0		
	Sel%-RP2D	65.4	32.2	0.8	0	0	0		
0.6	Pts(%)	13.3(45.4%)	11.1(37.9%)	4.1(13.9%)	0.8(2.6%)	0.1(0.2%)	0(0%)	29.3	14.7
	Sel%-MTD	26.4	59.4	13.1	0.7	0	0		
	Sel%-RP2D	65.8	31.6	1.4	0	0	0		
Scenario 9	DLT rate	0.1	0.25	0.4	0.55	0.65	0.65		
	Response rate	0.2	0.45	0.45	0.5	0.5	0.5		
	Mean change of QoL	-14	-6	-12	-18	-20	-20		
0.4	Pts(%)	8.7(35.7%)	10.8(44.4%)	3.9(16.3%)	0.8(3.3%)	0.1(0.3%)	0(0%)	24.3	14.5
	Sel%-MTD	26.9	58	14	0.8	0	0		

	Sel%-RP2D	0.1	55.4	0.4	0	0	0		
0.5	Pts(%)	8.9(35.9%)	11.1(44.8%)	3.9(15.9%)	0.8(3.2%)	0.1(0.2%)	0(0%)	24.8	14.4
	Sel%-MTD	27.4	57.5	14	0.8	0	0		
	Sel%-RP2D	1.2	66	1.4	0	0	0		
0.6	Pts(%)	9.4(36.9%)	11.2(44.1%)	4(15.7%)	0.8(3.1%)	0.1(0.2%)	0(0%)	25.5	14.5
	Sel%-MTD	26.8	59	13.3	0.6	0	0		
	Sel%-RP2D	4.4	67.6	2.4	0	0	0		
Scenario 10	DLT rate	0.3	0.35	0.4	0.55	0.65	0.65		
	Response rate	0.3	0.3	0.3	0.3	0.3	0.3		
	Mean change of QoL	-14	-15	-16	-18	-20	-20		
0.4	Pts(%)	9.1(63.1%)	3.8(26.1%)	1.2(8.6%)	0.3(2%)	0(0.2%)	0(0%)	14.5	9.3
	Sel%-MTD	66.8	12.5	3.7	0.5	0	0		
	Sel%-RP2D	0.9	0	0	0	0	0		
0.5	Pts(%)	9.3(63.6%)	3.8(26.1%)	1.2(8.3%)	0.3(1.8%)	0(0.2%)	0(0%)	14.6	9.3
	Sel%-MTD	67.2	12.3	3.5	0.5	0	0		
	Sel%-RP2D	4.3	0	0.1	0	0	0		
0.6	Pts(%)	9.5(64.1%)	3.8(25.9%)	1.2(8.2%)	0.2(1.7%)	0(0.2%)	0(0%)	14.8	9.2
	Sel%-MTD	66.7	12.6	3.5	0.5	0	0		
	Sel%-RP2D	15	1	0.3	0	0	0		

Sensitivity analysis 4C

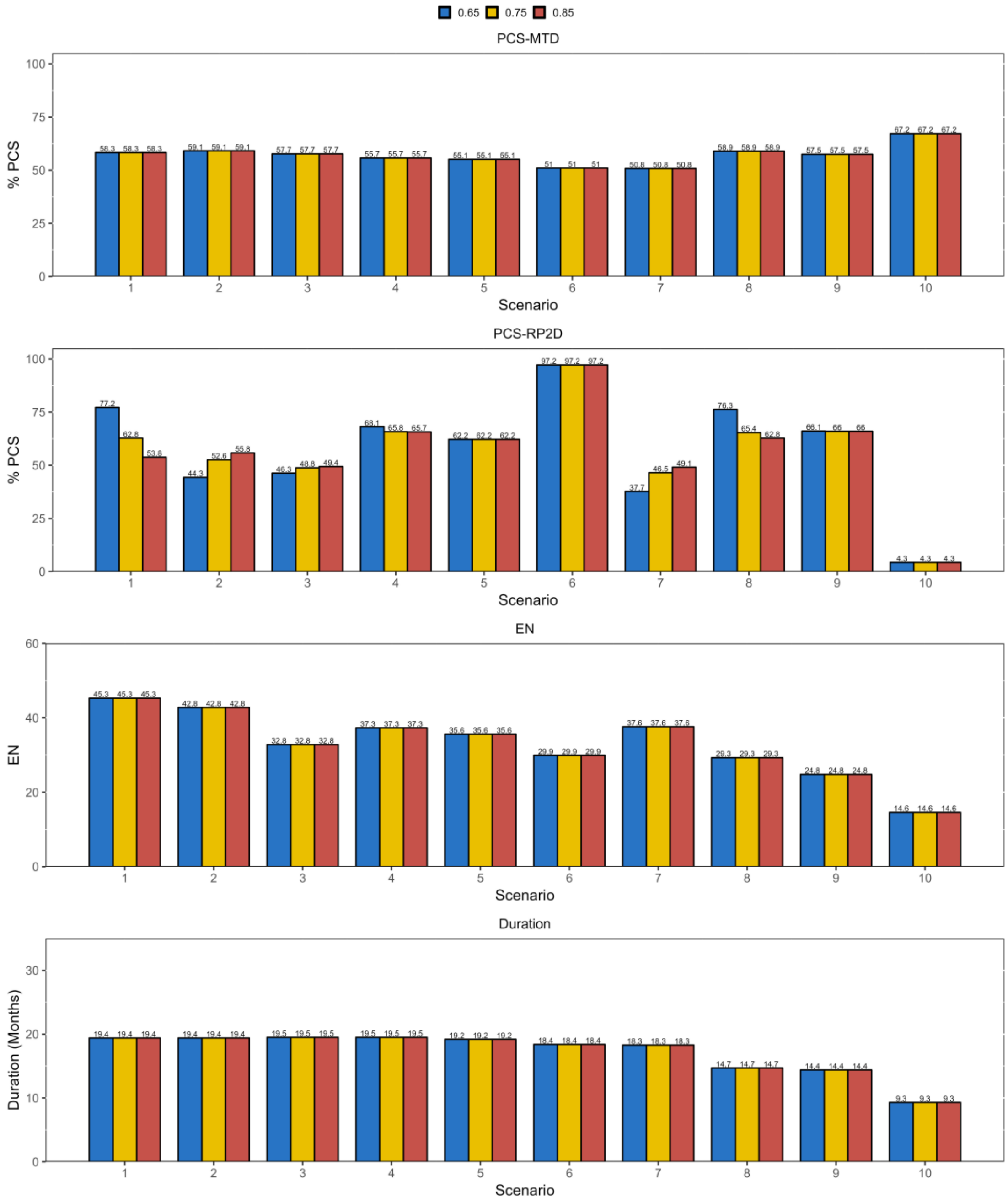

Figure S11 Comparison of three cutoff values for φ_C on the percentage of correct selection, the expected sample size and the average trial duration.

Table S7 Detailed simulation results of sensitivity analysis 4C.

Cutoff φ_C		Dose Level						EN	Duration (Month)
		1	2	3	4	5	6		
Scenario 1	DLT rate	0.01	0.03	0.06	0.1	0.12	0.25		
	Response rate	0.45	0.45	0.45	0.5	0.5	0.5		
	Mean change of QoL	0	-6	-10	-14	-18	-20		
0.65	Pts(%)	10.7(23.7%)	9.2(20.3%)	6.8(15.1%)	5.6(12.4%)	6.3(14%)	6.6(14.5%)	45.3	19.4
	Sel%-MTD	0	2	4.8	10.4	24.5	58.3		
	Sel%-RP2D	77.2	16.7	3.4	0	0	0		
0.75	Pts(%)	10.7(23.7%)	9.2(20.3%)	6.8(15.1%)	5.6(12.4%)	6.3(14%)	6.6(14.5%)	45.3	19.4
	Sel%-MTD	0	2	4.8	10.4	24.5	58.3		
	Sel%-RP2D	62.8	27	6.7	0.8	0	0		
0.85	Pts(%)	10.7(23.7%)	9.2(20.3%)	6.8(15.1%)	5.6(12.4%)	6.3(14%)	6.6(14.5%)	45.3	19.4
	Sel%-MTD	0	2	4.8	10.4	24.5	58.3		
	Sel%-RP2D	53.8	31.5	10.7	1.3	0	0		
Scenario 2	DLT rate	0.01	0.03	0.06	0.1	0.12	0.25		
	Response rate	0.2	0.45	0.45	0.5	0.5	0.5		
	Mean change of QoL	0	-6	-10	-14	-18	-20		
0.65	Pts(%)	7.8(18.2%)	9.4(22%)	7.1(16.6%)	5.6(13.1%)	6.3(14.7%)	6.6(15.4%)	42.8	19.4
	Sel%-MTD	0	1.8	5.1	10.4	23.6	59.1		
	Sel%-RP2D	34.1	44.3	9.9	1.1	0	0.1		
0.75	Pts(%)	7.8(18.2%)	9.4(22%)	7.1(16.6%)	5.6(13.1%)	6.3(14.7%)	6.6(15.4%)	42.8	19.4
	Sel%-MTD	0	1.8	5.1	10.4	23.6	59.1		
	Sel%-RP2D	22	52.6	13.2	1.6	0	0.1		
0.85	Pts(%)	7.8(18.2%)	9.4(22%)	7.1(16.6%)	5.6(13.1%)	6.3(14.7%)	6.6(15.4%)	42.8	19.4
	Sel%-MTD	0	1.8	5.1	10.4	23.6	59.1		

	Sel%-RP2D	14.7	55.8	17.1	1.8	0	0.1		
Scenario 3	DLT rate	0.01	0.03	0.06	0.1	0.12	0.25		
	Response rate	0.1	0.2	0.2	0.3	0.4	0.6		
	Mean change of QoL	-20	-18	-14	-14	-10	-6		
0.65	Pts(%)	3.4(10.2%)	4(12.1%)	5.2(15.8%)	5.9(17.9%)	8(24.2%)	6.5(19.8%)	32.8	19.5
	Sel%-MTD	0	1	4.3	11.9	25.1	57.7		
	Sel%-RP2D	0	0	0.3	1.1	20.7	46.3		
0.75	Pts(%)	3.4(10.2%)	4(12.1%)	5.2(15.8%)	5.9(17.9%)	8(24.2%)	6.5(19.8%)	32.8	19.5
	Sel%-MTD	0	1	4.3	11.9	25.1	57.7		
	Sel%-RP2D	0	0	0.3	1	18.3	48.8		
0.85	Pts(%)	3.4(10.2%)	4(12.1%)	5.2(15.8%)	5.9(17.9%)	8(24.2%)	6.5(19.8%)	32.8	19.5
	Sel%-MTD	0	1	4.3	11.9	25.1	57.7		
	Sel%-RP2D	0	0	0.3	1	17.7	49.4		
Scenario 4	DLT rate	0.01	0.03	0.06	0.1	0.12	0.25		
	Response rate	0.1	0.2	0.2	0.6	0.45	0.45		
	Mean change of QoL	-20	-18	-14	-6	-8	-10		
0.65	Pts(%)	3.4(9%)	4(10.7%)	5.2(13.9%)	10.1(27.2%)	8.4(22.5%)	6.3(16.8%)	37.3	19.5
	Sel%-MTD	0	1	4.3	12.1	26.9	55.7		
	Sel%-RP2D	0	0	0.4	68.1	16.4	6.9		
0.75	Pts(%)	3.4(9%)	4(10.7%)	5.2(13.9%)	10.1(27.2%)	8.4(22.5%)	6.3(16.8%)	37.3	19.5
	Sel%-MTD	0	1	4.3	12.1	26.9	55.7		
	Sel%-RP2D	0	0	0.2	65.8	18	7.8		
0.85	Pts(%)	3.4(9%)	4(10.7%)	5.2(13.9%)	10.1(27.2%)	8.4(22.5%)	6.3(16.8%)	37.3	19.5
	Sel%-MTD	0	1	4.3	12.1	26.9	55.7		
	Sel%-RP2D	0	0	0.2	65.7	18	7.9		
Scenario 5	DLT rate	0.01	0.03	0.06	0.1	0.12	0.25		

	Response rate	0.1	0.15	0.2	0.4	0.4	0.4		
	Mean change of QoL	-4	-8	-12	-18	-20	-20		
0.65	Pts(%)	5.7(16%)	6.3(17.7%)	5.7(15.9%)	5.4(15.2%)	6.2(17.5%)	6.3(17.8%)	35.6	19.2
	Sel%-MTD	0	1.4	4.2	12.3	27	55.1		
	Sel%-RP2D	14.7	18.2	4.9	0	0	0		
0.75	Pts(%)	5.7(16%)	6.3(17.7%)	5.7(15.9%)	5.4(15.2%)	6.2(17.5%)	6.3(17.8%)	35.6	19.2
	Sel%-MTD	0	1.4	4.2	12.3	27	55.1		
	Sel%-RP2D	14.1	18.5	5.2	0	0	0		
0.85	Pts(%)	5.7(16%)	6.3(17.7%)	5.7(15.9%)	5.4(15.2%)	6.2(17.5%)	6.3(17.8%)	35.6	19.2
	Sel%-MTD	0	1.4	4.2	12.3	27	55.1		
	Sel%-RP2D	14.1	18.5	5.2	0	0	0		
Scenario 6	DLT rate	0.03	0.06	0.12	0.25	0.4	0.55		
	Response rate	0.2	0.2	0.3	0.4	0.4	0.4		
	Mean change of QoL	-14	-14	-18	-20	-20	-20		
0.65	Pts(%)	4.3(14.5%)	5.7(19%)	7.8(26%)	7.8(26.2%)	3.5(11.9%)	0.7(2.4%)	29.9	18.4
	Sel%-MTD	0.8	5.9	27.1	51	14.5	0.7		
	Sel%-RP2D	0.9	1.9	0	0	0	0		
0.75	Pts(%)	4.3(14.5%)	5.7(19%)	7.8(26%)	7.8(26.2%)	3.5(11.9%)	0.7(2.4%)	29.9	18.4
	Sel%-MTD	0.8	5.9	27.1	51	14.5	0.7		
	Sel%-RP2D	0.9	1.9	0	0	0	0		
0.85	Pts(%)	4.3(14.5%)	5.7(19%)	7.8(26%)	7.8(26.2%)	3.5(11.9%)	0.7(2.4%)	29.9	18.4
	Sel%-MTD	0.8	5.9	27.1	51	14.5	0.7		
	Sel%-RP2D	0.9	1.9	0	0	0	0		
Scenario 7	DLT rate	0.03	0.06	0.12	0.25	0.4	0.55		
	Response rate	0.1	0.2	0.4	0.45	0.5	0.5		
	Mean change of QoL	-2	-4	-10	-4	-14	-18		

0.65	Pts(%)	6.3(16.7%)	8.9(23.7%)	9.5(25.4%)	9.2(24.4%)	3.2(8.5%)	0.5(1.4%)	37.6	18.3
	Sel%-MTD	0.8	5.3	30.4	50.8	11.7	1		
	Sel%-RP2D	7.5	24.1	21.6	37.7	0	0		
0.75	Pts(%)	6.3(16.7%)	8.9(23.7%)	9.5(25.4%)	9.2(24.4%)	3.2(8.5%)	0.5(1.4%)	37.6	18.3
	Sel%-MTD	0.8	5.3	30.4	50.8	11.7	1		
	Sel%-RP2D	3.9	18.7	21.6	46.5	0.2	0		
0.85	Pts(%)	6.3(16.7%)	8.9(23.7%)	9.5(25.4%)	9.2(24.4%)	3.2(8.5%)	0.5(1.4%)	37.6	18.3
	Sel%-MTD	0.8	5.3	30.4	50.8	11.7	1		
	Sel%-RP2D	3	16.9	21.7	49.1	0.2	0		
Scenario 8	DLT rate	0.1	0.25	0.4	0.55	0.65	0.65		
	Response rate	0.45	0.45	0.45	0.5	0.5	0.5		
	Mean change of QoL	-2	-6	-12	-18	-20	-20		
0.65	Pts(%)	13.3(45.4%)	11.1(37.8%)	4(13.8%)	0.8(2.7%)	0.1(0.2%)	0(0%)	29.3	14.7
	Sel%-MTD	26.1	58.9	13.9	0.7	0	0		
	Sel%-RP2D	76.3	21.9	0.2	0	0	0		
0.75	Pts(%)	13.3(45.4%)	11.1(37.8%)	4(13.8%)	0.8(2.7%)	0.1(0.2%)	0(0%)	29.3	14.7
	Sel%-MTD	26.1	58.9	13.9	0.7	0	0		
	Sel%-RP2D	65.4	32.2	0.8	0	0	0		
0.85	Pts(%)	13.3(45.4%)	11.1(37.8%)	4(13.8%)	0.8(2.7%)	0.1(0.2%)	0(0%)	29.3	14.7
	Sel%-MTD	26.1	58.9	13.9	0.7	0	0		
	Sel%-RP2D	62.8	34.6	1	0	0	0		
Scenario 9	DLT rate	0.1	0.25	0.4	0.55	0.65	0.65		
	Response rate	0.2	0.45	0.45	0.5	0.5	0.5		
	Mean change of QoL	-14	-6	-12	-18	-20	-20		
0.65	Pts(%)	8.9(35.9%)	11.1(44.8%)	3.9(15.9%)	0.8(3.2%)	0.1(0.2%)	0(0%)	24.8	14.4
	Sel%-MTD	27.4	57.5	14	0.8	0	0		

	Sel%-RP2D	1.2	66.1	1.3	0	0	0		
0.75	Pts(%)	8.9(35.9%)	11.1(44.8%)	3.9(15.9%)	0.8(3.2%)	0.1(0.2%)	0(0%)	24.8	14.4
	Sel%-MTD	27.4	57.5	14	0.8	0	0		
	Sel%-RP2D	1.2	66	1.4	0	0	0		
0.85	Pts(%)	8.9(35.9%)	11.1(44.8%)	3.9(15.9%)	0.8(3.2%)	0.1(0.2%)	0(0%)	24.8	14.4
	Sel%-MTD	27.4	57.5	14	0.8	0	0		
	Sel%-RP2D	1.2	66	1.4	0	0	0		
Scenario 10	DLT rate	0.3	0.35	0.4	0.55	0.65	0.65		
	Response rate	0.3	0.3	0.3	0.3	0.3	0.3		
	Mean change of QoL	-14	-15	-16	-18	-20	-20		
0.65	Pts(%)	9.3(63.6%)	3.8(26.1%)	1.2(8.3%)	0.3(1.8%)	0(0.2%)	0(0%)	14.6	9.3
	Sel%-MTD	67.2	12.3	3.5	0.5	0	0		
	Sel%-RP2D	4.3	0	0.1	0	0	0		
0.75	Pts(%)	9.3(63.6%)	3.8(26.1%)	1.2(8.3%)	0.3(1.8%)	0(0.2%)	0(0%)	14.6	9.3
	Sel%-MTD	67.2	12.3	3.5	0.5	0	0		
	Sel%-RP2D	4.3	0	0.1	0	0	0		
0.85	Pts(%)	9.3(63.6%)	3.8(26.1%)	1.2(8.3%)	0.3(1.8%)	0(0.2%)	0(0%)	14.6	9.3
	Sel%-MTD	67.2	12.3	3.5	0.5	0	0		
	Sel%-RP2D	4.3	0	0.1	0	0	0		

Sensitivity analysis 5

Let x_{ji} and r_{ji} denote the binary DLT and response outcome for the i_{th} patient treated with dose level j. L_{ji} and M_{ji} denote the corresponding latent variables, where $x_{ji} = I(L_{ji} > 0)$ and $r_{ji} = I(M_{ji} > 0)$. Trivariate normal distributions are used to simultaneously simulate QoL outcomes and two latent variables as follows

$$\begin{pmatrix} L_{ji} \\ M_{ji} \\ y_{ji} \end{pmatrix} \sim MVN\left(\begin{pmatrix} \mu_{Lj} \\ \mu_{Mj} \\ \mu_j \end{pmatrix}, \begin{pmatrix} 1 & \rho_1 & \rho_2\sigma_j \\ \rho_1 & 1 & \rho_3\sigma_j \\ \rho_2\sigma_j & \rho_3\sigma_j & \sigma_j^2 \end{pmatrix} \right).$$

μ_{Lj} and μ_{Mj} are determined by letting $\Pr(L_{ji} > 0) = p_j$ and $\Pr(M_{ji} > 0) = \theta_j$, thus the marginal distributions of the three outcomes are still the same as those in the manuscript body. (ρ_1, ρ_2, ρ_3) indicates the vector of correlation parameters.

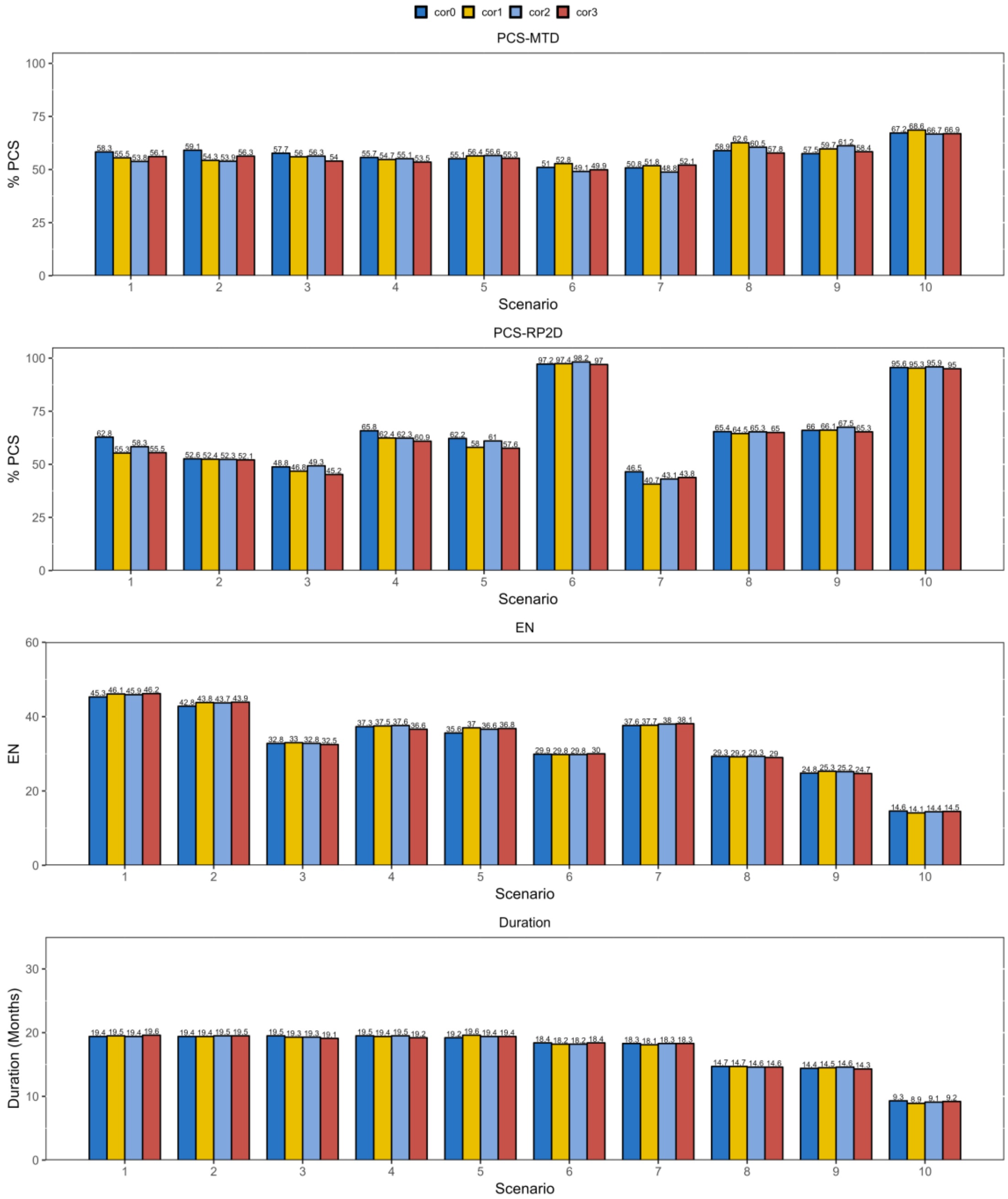

Figure S12 Comparison of four correlation settings on the percentage of correct selection, the expected sample size and the average trial duration. The labels cor0, cor1, cor2 and cor3 correspond to the cases where clinical outcomes are simulated independently, $(\rho_1, \rho_2, \rho_3) = (0.5, -0.5, 0.5)$, $(\rho_1, \rho_2, \rho_3) = (0.25, -0.75, 0.25)$, and $(\rho_1, \rho_2, \rho_3) = (0.5, -0.25, 0.5)$, respectively.

Table S8 Detailed simulation results of sensitivity analysis 5.

Correlation		Dose Level						EN	Duration (Month)
		1	2	3	4	5	6		
Scenario 1	DLT rate	0.01	0.03	0.06	0.1	0.12	0.25		
	Response rate	0.45	0.45	0.45	0.5	0.5	0.5		
	Mean change of QoL	0	-6	-10	-14	-18	-20		
cor0	Pts(%)	10.7(23.7%)	9.2(20.3%)	6.8(15.1%)	5.6(12.4%)	6.3(14%)	6.6(14.5%)	45.3	19.4
	Sel%-MTD	0	2	4.8	10.4	24.5	58.3		
	Sel%-RP2D	62.8	27	6.7	0.8	0	0		
cor1	Pts(%)	10.5(22.9%)	9.6(20.8%)	7.2(15.7%)	5.9(12.9%)	6.5(14.1%)	6.3(13.7%)	46.1	19.5
	Sel%-MTD	0.1	1.1	5	12.4	25.9	55.5		
	Sel%-RP2D	55.3	30.6	10.8	0.8	0	0		
cor2	Pts(%)	10.6(23.2%)	9.5(20.8%)	7.2(15.8%)	5.7(12.4%)	6.4(14%)	6.3(13.8%)	45.9	19.4
	Sel%-MTD	0	1.6	5.4	12	27.2	53.8		
	Sel%-RP2D	58.3	30.2	8.4	0.9	0	0		
cor3	Pts(%)	10.5(22.7%)	9.6(20.7%)	7.2(15.5%)	6(13%)	6.6(14.2%)	6.4(13.9%)	46.2	19.6
	Sel%-MTD	0	0.9	4.2	12.1	26.7	56.1		
	Sel%-RP2D	55.5	32.7	8.7	0.8	0	0		
Scenario 2	DLT rate	0.01	0.03	0.06	0.1	0.12	0.25		
	Response rate	0.2	0.45	0.45	0.5	0.5	0.5		
	Mean change of QoL	0	-6	-10	-14	-18	-20		
cor0	Pts(%)	7.8(18.2%)	9.4(22%)	7.1(16.6%)	5.6(13.1%)	6.3(14.7%)	6.6(15.4%)	42.8	19.4
	Sel%-MTD	0	1.8	5.1	10.4	23.6	59.1		
	Sel%-RP2D	22	52.6	13.2	1.6	0	0.1		
cor1	Pts(%)	7.9(17.9%)	9.9(22.5%)	7.4(16.9%)	5.9(13.5%)	6.5(14.8%)	6.3(14.4%)	43.8	19.4
	Sel%-MTD	0.1	0.8	5.4	11.8	27.6	54.3		

	Sel%-RP2D	22	52.4	14.7	1.7	0	0		
cor2	Pts(%)	8(18.2%)	9.8(22.4%)	7.3(16.8%)	5.8(13.1%)	6.5(14.8%)	6.4(14.7%)	43.7	19.5
	Sel%-MTD	0	1.6	5.8	11.3	27.4	53.9		
	Sel%-RP2D	22.7	52.3	13.9	1.4	0	0		
cor3	Pts(%)	7.8(17.8%)	9.8(22.2%)	7.3(16.6%)	6(13.7%)	6.6(15%)	6.4(14.6%)	43.9	19.5
	Sel%-MTD	0	0.9	4.2	12.2	26.4	56.3		
	Sel%-RP2D	22.3	52.1	15	1.3	0	0		
Scenario 3	DLT rate	0.01	0.03	0.06	0.1	0.12	0.25		
	Response rate	0.1	0.2	0.2	0.3	0.4	0.6		
	Mean change of QoL	-20	-18	-14	-14	-10	-6		
cor0	Pts(%)	3.4(10.2%)	4(12.1%)	5.2(15.8%)	5.9(17.9%)	8(24.2%)	6.5(19.8%)	32.8	19.5
	Sel%-MTD	0	1	4.3	11.9	25.1	57.7		
	Sel%-RP2D	0	0	0.3	1	18.3	48.8		
cor1	Pts(%)	3.4(10.3%)	4(12.1%)	5.5(16.7%)	6(18%)	7.9(24%)	6.2(18.9%)	33	19.3
	Sel%-MTD	0.1	1	5.5	12.9	24.5	56		
	Sel%-RP2D	0	0	0.9	0.7	15.2	46.8		
cor2	Pts(%)	3.4(10.3%)	4(12.2%)	5.5(16.7%)	5.8(17.7%)	7.9(24%)	6.3(19.1%)	32.8	19.3
	Sel%-MTD	0	0.8	6.6	11.6	24.7	56.3		
	Sel%-RP2D	0	0	0.5	0.9	15	49.3		
cor3	Pts(%)	3.4(10.4%)	4(12.2%)	5.5(16.9%)	6(18.5%)	7.6(23.5%)	6(18.5%)	32.5	19.1
	Sel%-MTD	0	1.4	5.2	15.2	24.2	54		
	Sel%-RP2D	0	0	1.1	0.5	17.1	45.2		
Scenario 4	DLT rate	0.01	0.03	0.06	0.1	0.12	0.25		
	Response rate	0.1	0.2	0.2	0.6	0.45	0.45		
	Mean change of QoL	-20	-18	-14	-6	-8	-10		
cor0	Pts(%)	3.4(9%)	4(10.7%)	5.2(13.9%)	10.1(27.2%)	8.4(22.5%)	6.3(16.8%)	37.3	19.5

	Sel%-MTD	0	1	4.3	12.1	26.9	55.7		
	Sel%-RP2D	0	0	0.2	65.8	18	7.8		
cor1	Pts(%)	3.4(9.1%)	4(10.7%)	5.6(14.9%)	10.3(27.4%)	8.1(21.7%)	6.1(16.3%)	37.5	19.4
	Sel%-MTD	0.1	1	5.6	12.7	25.9	54.7		
	Sel%-RP2D	0	0	0.7	62.4	19.2	7.9		
cor2	Pts(%)	3.4(9%)	4(10.6%)	5.5(14.6%)	10.1(26.9%)	8.3(22.1%)	6.3(16.8%)	37.6	19.5
	Sel%-MTD	0	0.8	6.4	11.6	26.1	55.1		
	Sel%-RP2D	0	0	0.1	62.3	18.9	9		
cor3	Pts(%)	3.4(9.2%)	4(10.8%)	5.5(15.1%)	10(27.3%)	7.8(21.2%)	6(16.3%)	36.6	19.2
	Sel%-MTD	0	1.4	5.2	14.8	25.1	53.5		
	Sel%-RP2D	0	0	0.2	60.9	19.9	9		
Scenario 5	DLT rate	0.01	0.03	0.06	0.1	0.12	0.25		
	Response rate	0.1	0.15	0.2	0.4	0.4	0.4		
	Mean change of QoL	-4	-8	-12	-18	-20	-20		
cor0	Pts(%)	5.7(16%)	6.3(17.7%)	5.7(15.9%)	5.4(15.2%)	6.2(17.5%)	6.3(17.8%)	35.6	19.2
	Sel%-MTD	0	1.4	4.2	12.3	27	55.1		
	Sel%-RP2D	14.1	18.5	5.2	0	0	0		
cor1	Pts(%)	5.9(15.9%)	6.7(18.1%)	6(16.2%)	5.4(14.6%)	6.5(17.6%)	6.5(17.6%)	37	19.6
	Sel%-MTD	0.1	0.8	5.3	11.4	26	56.4		
	Sel%-RP2D	13.9	21.1	7	0	0	0		
cor2	Pts(%)	6(16.4%)	6.5(17.8%)	5.9(16.1%)	5.2(14.3%)	6.4(17.5%)	6.5(17.9%)	36.6	19.4
	Sel%-MTD	0	1.2	6.1	9.3	26.8	56.6		
	Sel%-RP2D	14.7	18.5	5.7	0	0.1	0		
cor3	Pts(%)	6(16.3%)	6.6(17.8%)	5.9(15.9%)	5.4(14.6%)	6.5(17.6%)	6.6(17.8%)	36.8	19.4
	Sel%-MTD	0	1	4.6	12.3	26.8	55.3		
	Sel%-RP2D	15	21.1	6.2	0	0.1	0		

Scenario 6	DLT rate	0.03	0.06	0.12	0.25	0.4	0.55		
	Response rate	0.2	0.2	0.3	0.4	0.4	0.4		
	Mean change of QoL	-14	-14	-18	-20	-20	-20		
cor0	Pts(%)	4.3(14.5%)	5.7(19%)	7.8(26%)	7.8(26.2%)	3.5(11.9%)	0.7(2.4%)	29.9	18.4
	Sel%-MTD	0.8	5.9	27.1	51	14.5	0.7		
	Sel%-RP2D	0.9	1.9	0	0	0	0		
cor1	Pts(%)	4.6(15.4%)	5.4(18.3%)	7.7(25.7%)	8(26.8%)	3.4(11.5%)	0.7(2.2%)	29.8	18.2
	Sel%-MTD	2	5.2	27.2	52.8	12.5	0.3		
	Sel%-RP2D	2	0.6	0	0	0	0		
cor2	Pts(%)	4.6(15.3%)	5.8(19.4%)	8(26.8%)	7.7(25.7%)	3.2(10.6%)	0.7(2.2%)	29.8	18.2
	Sel%-MTD	1	6.1	30.8	49.1	12	1		
	Sel%-RP2D	1.3	0.5	0	0	0	0		
cor3	Pts(%)	4.5(15.1%)	5.6(18.8%)	7.8(26%)	7.9(26.2%)	3.4(11.4%)	0.7(2.5%)	30	18.4
	Sel%-MTD	1.1	5.2	30.1	49.9	12.6	1.1		
	Sel%-RP2D	1.9	1.1	0	0	0	0		
Scenario 7	DLT rate	0.03	0.06	0.12	0.25	0.4	0.55		
	Response rate	0.1	0.2	0.4	0.45	0.5	0.5		
	Mean change of QoL	-2	-4	-10	-4	-14	-18		
cor0	Pts(%)	6.3(16.7%)	8.9(23.7%)	9.5(25.4%)	9.2(24.4%)	3.2(8.5%)	0.5(1.4%)	37.6	18.3
	Sel%-MTD	0.8	5.3	30.4	50.8	11.7	1		
	Sel%-RP2D	3.9	18.7	21.6	46.5	0.2	0		
cor1	Pts(%)	6.4(17%)	8.6(22.9%)	9.7(25.8%)	9.1(24.2%)	3.2(8.4%)	0.6(1.7%)	37.7	18.1
	Sel%-MTD	1.9	5.6	28.6	51.8	11.2	0.9		
	Sel%-RP2D	5.8	18.9	24.4	40.7	0.7	0.1		
cor2	Pts(%)	6.6(17.3%)	8.8(23.1%)	9.7(25.6%)	9(23.7%)	3.2(8.5%)	0.6(1.7%)	38	18.3
	Sel%-MTD	0.9	6.7	29.9	48.8	12.2	1.5		

	Sel%-RP2D	5.6	19.2	21.1	43.1	0.8	0		
cor3	Pts(%)	6.5(17%)	8.7(22.7%)	9.9(25.9%)	9.3(24.4%)	3.2(8.4%)	0.6(1.6%)	38.1	18.3
	Sel%-MTD	1.1	4.8	29.4	52.1	11.7	0.9		
	Sel%-RP2D	5.4	18.5	22.7	43.8	0.7	0.1		
Scenario 8	DLT rate	0.1	0.25	0.4	0.55	0.65	0.65		
	Response rate	0.45	0.45	0.45	0.5	0.5	0.5		
	Mean change of QoL	-2	-6	-12	-18	-20	-20		
cor0	Pts(%)	13.3(45.4%)	11.1(37.8%)	4(13.8%)	0.8(2.7%)	0.1(0.2%)	0(0%)	29.3	14.7
	Sel%-MTD	26.1	58.9	13.9	0.7	0	0		
	Sel%-RP2D	65.4	32.2	0.8	0	0	0		
cor1	Pts(%)	13.2(45.3%)	11.2(38.4%)	4(13.8%)	0.7(2.3%)	0.1(0.2%)	0(0%)	29.2	14.7
	Sel%-MTD	24.7	62.6	11.1	1	0.1	0		
	Sel%-RP2D	64.5	33.3	1.2	0	0	0		
cor2	Pts(%)	13.3(45.4%)	11.2(38.1%)	4.1(13.8%)	0.7(2.4%)	0.1(0.2%)	0(0%)	29.3	14.6
	Sel%-MTD	25.4	60.5	12.7	0.5	0	0		
	Sel%-RP2D	65.3	31.2	1.6	0	0	0		
cor3	Pts(%)	13.3(45.7%)	10.9(37.7%)	4(13.7%)	0.8(2.6%)	0.1(0.2%)	0(0%)	29	14.6
	Sel%-MTD	27.3	57.8	13	1.1	0	0		
	Sel%-RP2D	65	32.7	1.2	0	0	0		
Scenario 9	DLT rate	0.1	0.25	0.4	0.55	0.65	0.65		
	Response rate	0.2	0.45	0.45	0.5	0.5	0.5		
	Mean change of QoL	-14	-6	-12	-18	-20	-20		
cor0	Pts(%)	8.9(35.9%)	11.1(44.8%)	3.9(15.9%)	0.8(3.2%)	0.1(0.2%)	0(0%)	24.8	14.4
	Sel%-MTD	27.4	57.5	14	0.8	0	0		
	Sel%-RP2D	1.2	66	1.4	0	0	0		
cor1	Pts(%)	9.2(36.5%)	11.4(45.1%)	3.9(15.6%)	0.7(2.6%)	0(0.1%)	0(0%)	25.3	14.5

	Sel%-MTD	26.6	59.7	12.6	0.6	0	0		
	Sel%-RP2D	1	66.1	2.6	0.1	0	0		
cor2	Pts(%)	9.4(37.2%)	11.5(45.4%)	3.8(15%)	0.6(2.4%)	0(0.1%)	0(0%)	25.2	14.6
	Sel%-MTD	25.8	61.2	11.7	0.6	0	0		
	Sel%-RP2D	0.4	67.5	2.7	0.1	0	0		
cor3	Pts(%)	9.2(37.1%)	11.1(44.7%)	3.8(15.3%)	0.6(2.6%)	0.1(0.2%)	0(0%)	24.7	14.3
	Sel%-MTD	27.1	58.4	12.8	0.8	0.1	0		
	Sel%-RP2D	0.8	65.3	2	0	0	0		
Scenario 10	DLT rate	0.3	0.35	0.4	0.55	0.65	0.65		
	Response rate	0.3	0.3	0.3	0.3	0.3	0.3		
	Mean change of QoL	-14	-15	-16	-18	-20	-20		
cor0	Pts(%)	9.3(63.6%)	3.8(26.1%)	1.2(8.3%)	0.3(1.8%)	0(0.2%)	0(0%)	14.6	9.3
	Sel%-MTD	67.2	12.3	3.5	0.5	0	0		
	Sel%-RP2D	4.3	0	0.1	0	0	0		
cor1	Pts(%)	9.2(65.5%)	3.5(24.5%)	1.1(8%)	0.2(1.7%)	0(0.2%)	0(0%)	14.1	8.9
	Sel%-MTD	68.6	11	3.2	0.1	0	0		
	Sel%-RP2D	4.1	0.5	0.1	0	0	0		
cor2	Pts(%)	9.3(64.8%)	3.6(25.3%)	1.2(8.3%)	0.2(1.5%)	0(0.1%)	0(0%)	14.4	9.1
	Sel%-MTD	66.7	12.6	2.7	0.1	0	0		
	Sel%-RP2D	4.1	0	0	0	0	0		
cor3	Pts(%)	9.2(63.9%)	3.7(25.5%)	1.2(8.4%)	0.3(2%)	0(0.2%)	0(0%)	14.5	9.2
	Sel%-MTD	66.9	11.3	3.5	0.5	0	0		
	Sel%-RP2D	4.7	0.2	0.1	0	0	0		

The labels cor0, cor1, cor2 and cor3 correspond to the cases where clinical outcomes are simulated independently, $(\rho_1, \rho_2, \rho_3) = (0.5, -0.5, 0.5)$, $(\rho_1, \rho_2, \rho_3) = (0.25, -0.75, 0.25)$, and $(\rho_1, \rho_2, \rho_3) = (0.5, -0.25, 0.5)$, respectively.

Sensitivity analysis 6

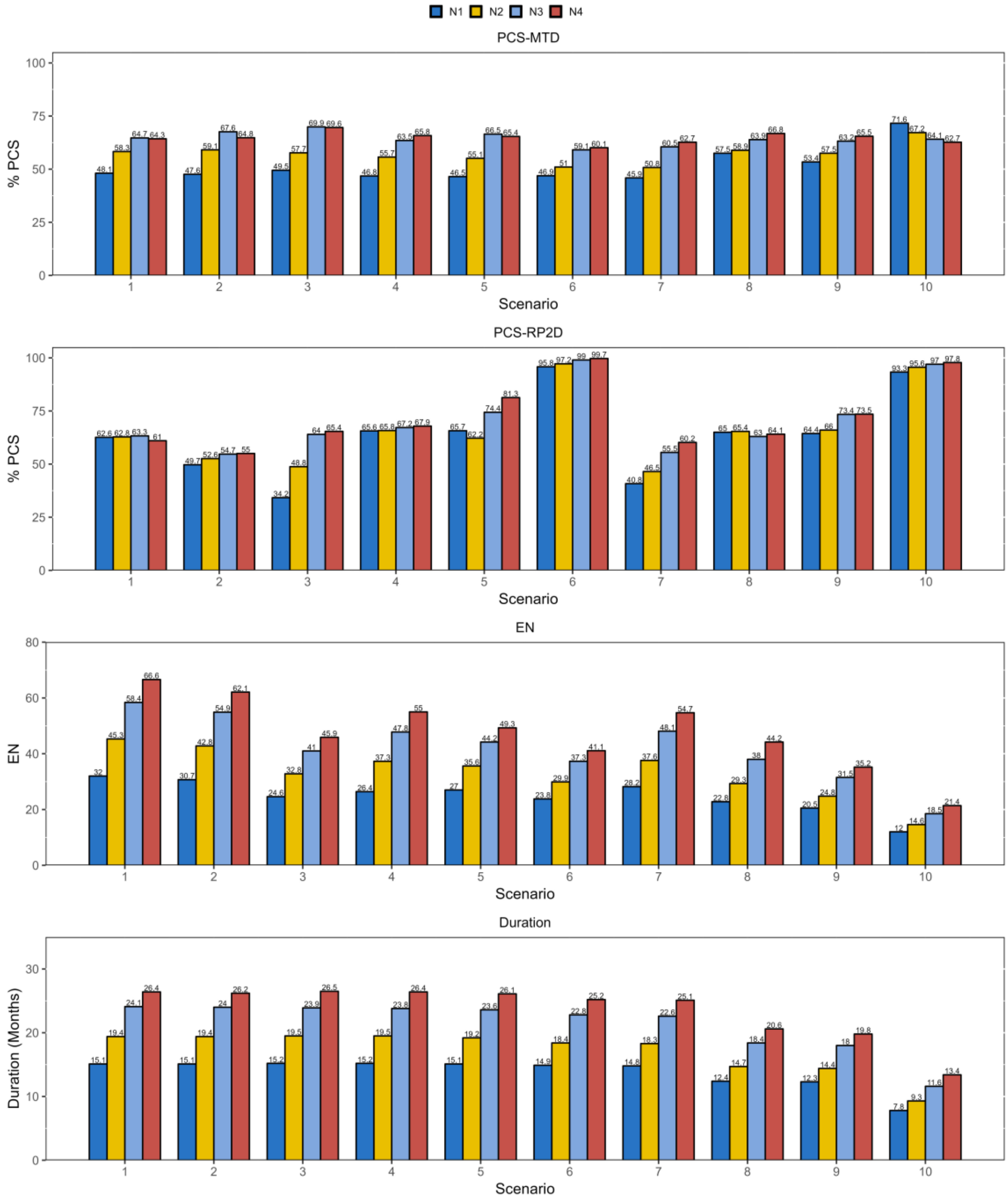

Figure S13 Comparison of four settings of sample size on the percentage of correct selection, the expected sample size and the average trial duration. The labels N1, N2, N3 and N4 correspond to the cases where $(N_{esc}, n_{stop}, n_{cap}) = (24,6,8)$, $(36,9,12)$, $(48,12,16)$, and $(60,15,20)$, respectively.

Table S9 Detailed simulation results of sensitivity analysis 6.

Sample size		Dose Level						EN	Duration (Month)
		1	2	3	4	5	6		
Scenario 1	DLT rate	0.01	0.03	0.06	0.1	0.12	0.25		
	Response rate	0.45	0.45	0.45	0.5	0.5	0.5		
	Mean change of QoL	0	-6	-10	-14	-18	-20		
N1	Pts(%)	7.5(23.3%)	6.9(21.5%)	5.9(18.4%)	5(15.7%)	3.6(11.4%)	3.1(9.7%)	32	15.1
	Sel%-MTD	0	2.3	5.5	16	28.1	48.1		
	Sel%-RP2D	62.6	25.8	8	1.3	0	0		
N2	Pts(%)	10.7(23.7%)	9.2(20.3%)	6.8(15.1%)	5.6(12.4%)	6.3(14%)	6.6(14.5%)	45.3	19.4
	Sel%-MTD	0	2	4.8	10.4	24.5	58.3		
	Sel%-RP2D	62.8	27	6.7	0.8	0	0		
N3	Pts(%)	13.8(23.6%)	11.3(19.4%)	8(13.6%)	6.4(10.9%)	9(15.4%)	10(17%)	58.4	24.1
	Sel%-MTD	0	0.5	1.8	6.8	26.2	64.7		
	Sel%-RP2D	63.3	27.8	5.3	0.5	0	0		
N4	Pts(%)	16.4(24.7%)	13(19.5%)	8.3(12.4%)	6.6(9.9%)	10.2(15.3%)	12.2(18.3%)	66.6	26.4
	Sel%-MTD	0	0.6	1.3	7.5	26.3	64.3		
	Sel%-RP2D	61	30.2	5.1	0.5	0	0		
Scenario 2	DLT rate	0.01	0.03	0.06	0.1	0.12	0.25		
	Response rate	0.2	0.45	0.45	0.5	0.5	0.5		
	Mean change of QoL	0	-6	-10	-14	-18	-20		
N1	Pts(%)	6(19.7%)	7(22.6%)	5.9(19.3%)	5(16.3%)	3.7(11.9%)	3.1(10.2%)	30.7	15.1
	Sel%-MTD	0	2.1	5	15.9	29.4	47.6		
	Sel%-RP2D	20.9	49.7	16	2.9	0.3	0		
N2	Pts(%)	7.8(18.2%)	9.4(22%)	7.1(16.6%)	5.6(13.1%)	6.3(14.7%)	6.6(15.4%)	42.8	19.4
	Sel%-MTD	0	1.8	5.1	10.4	23.6	59.1		

	Sel%-RP2D	22	52.6	13.2	1.6	0	0.1		
N3	Pts(%)	9.2(16.8%)	11.8(21.5%)	8.3(15.1%)	6.4(11.7%)	9(16.4%)	10.2(18.5%)	54.9	24
	Sel%-MTD	0	0.7	1.6	6.5	23.6	67.6		
	Sel%-RP2D	18.1	54.7	13.2	0.9	0	0		
N4	Pts(%)	10.4(16.8%)	14(22.5%)	8.8(14.1%)	6.5(10.5%)	10.1(16.3%)	12.3(19.8%)	62.1	26.2
	Sel%-MTD	0	0.6	1.8	7	25.8	64.8		
	Sel%-RP2D	16.2	55	13.1	0.8	0	0		
Scenario 3	DLT rate	0.01	0.03	0.06	0.1	0.12	0.25		
	Response rate	0.1	0.2	0.2	0.3	0.4	0.6		
	Mean change of QoL	-20	-18	-14	-14	-10	-6		
N1	Pts(%)	3.4(13.7%)	3.9(16%)	4.8(19.6%)	5.1(20.8%)	4.3(17.3%)	3.1(12.6%)	24.6	15.2
	Sel%-MTD	0	1.2	4.7	16.7	27.9	49.5		
	Sel%-RP2D	0	0	1.1	2.5	20.9	34.2		
N2	Pts(%)	3.4(10.2%)	4(12.1%)	5.2(15.8%)	5.9(17.9%)	8(24.2%)	6.5(19.8%)	32.8	19.5
	Sel%-MTD	0	1	4.3	11.9	25.1	57.7		
	Sel%-RP2D	0	0	0.3	1	18.3	48.8		
N3	Pts(%)	3.4(8.2%)	4(9.8%)	5.4(13.2%)	6.6(16.1%)	11.4(27.8%)	10.2(24.9%)	41	23.9
	Sel%-MTD	0	0.3	1.7	7.1	21	69.9		
	Sel%-RP2D	0	0	0.2	0.5	14.3	64		
N4	Pts(%)	3.4(7.3%)	4(8.8%)	5.5(11.9%)	6.9(15%)	13.4(29.2%)	12.8(27.8%)	45.9	26.5
	Sel%-MTD	0	0.3	1.5	6.9	21.7	69.6		
	Sel%-RP2D	0	0	0.1	0.4	12.6	65.4		
Scenario 4	DLT rate	0.01	0.03	0.06	0.1	0.12	0.25		
	Response rate	0.1	0.2	0.2	0.6	0.45	0.45		
	Mean change of QoL	-20	-18	-14	-6	-8	-10		
N1	Pts(%)	3.4(12.7%)	3.9(15%)	4.8(18.3%)	7(26.4%)	4.3(16.3%)	3(11.4%)	26.4	15.2

	Sel%-MTD	0	1.2	4.7	18	29.3	46.8		
	Sel%-RP2D	0	0	0.4	65.6	16.8	6.2		
N2	Pts(%)	3.4(9%)	4(10.7%)	5.2(13.9%)	10.1(27.2%)	8.4(22.5%)	6.3(16.8%)	37.3	19.5
	Sel%-MTD	0	1	4.3	12.1	26.9	55.7		
	Sel%-RP2D	0	0	0.2	65.8	18	7.8		
N3	Pts(%)	3.4(7%)	4(8.4%)	5.4(11.3%)	13.3(27.9%)	12(25.1%)	9.7(20.3%)	47.8	23.8
	Sel%-MTD	0	0.3	1.7	7.5	27	63.5		
	Sel%-RP2D	0	0	0.1	67.2	19.9	8.1		
N4	Pts(%)	3.4(6.1%)	4(7.3%)	5.5(9.9%)	15.9(28.9%)	14.1(25.7%)	12.2(22.1%)	55	26.4
	Sel%-MTD	0	0.3	1.5	6.4	26	65.8		
	Sel%-RP2D	0	0	0	67.9	18.6	9		
Scenario 5	DLT rate	0.01	0.03	0.06	0.1	0.12	0.25		
	Response rate	0.1	0.15	0.2	0.4	0.4	0.4		
	Mean change of QoL	-4	-8	-12	-18	-20	-20		
N1	Pts(%)	4.9(18.2%)	5.3(19.8%)	5.1(18.8%)	4.8(17.8%)	3.8(13.9%)	3.1(11.5%)	27	15.1
	Sel%-MTD	0	1.8	4.8	17.5	29.4	46.5		
	Sel%-RP2D	11.6	16.2	6.2	0.1	0.1	0.1		
N2	Pts(%)	5.7(16%)	6.3(17.7%)	5.7(15.9%)	5.4(15.2%)	6.2(17.5%)	6.3(17.8%)	35.6	19.2
	Sel%-MTD	0	1.4	4.2	12.3	27	55.1		
	Sel%-RP2D	14.1	18.5	5.2	0	0	0		
N3	Pts(%)	6.3(14.2%)	7.1(16%)	6.1(13.8%)	5.9(13.5%)	8.8(19.9%)	10(22.6%)	44.2	23.6
	Sel%-MTD	0	0.4	1.4	7.6	24.1	66.5		
	Sel%-RP2D	9.1	13.3	3.2	0	0	0		
N4	Pts(%)	6.7(13.5%)	7.6(15.4%)	6.3(12.7%)	6.2(12.6%)	10.2(20.7%)	12.3(25%)	49.3	26.1
	Sel%-MTD	0	0.4	1.3	7.7	25.2	65.4		
	Sel%-RP2D	4.8	11.2	2.7	0	0	0		

Scenario 6	DLT rate	0.03	0.06	0.12	0.25	0.4	0.55		
	Response rate	0.2	0.2	0.3	0.4	0.4	0.4		
	Mean change of QoL	-14	-14	-18	-20	-20	-20		
N1	Pts(%)	4.2(17.7%)	5.4(22.6%)	6.2(26%)	5.6(23.5%)	2(8.4%)	0.4(1.8%)	23.8	14.9
	Sel%-MTD	0.9	6.3	28	46.9	15.5	2.4		
	Sel%-RP2D	2	2.1	0.1	0	0	0		
N2	Pts(%)	4.3(14.5%)	5.7(19%)	7.8(26%)	7.8(26.2%)	3.5(11.9%)	0.7(2.4%)	29.9	18.4
	Sel%-MTD	0.8	5.9	27.1	51	14.5	0.7		
	Sel%-RP2D	0.9	1.9	0	0	0	0		
N3	Pts(%)	4.4(11.8%)	6(16.2%)	9.6(25.7%)	11.1(29.7%)	5.3(14.1%)	0.9(2.4%)	37.3	22.8
	Sel%-MTD	0.3	2.9	23.5	59.1	13.9	0.3		
	Sel%-RP2D	0.2	0.8	0	0	0	0		
N4	Pts(%)	4.4(10.8%)	6.1(14.9%)	10.6(25.8%)	13(31.7%)	6(14.6%)	0.9(2.3%)	41.1	25.2
	Sel%-MTD	0.3	2.9	25.1	60.1	11.4	0.2		
	Sel%-RP2D	0.1	0.2	0	0	0	0		
Scenario 7	DLT rate	0.03	0.06	0.12	0.25	0.4	0.55		
	Response rate	0.1	0.2	0.4	0.45	0.5	0.5		
	Mean change of QoL	-2	-4	-10	-4	-14	-18		
N1	Pts(%)	5.4(19.1%)	7.2(25.4%)	7.1(25.1%)	6.2(22%)	1.9(6.9%)	0.4(1.4%)	28.2	14.8
	Sel%-MTD	0.9	5.9	30.1	45.9	14.2	3		
	Sel%-RP2D	5.5	19.2	23.3	40.8	0.5	0.1		
N2	Pts(%)	6.3(16.7%)	8.9(23.7%)	9.5(25.4%)	9.2(24.4%)	3.2(8.5%)	0.5(1.4%)	37.6	18.3
	Sel%-MTD	0.8	5.3	30.4	50.8	11.7	1		
	Sel%-RP2D	3.9	18.7	21.6	46.5	0.2	0		
N3	Pts(%)	6.8(14.2%)	10.7(22.3%)	12.4(25.9%)	12.9(26.9%)	4.4(9.2%)	0.7(1.5%)	48.1	22.6
	Sel%-MTD	0.3	3.2	25.5	60.5	9.5	1		

	Sel%-RP2D	2.6	12.2	22.7	55.5	0.3	0		
N4	Pts(%)	7.1(13.1%)	12(22%)	14.1(25.8%)	15.4(28.2%)	5.2(9.5%)	0.8(1.4%)	54.7	25.1
	Sel%-MTD	0.3	2.7	24.5	62.7	9.2	0.6		
	Sel%-RP2D	1.5	9.2	20.6	60.2	0.2	0		
Scenario 8	DLT rate	0.1	0.25	0.4	0.55	0.65	0.65		
	Response rate	0.45	0.45	0.45	0.5	0.5	0.5		
	Mean change of QoL	-2	-6	-12	-18	-20	-20		
N1	Pts(%)	9.7(42.5%)	9(39.3%)	3.4(15%)	0.7(2.9%)	0(0.2%)	0(0%)	22.8	12.4
	Sel%-MTD	24	57.5	16.7	1.4	0	0		
	Sel%-RP2D	65	33	0.6	0	0	0		
N2	Pts(%)	13.3(45.4%)	11.1(37.8%)	4(13.8%)	0.8(2.7%)	0.1(0.2%)	0(0%)	29.3	14.7
	Sel%-MTD	26.1	58.9	13.9	0.7	0	0		
	Sel%-RP2D	65.4	32.2	0.8	0	0	0		
N3	Pts(%)	17.2(45.3%)	14.5(38.1%)	5.3(14%)	0.9(2.4%)	0.1(0.2%)	0(0%)	38	18.4
	Sel%-MTD	21.5	63.9	13.7	0.5	0	0		
	Sel%-RP2D	63	35.3	0.8	0	0	0		
N4	Pts(%)	20.3(46%)	16.9(38.2%)	6(13.6%)	0.9(2.1%)	0.1(0.1%)	0(0%)	44.2	20.6
	Sel%-MTD	22.1	66.8	9.9	0.8	0	0		
	Sel%-RP2D	64.1	34.5	0.7	0	0	0		
Scenario 9	DLT rate	0.1	0.25	0.4	0.55	0.65	0.65		
	Response rate	0.2	0.45	0.45	0.5	0.5	0.5		
	Mean change of QoL	-14	-6	-12	-18	-20	-20		
N1	Pts(%)	7.5(36.5%)	9(43.6%)	3.3(16.3%)	0.7(3.3%)	0(0.2%)	0(0%)	20.5	12.3
	Sel%-MTD	27.4	53.4	17.3	1.5	0.1	0		
	Sel%-RP2D	2.2	64.4	2.4	0	0	0		
N2	Pts(%)	8.9(35.9%)	11.1(44.8%)	3.9(15.9%)	0.8(3.2%)	0.1(0.2%)	0(0%)	24.8	14.4

	Sel%-MTD	27.4	57.5	14	0.8	0	0		
	Sel%-RP2D	1.2	66	1.4	0	0	0		
N3	Pts(%)	10.6(33.5%)	14.6(46.5%)	5.2(16.6%)	1(3.2%)	0.1(0.2%)	0(0%)	31.5	18
	Sel%-MTD	21.8	63.2	13.9	0.8	0	0		
	Sel%-RP2D	0.4	73.4	0.9	0	0	0		
N4	Pts(%)	11.5(32.7%)	16.8(47.8%)	5.7(16.3%)	1(3%)	0.1(0.2%)	0(0%)	35.2	19.8
	Sel%-MTD	22.5	65.5	11.1	0.6	0	0		
	Sel%-RP2D	0.3	73.5	0.9	0	0	0		
Scenario 10	DLT rate	0.3	0.35	0.4	0.55	0.65	0.65		
	Response rate	0.3	0.3	0.3	0.3	0.3	0.3		
	Mean change of QoL	-14	-15	-16	-18	-20	-20		
N1	Pts(%)	7.2(60.1%)	3.5(28.9%)	1.1(8.9%)	0.2(1.9%)	0(0.2%)	0(0%)	12	7.8
	Sel%-MTD	71.6	13.6	3.6	0.7	0.1	0		
	Sel%-RP2D	6.5	0.1	0.1	0	0	0		
N2	Pts(%)	9.3(63.6%)	3.8(26.1%)	1.2(8.3%)	0.3(1.8%)	0(0.2%)	0(0%)	14.6	9.3
	Sel%-MTD	67.2	12.3	3.5	0.5	0	0		
	Sel%-RP2D	4.3	0	0.1	0	0	0		
N3	Pts(%)	12(64.7%)	4.6(25%)	1.5(8.2%)	0.4(1.9%)	0(0.2%)	0(0%)	18.5	11.6
	Sel%-MTD	64.1	12.4	3.8	0.4	0	0		
	Sel%-RP2D	3	0	0	0	0	0		
N4	Pts(%)	14.3(66.9%)	5(23.5%)	1.6(7.7%)	0.4(1.7%)	0(0.2%)	0(0%)	21.4	13.4
	Sel%-MTD	62.7	11.2	3.6	0.3	0	0		
	Sel%-RP2D	2.1	0	0.1	0	0	0		

The labels N1, N2, N3 and N4 correspond to the cases where $(N_{esc}, n_{stop}, n_{cap}) = (24,6,8)$, (36,9,12), (48,12,16), and (60,15,20), respectively.
